# Identification of Cyclobutane Pyrimidine Dimer-Responsive Genes Using UVB-Irradiated Human Keratinocytes Transfected with *In Vitro*-Synthesized Photolyase mRNA

**DOI:** 10.1371/journal.pone.0131141

**Published:** 2015-06-29

**Authors:** Gábor Boros, Edit Miko, Hiromi Muramatsu, Drew Weissman, Eszter Emri, Gijsbertus T. J. van der Horst, Andrea Szegedi, Irén Horkay, Gabriella Emri, Katalin Karikó, Éva Remenyik

**Affiliations:** 1 Department of Dermatology, Faculty of Medicine, University of Debrecen, Debrecen, Hungary; 2 Department of Neurosurgery, University of Pennsylvania, Philadelphia, Pennsylvania, United States of America; 3 Department of Medicine, University of Pennsylvania, Philadelphia, Pennsylvania, United States of America; 4 Department of Genetics, Erasmus University Medical Center, Rotterdam, The Netherlands; 5 Department of Dermatological Allergology, Faculty of Medicine, University of Debrecen, Debrecen, Hungary; San Gallicano Dermatologic Institute, ITALY

## Abstract

Major biological effects of UVB are attributed to cyclobutane pyrimidine dimers (CPDs), the most common photolesions formed on DNA. To investigate the contribution of CPDs to UVB-induced changes of gene expression, a model system was established by transfecting keratinocytes with pseudouridine-modified mRNA (Ψ-mRNA) encoding CPD-photolyase. Microarray analyses of this model system demonstrated that more than 50% of the gene expression altered by UVB was mediated by CPD photolesions. Functional classification of the gene targets revealed strong effects of CPDs on the regulation of the cell cycle and transcriptional machineries. To confirm the microarray data, cell cycle-regulatory genes, *CCNE1* and *CDKN2B* that were induced exclusively by CPDs were selected for further investigation. Following UVB irradiation, expression of these genes increased significantly at both mRNA and protein levels, but not in cells transfected with CPD-photolyase Ψ-mRNA and exposed to photoreactivating light. Treatment of cells with inhibitors of c-Jun N-terminal kinase (JNK) blocked the UVB-dependent upregulation of both genes suggesting a role for JNK in relaying the signal of UVB-induced CPDs into transcriptional responses. Thus, photolyase mRNA-based experimental platform demonstrates CPD-dependent and -independent events of UVB-induced cellular responses, and, as such, has the potential to identify novel molecular targets for treatment of UVB-mediated skin diseases.

## Introduction

The incidence of keratinocyte-derived skin cancer, which is the most common human malignancy, continues to increase worldwide, thus presenting a serious challenge to healthcare systems [1]. Ultraviolet B (UVB) (290–320 nm) radiation is the main environmental risk factor for sunburn, skin carcinogenesis and premature skin ageing [2,3]. Cyclobutane pyrimidine dimers (CPDs) are the predominant photolesions caused by UVB radiation, and primarily they are responsible for these adverse effects [4]. CPDs are the most deleterious and premutagenic photolesions, due to their ability to distort the structure of the DNA, leading to disturbance of DNA replication and transcription [5,6]. The pathogenetic role of CPDs is further substantiated by presence of CPD-related signature mutations in genes involved in the formation of skin cancers [7], as well as, by the correlation between the action spectrum value for the induction of CPD photolesions and development of UV-induced skin cancer in animal models [8,9]. In addition, CPDs have been shown to mediate UVB-induced erythema [10] and immunosuppression [11,12]. Naturally, DNA lesions, including CPDs are excised by the nucleotide excision repair (NER) system of human keratinocytes [13]. However, the rate and accuracy of DNA repair by NER are suboptimal [14].

CPD-photolyase is a structure-specific DNA repair enzyme that specifically binds and cleaves CPDs using the energy of visible light (“photoreactivation”), thereby simply and rapidly restoring DNA integrity [15]. This enzyme functions in diverse organisms from bacteria to vertebrates but is absent in placental mammals, including humans, that must rely solely on the less potent NER to repair UV-induced DNA lesions [16]. Sunscreen lotions containing liposomal-encapsulated bacterial photolyase or CPD-specific endonuclease have been marketed for preventing UV-induced skin damages [17], especially in patients with NER-deficiency [18].

In a previous study, we applied a novel mRNA-based gene delivery method, and demonstrated that transfection of pseudouridine-modified mRNA (Ψ-mRNA) encoding *Potorous tridactylus* CPD-photolyase (CPD-PL) into human keratinocytes leads to rapid repair of DNA-damage [19]. Pseudouridine modifications increase mRNA stability [20], make it highly translatable [21,22] and abolish immunogenicity of the RNA [23]. It is well documented that CPD lesions are considered to be the principal mediator of UV-induced mutagenesis and DNA double-strand break (DSB) signalling [7,9]. However, so far, it has been unclear how CPDs change gene expression and cell activities. To gain insight, we performed a global analysis (microarray) of molecular networks. Most dermatological studies, in which microarray technology was used, analysed differential expression of genes comparing normal and pathologic skin samples in order to identify genes associated with a specific skin condition or with tumor progression [24–28]. Microarray platforms were also used to identify UV-regulated genes and have uncovered that significant change in the expression profiles of hundreds of genes are induced by UV. Altered expression of genes in response to UV irradiation have been determined in epidermal keratinocytes [29], fibroblasts [30] and melanocytes [31]. Microarray experiments have demonstrated that UVB exposure affects several biological processes indicating the complexity of UV-induced cellular activities. Studies performed on human keratinocytes identified UVB-induced genes that were involved in proteasome-mediated pathways, cytoskeleton organization, cell cycle and apoptosis networks, and control of basal transcription and translation leading to inhibition of cell growth [29,32–34]. Furthermore, it has been shown that the repair rate of DNA lesions alters the UV-induced transcription profile, thus suggesting that adequate removal of the photoproducts could avoid UV-related cutaneous pathologies [35]. However, until now, there was no suitable experimental platform to identify directly CPD-responsive genes in human cells, thus distinguish CPD-regulated cellular mechanisms from those mediated by other UVB-induced derivatives, including diverse photoproducts, reactive oxygen species, cross-linked protein-DNA and other damaged macromolecules.

Here, we present data obtained by using human keratinocytes transfected with *in vitro*-synthesized nucleoside-modified mRNA encoding a marsupial-specific CPD-photolyase. This model system enabled us to distinguish between CPD-dependent and-independent cellular processes. We determined the UVB-induced transcriptional responses in human keratinocytes using the combination of a CPD-specific photolyase Ψ-mRNA delivery with a genomics approach. Pathway analysis revealed a strong effect of CPDs on the expression levels of genes involved in the control of the cell cycle and transcriptional machineries. Our findings demonstrate that the c-Jun N-terminal kinase (JNK) signalling pathway plays a role in conversion of the cues generated by UVB-induced CPDs into a transcriptional response.

## Materials and Methods

### RNA synthesis

Messenger RNAs were generated as previously described [19], using linearized plasmids (pTEV-CPD-PL-A101 and pTEVeGFP-A101) encoding codon-optimized *Potorous* CPD-photolyase (CPD-PL Ψ-mRNA) and enhanced green fluorescent protein (eGFP Ψ-mRNA). The CPD-photolyase gene from *Potorous tridactylus* (rat kangaroo) was synthesized by Entelechon (Bad Abbach, Germany). The Megascript T7 RNA polymerase kit (Ambion, Austin, TX) was used for transcription, and UTP was replaced with pseudouridine triphosphate (TriLink, San Diego, CA) [21]. To remove the template DNA Turbo DNase (Ambion) was added to the reaction mix. Pseudouridine-modified mRNAs were HPLC-purified as described [36] and provided with cap1 generated by using the m7G capping enzyme and 2′-*O*-methyltransferase according to the manufacturer (CellScript, Madison, WI). The mRNAs were transcribed to contain 101 nt-long 3’ poly(A) tail. Small aliquots of RNA samples were stored in siliconized tubes at -20°C.

### Keratinocyte cell line

An established HaCaT cell line [37] was maintained in high glucose DMEM (Lonza, Verviers, Belgium) supplemented with 2 mM L-glutamine (Lonza), 10% fetal bovine serum (Lonza) and 0.5% antibiotic/antimycotic solution (Sigma-Aldrich, St. Louis, MO, USA) at 37°C with 5% CO_2_.

### Transient transfection and treatments

Transient transfection of Ψ-mRNA into HaCaT cells, UVB exposure and photoreactivation experiments were carried out, as previously described [19], except that a ratio of mRNA (0.25 μg) and Lipofectamine LTX-PLUS (Life Technologies) reagent (1.0 μl) in a final volume of 100 μl EpiLife keratinocyte growth medium (Life Technologies, Carlsbad, CA, USA) was used. Following a two-hour transfection, the lipofectamine-RNA complex was replaced with 100 μl fresh culture medium. At 12 h post transfection, the cell monolayer in a well of a 96-well plate, was covered with 50 μl Dulbecco’s phosphate buffer saline (Life Technologies) and subjected to 20 mJ/cm^2^ UVB using two TL-20W/12 tubes (Philips). Proper dosage of UVB was determined by a UVX Digital Radiometer (UVP Inc., San Gabriel, CA, USA). Immediately after UVB treatment, cells were exposed to visible light (“photoreactivation”) using F18W Daylight fluorescent tubes (Sylvania) or kept in the dark for one hour. At 5 and 23 h following photoreactivation, total RNA and genomic DNA were isolated from the cells.

### Enzyme-linked immunosorbent assay (ELISA)

Genomic DNA was extracted from HaCaT cell line using the Qiagen Blood and Cell Culture kit (Qiagen, Hilden, Germany), according to the manufacturer. A direct ELISA was applied for detection of CPD, as previously described [19]. Anti-CPD monoclonal antibody (TDM-2, Cosmo Bio, Tokyo, Japan) diluted in PBS (1:1000) was used as primary antibody.

### Microarray analysis

Total RNA was isolated from HaCaT cells using Trizol reagent (MRC, Cincinnati, OH, USA), according to the manufacturer’s instructions. RNA samples were treated by DNase I (Fermentas, St. Leon-Rot, Germany) and their quality were analyzed using an Agilent 2100 Bioanalyzer (Agilent Technologies, Santa Clara, CA, USA). Gene expression profiling of HaCaT cells transfected with CPD-photolyase mRNA was performed using a service provider (ChromoScience, Budapest, Hungary, http://www.chromoscience.hu). Briefly, cyanine 3-labeled cRNA was synthesized from 200 ng total RNA by the QuickAmp Labeling Kit (Agilent Technologies), according to the manufacturer. For each sample, 1650 ng of one-color labeled cRNA was hybridized to an Agilent 4x44 K Whole Human Genome Oligo (Agilent Technologies) microarray platform at 65°C for 17 h. All further steps were carried out according to the manufacturer (Agilent Technologies). The slides were scanned with the Agilent Microarray Scanner. Data were then normalized by the Feature Extraction software version 9.5 (Agilent Technologies) with default settings for one-color oligonucleotide microarrays and then transferred to GeneSpringGX program (Agilent Technologies) for further statistical evaluation. In GeneSpring, the normalization and data transformation steps recommended by Agilent Technologies for one-color data were applied at each time point. Microarray data have been submitted to Gene Expression Omnibus (GEO) [38] database and can be retrieved at http://www.ncbi.nlm.nih.gov/geo/query/acc.cgi?acc=GSE65034 (GEO Series accession number: GSE65034). Three replicates of the following 3 types of CPD-PL Ψ-mRNA transfected samples were studied by the microarray: non-irradiated, UVB-irradiated without photoreactivating light (inactive CPD-photolyase), UVB-irradiated plus photoreactivated (active CPD-photolyase). Genes showing statistically significant 2-fold change were selected for further study by Ingenuity Pathway Analysis (IPA, Qiagen; http://www.ingenuity.com/products/ipa) and Database for Annotation, Visualization and Integrated Discovery (DAVID; http://david.abcc.ncifcrf.gov) gene functional classification tool.

### Real-time quantitative RT-PCR analysis

To validate the microarray data, real-time quantitative reverse transcription (RT)-PCR was used. RT was performed using the High-Capacity cDNA Reverse Transcription Kit (Life Technologies), according to the manufacturer. RT reactions contained 500–500 ng of RNA. To quantitate the expression of candidate genes, the following TaqMan Gene Expression assays (Life Technologies) were used: *ATF3* (Hs00231069_m1), *CCNE1* (Hs01026536_m1), *CDKN2B* (p15INK4b) (Hs00793225_m1), *EGR1* (Hs00152928_m1), *ID2* (Hs04187239_m1), *PTGS2 (COX-2)* (Hs00153133_m1), *RUNX1* (Hs00231079_m1), *SNAI2* (Hs00950344_m1), the sequences are proprietary and not released by the company. To determine mRNA expression of *SNAI1* the following custom-designed primers and probe set were used: Forward primer: 5’-ACT ATG CCG CGC TCT TTC-3’; Reverse primer: 5’-GCT GGA AGG TAA ACT CTG GAT-3’; and the probe sequence is: 5’-[6-carboxyfluorescein (FAM)] AAT CGG AAG CCT AAC TAC AGC GAG C [tetramethylrhodamine (TAMRA)]-3’. The composition of RT mixes, the PCR reactions and the RT-PCR protocols were carried out as previously described [19] using the ABI 7900 HT Sequence Detection System (Life Technologies). Relative RNA expression values were calculated using the 2^-ΔΔCt^ method [39] in which expression levels in samples containing active CPD-photolyase were compared to those containing inactive one. *SDHA* ((Hs00188166_m1) and *PGK1* (Hs00943178_g1) mRNA levels that showed the smallest variation upon UVB exposure in keratinocytes [40] were used for normalization.

### Western blot analysis

For Western blot analyses, whole cell extracts were prepared, as previously described [19]. Proteins (10 μg) were separated on 12% polyacrylamide gels and transferred to nitrocellulose membranes (BioRad, Berkeley, CA, USA). After blocking the membranes in 5% non-fat dry milk, the membranes were incubated with primary antibodies diluted (1:750) in 5% skimmed milk at 4°C overnight, then with the appropriate secondary antibodies (1:3000) at room temperature for 1 hour. The following primary antibodies were used: anti-CCNE1 (4129), anti-ß-actin (3700) (Cell Signaling Technology, Danvers, MA, USA) and anti-CDKN2B (p15INK4b) (MA1-12294, Thermo Fisher Scientific, Rockford, IL, USA). Goat anti-mouse or anti-rabbit IgG conjugated with horseradish peroxidase (HRP) was used as a secondary antibody (BioRad). The visualization of proteins was achieved with ECL Prime Western blotting detection system (GE Healthcare, Little Chalfont, UK) and densitometry was performed using ImageJ public software (NIH, Bethesda, MD, USA).

### Inhibitor treatment

Keratinocytes at 85% confluency were transfected with lipofectamine-complexed CPD-PL Ψ-mRNA. Twelve hours later, cells were pretreated with specific inhibitors of JNK (SP600125), p38 MAPK (SB203580) or AKT (MK-2206) at a final concentration of 40 μM, 10 μM and 10 μM, respectively for 1 hour before treatment with UVB and subsequent photoreactivation. All chemical agents were purchased from SelleckChem (Selleck Chemicals, Houston, TX, USA) and were dissolved in dimethyl sulfoxide (DMSO) (Sigma-Aldrich). Stock solutions were prepared according to the manufacturer and stored at -20°C.

### Statistical evaluation

Identification of genes differentially expressed in microarray experiments was carried out by the unpaired, Student’s *t*-test followed by Benjamini-Hochberg correction. Statistical analysis of qRT-PCR data was performed using GraphPad Prism 5 software (GraphPad Software Inc., San Diego, CA, USA). The significance of differences in terms of mRNA expression comparing UVB-irradiated samples to non-irradiated ones, or UVB-irradiated plus photoreactivated (active CPD-photolyase) samples to non-photoreactivated (inactive CPD-photolyase) samples, respectively, was determined by the two-tailed, unpaired *t*-test. A *p* value of equal to or less than 0.05 was considered statistically significant.

## Results

### Accelerated removal of CPD lesions in keratinocytes transfected with CPD-photolyase mRNA

**Fig 1:**
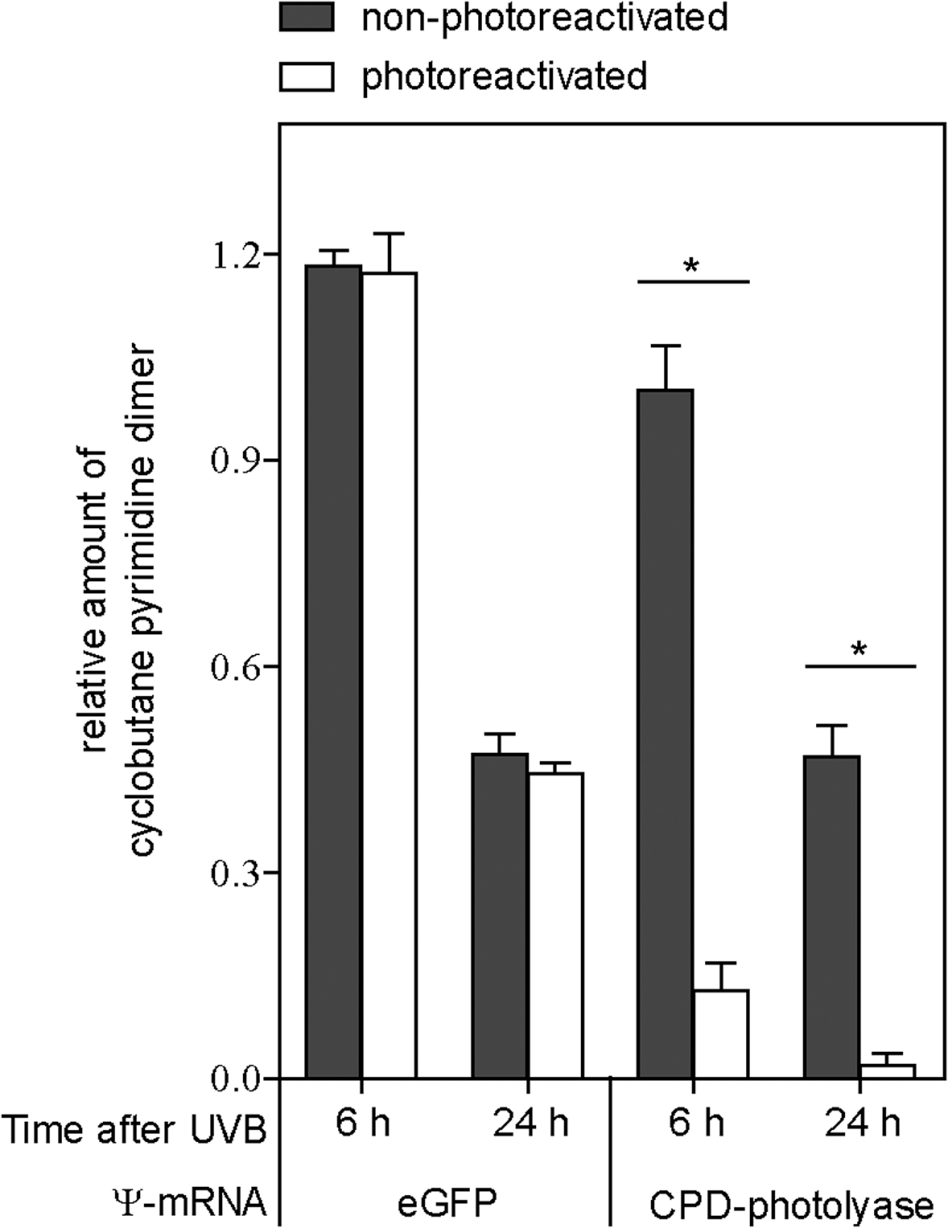
Accelerated photorepair of CPDs in HaCaT cells transfected with CPD-PL Ψ-mRNA. HaCaT cells were transfected with lipofectamine-complexed Ψ-mRNA encoding CPD-photolyase. Twelve hours later, cells were subjected to 20 mJ/cm^2^ UVB and immediately exposed to photoreactivating light (photoreactivated) or left in the dark (non-photoreactivated) for 1 h and then maintained at 37°C for 5 and 23 hs. After incubation at 5 and 23 h, genomic DNA was isolated at the indicated times after UVB irradiation and the amount of CPDs was measured by ELISA. The values were calculated relative to those obtained with cells that were not UVB-irradiated. Significance was assessed by unpaired, two-sample *t*-test, p<0.05. Error bars represent the standard error of the mean from three experiments performed independently.

To test the enzymatic activity of CPD-photolyase translated in HaCaT cells from the transfected mRNA (CPD-PL Ψ-mRNA), cells were subjected to a physiological dose of UVB (20 mJ/cm^2^) 12 h after transfection of lipofectamine-complexed CPD-PL Ψ-mRNA. Immediately thereafter, cells were either exposed to photoreactivating light, or left in the dark for 1 hour. Cells were harvested 5 and 23 hours later, and CPD-specific ELISA was performed to measure the amount of CPDs formed in genomic DNA. The amount of CPDs in CPD-PL Ψ-mRNA transfected cells was reduced by 90% in those receiving photoreactivating light (photoreactivated), as compared to cells kept in the dark (non-photoreactivated) (Fig 1). Cells transfected with control eGFP Ψ-mRNA contained the highest amount of CPD lesions at both 6 and 24 h after UVB irradiation (Fig 1). A reduced amount of CPD lesions were measured in all irradiated samples at 24 h vs. at 6 h due to slower endogenous NER-mediated DNA repair.

### CPD-dependent changes were distinguished within the UVB-induced alterations of gene expression in human keratinocytes

To determine the CPD-dependent gene expression profile of human keratinocytes exposed to UVB irradiation, an oligonucleotide-based DNA microarray analysis was performed in which HaCaT cells transfected with CPD-PL Ψ-mRNA were used. Total RNA was isolated at 6 and 24 h after UVB exposure and subsequent photoreactivation (or not). Gene expression values of UVB-irradiated plus photoreactivated samples (active CPD-photolyase) were compared to non-irradiated samples and to those that were UVB irradiated, but left without photoreactivation (inactive CPD-photolyase). UVB irradiated, but non-photoreactivated samples were also compared to non-irradiated samples. Cut-off values for changes in gene expression were set at ± 2-fold, and according to this criterion, expression levels of 2,370 out of 41,000 genes were changed at 6 or 24 h after UVB exposure. Altered expression of 1,334 (56%) of UVB-regulated genes was restored in cells that contained active CPD-photolyase indicating that the changes in the expression of these genes were CPD-dependent (S1 Fig). Importantly, more CPD-dependent gene expression changes (1,008 out of 1,334 genes) were observed at 6 h vs. 24 h, potentially due to the higher difference in CPD levels at 6 h comparing cells with active CPD-photolyase to those with inactive one (S1 Fig, S1 and S2 Tables). There are other reasons, such as transient expression of induced genes. On the other hand, CPD-dependent genes represented more than half of the UVB-regulated genes (1008 out of 1743 genes and 326 out of 627 genes at 6 and 24 h after UVB irradiation, respectively) at both time points after the exposure (S1 Fig, S1 and S2 Tables). These data suggest that UVB-induced CPDs mediate a significant portion of the transcriptional alterations after 6 and 24 hours in response to UVB irradiation of human keratinocytes.

### UVB-induced, CPD-dependent genes are associated with cellular stress responses

To determine possible network interactions and associated biological functions of CPD-regulated genes, datasets representing all the differentially expressed genes derived from microarray analyses were imported into IPA and DAVID software. Using these applications, we determined that a majority of CPD-dependent genes at 6 h after UVB exposure belong to the regulatory network of *Cell Cycle and Gene Expression*, while those at the 24 h belong to the network of *Cellular Development*, *Growth and Proliferation* (S2A Fig, S3A–S3G Fig). The top-rated network of CPD-regulated genes at 6 h after UVB irradiation was centered around a proto-oncogene *(c-Jun)*; the prostaglandin-endoperoxide synthase 2 *(PTGS2)*, which is a major mediator of inflammation, and the glucocorticoid receptor *(NR3C1)*, which mainly functions as a transcription factor (S2B Fig). In addition, c-Jun and interleukin 6 *(IL6)*, the transcriptional regulator nuclear factor kappa B *(NF-κB)*, *p38* and the protein kinase *ERK* were central nodes in the top network of CPD-dependent genes at 24 h after UVB exposure (S2C Fig). 122 out of the 1008 CPD-regulated genes belonged to the best-scored IPA network at 6 h after UVB and 60 out of the 326 CPD-regulated genes at 24 h after the exposure (S2A Fig). Functional classification of CPD-regulated genes within networks (S2B and S2C Fig) suggested that a wide range of cell cycle-regulatory genes, positive or negative regulators of the transcriptional and cell recovery machineries, proto-oncogenes, apoptosis and protein kinase related genes were uniquely induced or repressed by CPD photolesions (S3A–S3G Fig). Nine genes regulated by CPDs were selected for further investigation based on the microarray data and their involvement in cell cycle regulation *(CCNE1*, *CDKN2B)*, transcriptional regulation *(ATF3*, *EGR1*, *ID2*, *RUNX1)*, epithelial to mesenchymal transition *(SNAI1*, *SNAI2)* and inflammation *(PTGS2)* (Table 1).

**Table 1:** Summarized fold change values by microarray results of the selected 9 CPD-dependent genes.

			6 h after UVB irradiation		24 h after UVB irradiation	
GenBank ID	Gene	Description	vs. non-irradiated	active PL vs. inactive PL	vs. non-irradiated	active PL vs. inactive PL
NM_001040619	ATF3	activating transcription factor 3	5.65	-3.30	1.85	-1.90
NM_001238	CCNE1	cyclin E1	2.87	-2.14	1.17	1.13
NM_004936	CDKN2B	cyclin-dependent kinase inhibitor 2B	7.76	-3.36	2.05	-2.06
NM_001964	EGR1	early growth response 1	2.51	-3.95	3.38	-2.39
NM_002166	ID2	inhibitor of DNA binding 2	4.35	-3.89	-2.09	1.45
NM_000963	PTGS2	prostaglandin-endoperoxide synthase 2	6.75	-3.72	4.66	-3.04
NM_001001890	RUNX1	runt-related transcription factor 1	-4.45	4.36	1.05	1.09
NM_005985	SNAI1	snail family zinc finger 1	6.44	-3.44	2.76	-2.59
NM_003068	SNAI2	snail family zinc finger 2	2.39	-2.15	1.59	-1.24

active PL: samples containing active CPD-photolyase, inactive PL: samples containing inactive CPD-photolyase.

### CPD-dependency of transcriptional changes of 9 genes selected on the basis of microarray data were confirmed by RT-qPCR

**Fig 2:**
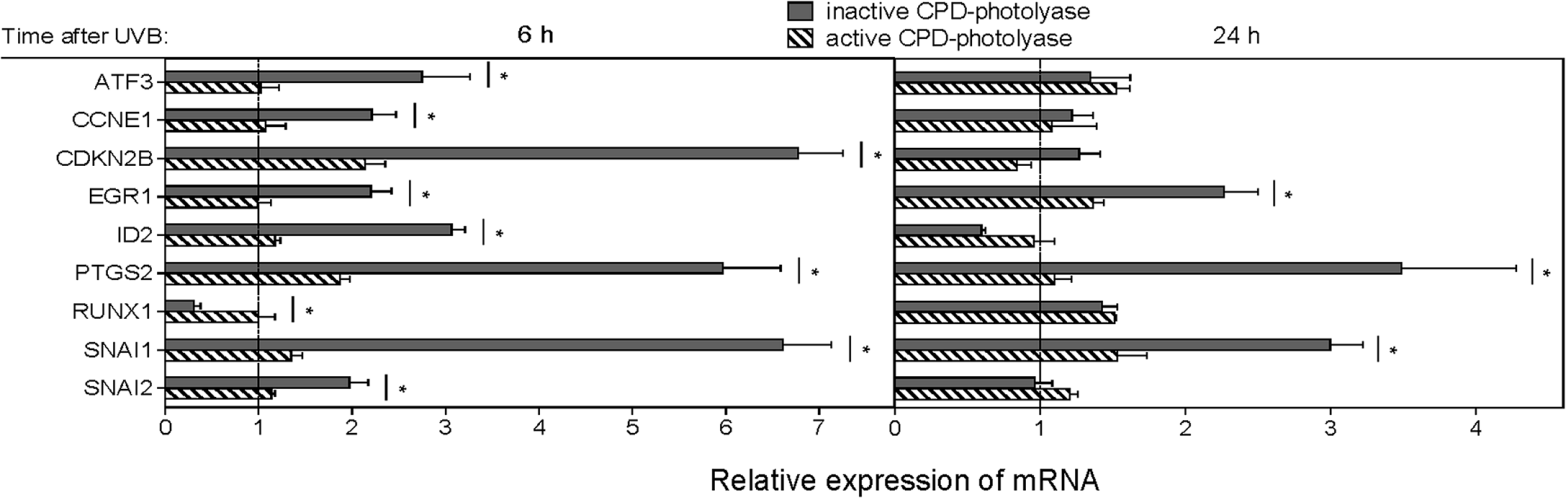
Experimental verification of microarray results for 9 selected genes. HaCaT cells were exposed to 20 mJ/cm^2^ UVB at 12 h after delivery of CPD-PL Ψ-mRNA. Immediately thereafter, cells were either subjected to photoreactivating light (active CPD-photolyase) or left in the dark (inactive CPD-photolyase) for 1 h. Following incubation total RNA was extracted at 5 and 23 h, then real-time RT-qPCR was performed to validate the CPD-dependent expression of ATF3, CCNE1, CDKN2B, EGR1, ID2, PTGS2, RUNX1, SNAI1 and SNAI2. Values measured in UVB irradiated cells with or without photoreactivation were related to those measured in non-UVB irradiated cells that were transfected control Ψ-mRNA, (pecked lines). Asterisks indicate significant differences (two-tailed, unpaired *t*-test; p<0.05) between photoreactivated (active CPD-photolyase) and non-photoreactivated (inactive CPD-photolyase) samples. The results of RT-qPCR are means ± SEM from three independent experiments in triplicate.

To validate the microarray data, we employed real time quantitative RT-PCR analyses. Regarding *ATF3*[41], *EGR1*[41], *ID2*[42], *SNAI1*[43], *SNAI2*[43], *PTGS2*[44] and *CCNE1*[45] UV-induced upregulation of these genes has been previously described [41–45], and our results were in agreement with the literature. We found significant upregulation of the expression of *CDKN2B* (p15INK4b), and downregulation of the expression of *RUNX1* in response to UVB (Fig 2). Furthermore, presence of active CPD-photolyase significantly prevented the changes in the expression of all tested genes (Fig 2), thereby confirming the results of the microarray analysis.

### Overexpression of cyclin E1 *(CCNE1)* and p15INK4b *(CDKN2B)* proteins in response to UVB is dependent on the generation of CPD and activation of JNK pathway

**Fig 3:**
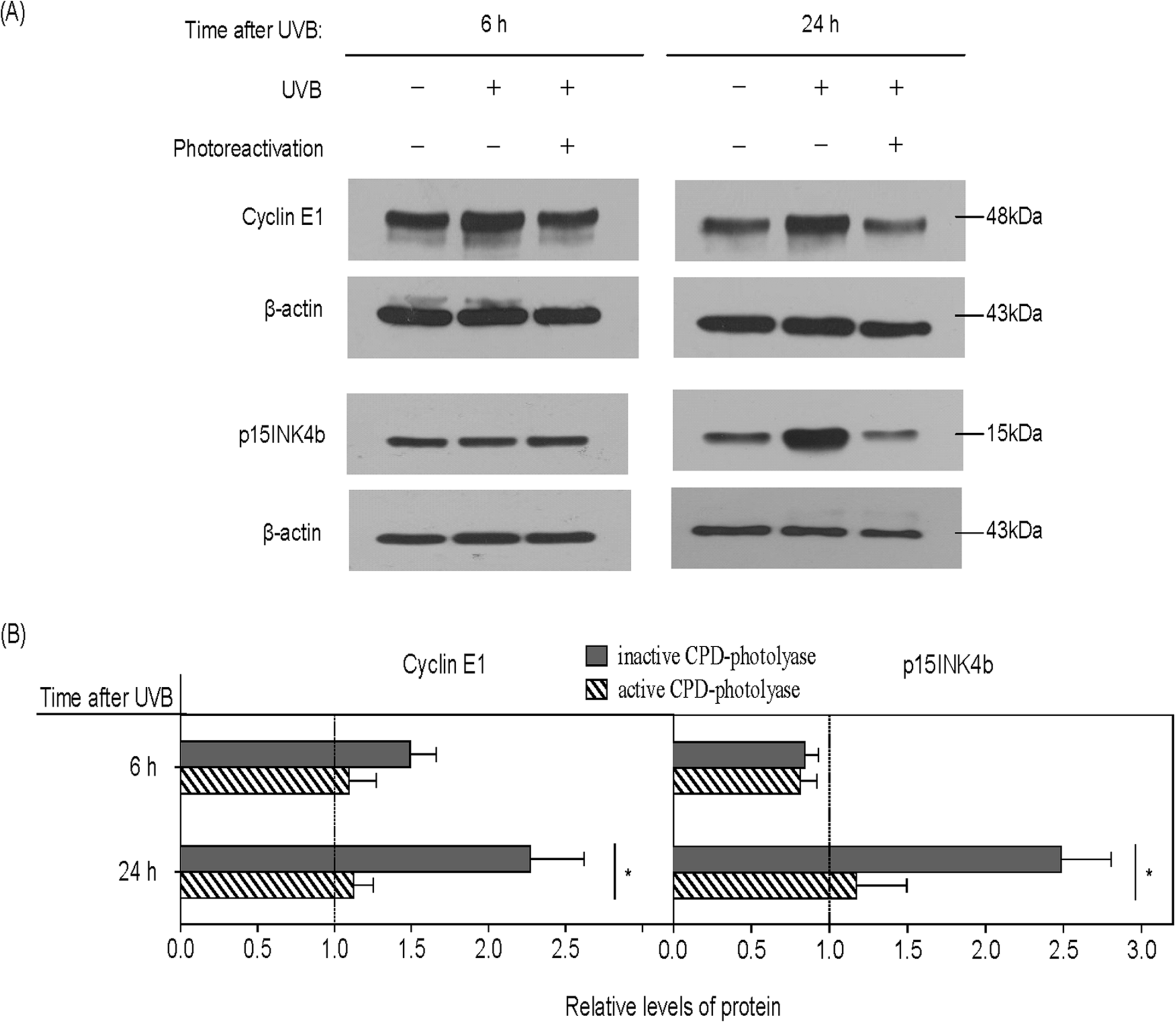
Photorepair of CPDs prevents altered expression of cyclin E1 and p15INK4b protein in UVB irradiated HaCaT cells. Cells were transfected with lipofectamine-complexed CPD-PL Ψ-mRNA, 12 hs later irradiated with 20 mJ/cm^2^ UVB and immediately exposed to photoreactivating light (active CPD-photolyase) or kept in the dark (inactive CPD-photolyase) for 1 h. Subsequently, cells were cultured at 37°C until harvested at the indicated time after UVB irradiation. (A) The expression of cyclin E1 and p15INK4b were analyzed by Western blot. (B) Quantitation of western blots displays relative changes in protein expression normalized to β-actin. Pixel densities were calculated relative to those obtained with cells that were not UVB irradiated (pecked lines). Significance was assessed by two-tailed, unpaired *t*-test (asterisk, p<0.05) showing differences between photoreactivated and non-photoreactivated samples. Error bars represent the standard error of the mean. The results are means of three independent experiments.

For further investigation, we selected two cell cycle-regulatory genes that were less characterized, related to their involvement in UVB-induced stress response. We have found that the expression of cyclin E1 and p15INK4b proteins were significantly increased at 24 h after UVB irradiation, but this increase was prevented by active CPD-photolyase (Fig 3A and 3B).

**Fig 4:**
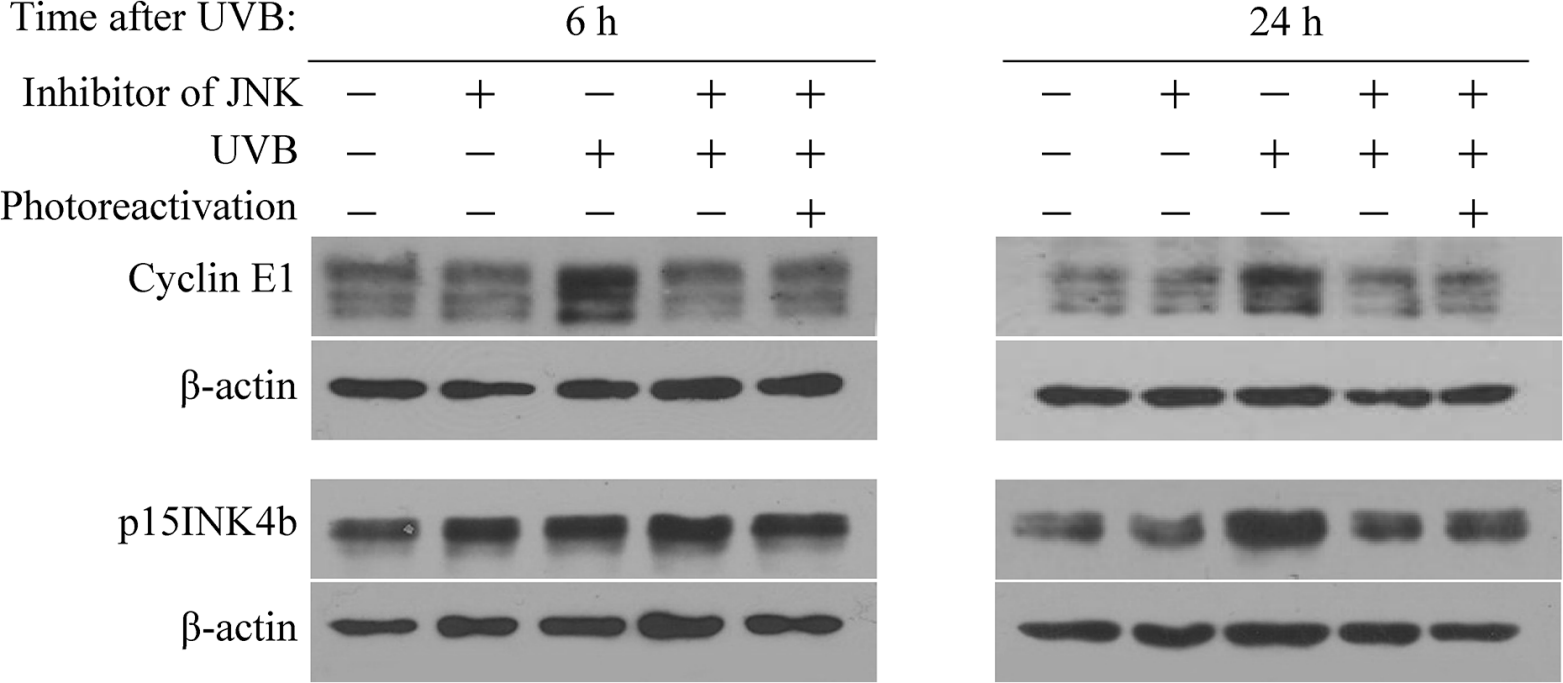
Induction of cyclin E1 and p15INK4b protein expression upon UVB exposure is regulated through the JNK signalling pathway in HaCaT cells. Keratinocytes transfected with CPD-PL Ψ-mRNA were incubated in serum-free medium supplemented with JNK inhibitor (SP600125) for 1 h. Immediately thereafter, cells were irradiated with a physiological dose of UVB or left untreated followed by exposure to photoreactivating light (or not) for 1 h. The cells were cultured further in serum-free medium supplemented with the inhibitor. Cells were harvested for western blot assay at the indicated time. Protein levels of cyclin E1, p15INK4b, and β-actin are noted. The figure shows representative results from three independent experiments.

UVB can activate various protein kinases to regulate cell proliferation and survival pathways in human keratinocytes [46]. To investigate the signalling pathways involved in modulation of cyclin E1 and p15INK4b expression upon UVB exposure, HaCaT cells transfected with CPD-PL Ψ-mRNA were first exposed to specific inhibitors of JNK (SP600125), p38 MAPK (SB203580) or AKT (MK-2206) for 1 h, then immediately subjected to 20 mJ/cm^2^ of UVB and subsequent photoreactivating light (active CPD-photolyase) or not (inactive CPD-photolyase). After photoreactivation, cells were cultured in keratinocyte medium containing inhibitor until harvested at 6 and 24 h. We determined the expression of cyclin E1 and p15INK4b proteins by western blot analysis. While suppression of p38 MAPK or AKT had no effect on UVB-induced increase in the levels of these proteins (data not shown), we found that the treatment of cells with the JNK inhibitor SP600125 abolished the induction of cyclin E1 and p15INK4b protein expression at 24 h after UVB (Fig 4). Taken together, these results indicate that activation of JNK could be responsible for transducing the signal from CPD lesions towards the induction of *CCNE1* and *CDKN2B* gene expression in UVB-exposed human keratinocytes.

## Discussion

The use of *in vitro*-transcribed messenger RNA for delivery of genetic material into mammalian cells has grown into a novel and alternative tool to oust DNA-based gene transfer in the last decade [47]. It is well documented that cells transfected with mRNA can synthesize the encoded protein [47]. We have previously shown that human cultured keratinocytes expressing CPD-photolyase from transfected *in vitro*-synthesized mRNA are suitable for investigating the cellular and molecular effects of UVB-induced CPDs [19]. Optimization of the transfection procedure resulted in 90% removal of CPDs within the first hour of photoreactivation, making this platform highly suitable for identification of CPD-dependent gene expression changes after UVB exposure. UV-induced mutagenesis is attributed to CPDs, which under normal conditions are repaired by the slow NER system [14]. Mutations in NER genes result in a less effective and error-prone DNA repair, which increases the probability of developing cancer [48]. Identification of the genes regulated by CPD photolesions might have clinical relevance, as it will facilitate defining novel targets for diagnosis or treatment of UVB-mediated skin diseases. We identified 1334 CPD-responsive genes, which represent more than 50% of the genes regulated by UVB at 6 and 24 h after the exposure, indicating that CPDs are major contributors to altered gene expression induced by UVB irradiation, as well as, to the harmful effects of UVC exposure [49]. These data also indicate that transcriptional change of a significant number of genes remained the same at 6 or 24 h after UVB exposure and subsequent photoreactivation in cells transfected with Ψ-mRNA encoding CPD-specific photolyase. This confirms that other UVB-related factors also play a role in gene regulation, but are independent from CPDs. Such factors include 6–4 photoproduct (6-4PP), which is the second most abundant UV-induced photolesion formed in DNA [50]. However, the removal of these photoproducts from the mammalian genome is very fast [14], thus 6-4PPs could potentially contribute to the early wave of UV-induced transcriptional response [49]. In addition to 6-4PPs, other UVB-induced products, including reactive oxygen species, oxidized nucleobases (e.g. 8-oxo-7,8-dihydroxyguanine), crosslinked protein-DNA are likely to be responsible for CPD-independent transcriptional changes in human keratinocytes. A significant number of CPD-responsive genes (738 and 250 genes at 6 h and 24 h after the exposure, respectively) were downregulated, most likely due to the stalling of RNA polymerase II and inhibition of transcription elongation caused by UVB-induced DNA damage [51]. Furthermore, we found a markedly higher number of CPD-dependent genes to show altered expression levels at 6 h, as compared to 24 h.

Herein, we mainly focused on analysis of genes regulated by UVB-induced CPD lesions at 6 h after the exposure. Top functional categories were determined among these genes, and we found that CPD-related gene expression changes were mainly associated with regulation of the cell cycle and transcriptional machineries in response to UVB irradiation of human keratinocytes. Molecular studies have provided evidence that recognition of DNA damage, including UVB-induced photolesions, can temporarily halt cell cycle progression, allowing time for cells to repair damages prior to replication [52,53]. Unsuccessful repair of these photoproducts initiates intracellular apoptotic signalling in order to prevent the multiplication of mutated chromosomes, but may lead to a permanent cell cycle block, genomic instability or skin carcinogenesis [3,9,12,54]. The importance of cell cycle arrest is supported by studies in which the function of genes encoding cell cycle inhibitors (INK4 and Cip/Kip family) and cell cycle regulators (p53, Rb) were analysed [55–58]. A strong correlation has been demonstrated between the occurrence of CPD-caused “UV fingerprint” mutations in p53 gene and the development of skin cancer [59]. In addition, p53 was also shown to be regulated by UVB at a posttranslational rather than at the transcriptional level [60]. And that might be the reason, why we did not detect a change in the expression of p53 in cells exposed to UVB (GEO database—GSE65034), but observed CPD-dependent changes in mRNA levels of several p53 target genes, including *MDM2*, *ATF3*, *CCNE1*, *SNAI1*, *CDK7*, *WWOX* (Fig 2, S1 and S2 Tables). These data demonstrated that germline mutations, chemical inactivation or alteration of cell cycle key regulators, including p53, result in perturbations of cell cycle checkpoint control leading to the transformation of the cells. Therefore, adequate regulation of cell cycle arrest is critical to eliminate mutated and potentially malignant cells. According to our microarray data, CPDs are the primary cause of UVB-induced cell cycle arrest. This finding is further supported by data demonstrating that photolyase-dependent removal of CPDs prevents UVB-induced upregulation of negative regulators of cell cycle, including *BTG2*, *CDKN2B* (p15), *CDKN1C* (p57), *GADD45B*, and *RGS2* (S3A Fig). Our microarray experiment provides new data that CPDs are the primary cause of UVB-induced cell cycle arrest [52].

Among the regulated cell cycle-related genes, *CDKN2B*, which is involved in G1/S checkpoint control of cell cycle as well as gain and loss events for p15INK4b have been extensively studied in recent years [61,62] with less established roles in UVB-mediated cellular damage in keratinocyte biology. Results of our RT-qPCR and western blotting analyses showed that the CPD-photolyase mediated repair of UVB-induced CPDs prevented increased expression of *CDKN2B* (p15INK4b) mRNA and protein. Loss of p15INK4b function and *CDKN2B* mutations has been described in several types of tumors [57,61–63] confirming that the inactivation of p15INK4b significantly contributes to the transformation of normal cells into cancer cells. Mutations and deletions in p15INK4b have been demonstrated in melanoma tumorigenesis [64], and considered to be a heightened risk of melanoma according to several wide-range examinations [65,66]. Altered expression of *CDKN2B* (p15INK4b) mRNA level and new mutations have been detected in patients with actinic keratosis (AK) suggesting its possible role in AK development [67]. Taken together, our data strongly suggest the high relevance of CPDs in disruption of cell cycle progression.

In addition to cell cycle arrest, UVB exposure may affect the cell cycle machinery and cell proliferation via regulation of target genes involved in apoptosis or inflammation. The most studied gene associated with UVB-mediated cellular processes is cyclooxygenase 2 *(COX-2* or *PTGS2)*. Its upregulation is described as a diagnostic marker and predictive factor in most tumors and other diseases related to chronic inflammation [68,69]. The role of *COX-2* inhibition is paramount important in prevention and treatment of different types of cancer [70]. *In vivo* studies have demonstrated that the use of specific inhibitors of *COX-2* leads to accumulation of cells in G0/G1 phase of cell cycle and reduced proliferation of cancer cells [69]. Our study confirmed upregulation of *COX-2* in UVB-irradiated keratinocytes, and here we also demonstrated that its increased expression was CPD-dependent (Fig 2, S3E Fig).

Furthermore, we have identified another gene, *CCNE1*, which similarly to *COX-2* related to enhanced cell proliferation and upregulated in a CPD-dependent manner. A study using a mouse model has shown that the cell cycle disturbance in epidermal keratinocytes subjected to an erythematogenic dose of UVB irradiation is at least in part caused by increased expression of cyclins, including cyclin E1 [71]. Their data suggests that UVB-induced hyperplasia and tumorigenesis is partly mediated by the upregulation of cyclin E1 [71], it however has not been investigated in humans. Several studies suggested that *CCNE1* may represent a useful prognostic marker and has the potential to be a target for therapy in breast and ovarian cancer [72,73]. This has not yet been investigated in skin cancer.

The second best scored gene network was related to transcriptional machinery. We validated the microarray results for six transcriptional regulators, *ATF3*[74], *EGR1*[75], *ID2*[76], *RUNX1*[77], *SNAI1*[78] and *SNAI2*[78]. Mutations or altered expression of these genes has been reported in various types of cancer, including skin cancer [74–78]. Upregulation of *ATF3*[41], *EGR1*[41], *ID2*[42], *SNAI1*[43] and *SNAI2*[43] has also been implicated in UV-induced cellular stress responses [41–43]. Here, we have demonstrated that expression of these transcription regulators are CPD-dependent, which could serve as novel biomarkers for evaluation of the involvement of UVB in various photosensitive skin disorders or skin cancers. Our findings underscore that UVB-induced CPD photolesions play a critical role in tumor development, including skin carcinogenesis and not only by induction of mutations but also by changing the expression of physiologically important genes.

The question of how CPD photolesions activate intracellular signalling and how they modulate the expression of genes in cellular mechanisms (e.g. cell cycle arrest) in response to UVB irradiation has not been answered. Using CPD-photolyase expressing transgenic mice, it has been shown previously that unrepaired CPD photolesions are the major mediator of UV-induced transcriptional responses and induce S-phase cell cycle arrest [49]. UV radiation activates different signal transduction pathways in a wavelength and dose-dependent manner [79]. It is well established that these signal pathways are regulated by specific protein kinases including p38 kinase, PKC, AKT and JNK, which play a crucial role in the response network to skin damage caused by UV exposure [46]. We examined how specific inhibitors of protein kinases, such as p38 kinase, AKT and JNK, affect gene expression changes initiated by CPD photolesions. We found that only the JNK inhibitor influenced UVB-induced overexpression of cyclin E1 and p15INK4b proteins mainly at 24 hours after UVB irradiation. Reviewing the literature [80] and comparing the data to our microarray findings (S1 and S2 Tables) forty-two CPD-dependent genes could be identified that might be regulated through the activation of JNK. These findings indicate an important role for JNK kinases in the control of gene expression modulated by CPD photolesions. These genes are involved in cell adhesion, apoptosis, regulation of the cell cycle and transcriptional machineries, cytoskeletal remodelling, cellular development, growth and proliferation. Activation of *NF-κB* and *AP-1* transcription factors (the major downstream targets of the JNK cascade to regulate the expression of several genes involved in proliferation and survival pathways or inflammation) are candidates to mediate the effect of JNK upon UVB irradiation [46]. Increased levels of the activated form of JNK have been shown in UV exposed keratinocytes [81,82]. It has been reported that active JNK is mainly associated with keratinocyte proliferation and differentiation [80,83]. Some studies demonstrated enhanced JNK activity in psoriatic and wound-healing epidermal cells [84,85], as well as in cylindromas and other hair follicle derived tumors [86]. Moreover, experiments using animal or human tissue samples suggested that the JNK-Ap1 signalling pathway has an important role in development of squamous cell carcinoma and melanoma [86]. It is also possible that CPDs (or other UV-induced DNA-lesions) formed in the promoter or enhancer region of genes alter binding potentials of transcription factor leading to change in gene expression [87,88]. These published data raise the question whether expression of certain genes can be changed directly by UV-induced CPDs formed in their promoter region rather than through a damage-response signalling pathway. The answer to this question should come from studies with systematic investigation using specific inhibitors of signalling pathways to prove that indeed the observed transcriptional changes is mediated through a CPD-dependent signalling event or caused by a change in the promoter. Studies are ongoing to discern the mechanisms of CPD photolesion-mediated activation of JNK and its downstream effectors.

## Conclusion

We demonstrated that *in vitro*-transcribed mRNA encoding non-human CPD-photolyase can efficiently be translated into a functional protein in cultured human keratinocytes. Using pseudouridine-modified mRNA encoding CPD-specific photolyase, which can specifically remove UVB-induced CPD lesions after exposure to photoreactivating light, we found that the CPD lesions represents the major contributors to the transcriptional response to UVB irradiation. Network analysis of CPD-regulated genes revealed that CPDs are principal mediators of biological processes related to cell cycle signalling and transcriptional control. Consequently, the presence of CPDs modulated the transcription profile of many genes that are involved in the regulation of cell cycle (e.g. CCNE1, CDKN2B) or function as transcription factors (e.g. ATF3, ID2, RUNX1). Taken together, the results presented here demonstrates that an approach based on transfection of *in vitro*-transcribed mRNA is suitable for distinguishing UVB-induced CPD-dependent and-independent cellular mechanisms, and explore the molecular details of the involved signalling pathways.

*In vitro*-transcribed mRNA can be easily generated and it is a new platform for the delivery of therapeutic proteins opening wide perspectives for dermatological or other medical utilizations. This novel mRNA-based model provides an opportunity to identify additional UV-specific molecular targets and achieve a better understanding of UVB-mediated skin diseases.

## Supporting Information

S1 Fig Divergence of UVB-mediated gene expression changes using CPD-specific photolyase encoded by *in vitro*-transcribed mRNA. To characterize the expression profile of CPD-related genes, oligonucleotide microarray was carried out as described in Materials and Methods. Bar graph represents the total number of UVB-responsive genes determined 6 and 24 h after the exposure. Bioset was divided into CPD-independent (the presence of active photolyase had no effect on the expression level of genes modified by UVB irradiation) and CPD-dependent (the presence of active photolyase has restored the expression level of genes modified by UVB irradiation) genes. Cut-off values for changes in gene expression were set at ± 2-fold.
(PDF)

S2 Fig Top network interactions among CPD-dependent genes determined at 6 and 24 h after UVB irradiation in CPD-PL Ψ-mRNA transfected keratinocytes. To analyze network interactions of CPD-dependent genes, datasets representing differentially regulated genes derived from microarray were imported into the Ingenuity Pathway Analysis (IPA) application. The list of the top three networks and associated cellular functions of all CPD-related gene datasets are shown with their respective scores and *p*-values (p < 0.05) obtained from IPA (panel A). The score is derived from a *p*-value and indicates the likelihood of the focus genes in a network being found together due to random chance (defined as:—log10 (p-value)). The most highly rated networks of genes, determined 6 (panel B) and 24 h (panel C) after UVB irradiation, are illustrated with the significantly up- (red shaded) and downregulated (green shaded) genes modulated in a CPD-dependent manner. Genes in empty nodes were not identified as differentially expressed in our experiment and were generated automatically by IPA Knowledge Base indicating a relevance to this network. The genes marked with blue circle have been validated by RT-qPCR.
(PDF)

S3 Fig Functional classification of CPD-related genes belonging to top rated networks determined by IPA. To analyze functional classification of CPD-responsive genes belonging to top networks, datasets derived from the results of network analysis were imported into the Database for Annotation, Visualization and Integrated Discovery (DAVID) tool. The list of these CPD-dependent genes, determined at 6 and 24 h after UVB irradiation is shown according to their cellular functions (panel A-G). Gene expression values measured in photolyase mRNA transfected and UVB irradiated cells (PL+UVB) were compared to those measured in non-UVB irradiated control cells, while photolyase mRNA transfected, UVB-irradiated and photoreactivated samples (active CPD-photolyase) were compared to those that were photolyase mRNA transfected and UVB irradiated, but left without photoreactivation (inactive CPD-photolyase). Cut-off values for changes in gene expression were set at ± 2-fold. To evaluate statistical analysis unpaired, Student’s *t*-test followed by Benjamini-Hochberg corrections were used. The genes marked with red boxes were selected for further investigation. The blue box in (F) represents a gene (IL6) that was confirmed in previous work [19] to have CPD-dependent changes in expression.
(PDF)

S1 Table List of all up and downregulated genes related to CPD lesions determined in CPD-PL Ψ-mRNA transfected human keratinocytes at 6 h after UVB exposure by microarray analyses. (DOCX)

S2 Table List of all up and downregulated genes related to CPD lesions determined in CPD-PL Ψ -mRNA transfected human keratinocytes at 24 h after UVB exposure by microarray analyses. (DOCX)
