# Supplementary material for: Identification of Cyclobutane Pyrimidine Dimer-Responsive Genes Using UVB-Irradiated Human Keratinocytes Transfected with In Vitro-Synthesized Photolyase mRNA
Source: PLoS One. 2015 Jun 29;10(6):e0131141. doi: 10.1371/journal.pone.0131141 (PMC4488231; doi:10.1371/journal.pone.0131141)
Supplement: S1 Fig — To characterize the expression profile of CPD-related genes, oligonucleotide microarray was carried out as described in Materials and Methods. Bar graph represents the total number of UVB-responsive genes determined 6 and 24 h after the exposure. Bioset was divided into CPD-independent (the presence of active photolyase had no effect on the expression level of genes modified by UVB irradiation) and CPD-dependent (the presence of active photolyase has restored the expression level of genes modified by UVB irradiation) genes. Cut-off values for changes in gene expression were set at ± 2-fold. (PDF) [file pone.0131141.s001.pdf]

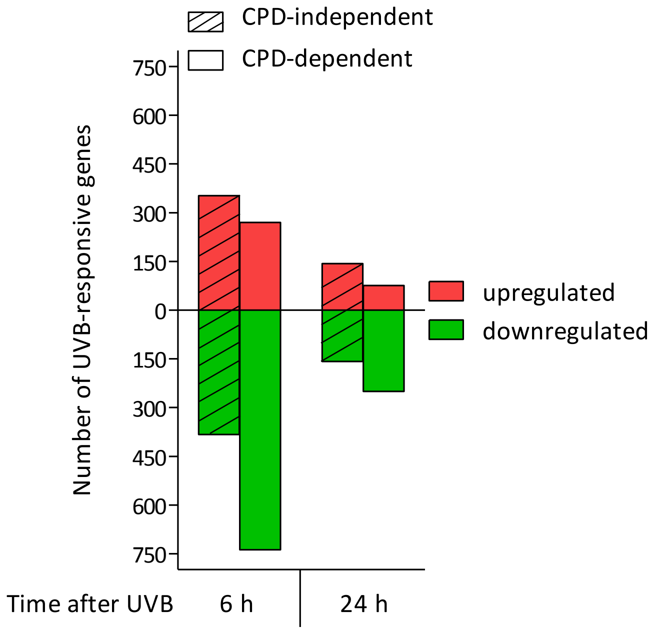
CPD-independent
CPD-dependent
Number of UVB-responsive genes
750
600
450
300
150
0
150
300
450
600
750
upregulated
downregulated
Time after UVB
6 h
24 h
