# Supplementary material for: Identification of Cyclobutane Pyrimidine Dimer-Responsive Genes Using UVB-Irradiated Human Keratinocytes Transfected with In Vitro-Synthesized Photolyase mRNA
Source: PLoS One. 2015 Jun 29;10(6):e0131141. doi: 10.1371/journal.pone.0131141 (PMC4488231; doi:10.1371/journal.pone.0131141)
Supplement: S2 Fig — To analyze network interactions of CPD-dependent genes, datasets representing differentially regulated genes derived from microarray were imported into the Ingenuity Pathway Analysis (IPA) application. The list of the top three networks and associated cellular functions of all CPD-related gene datasets are shown with their respective scores and p-values (p < 0.05) obtained from IPA (panel A). The score is derived from a p-value and indicates the likelihood of the focus genes in a network being found together due to random chance (defined as:—log10 (p-value)). The most highly rated networks of genes, determined 6 (panel B) and 24 h (panel C) after UVB irradiation, are illustrated with the significantly up- (red shaded) and downregulated (green shaded) genes modulated in a CPD-dependent manner. Genes in empty nodes were not identified as differentially expressed in our experiment and were generated automatically by IPA Knowledge Base indicating a relevance to this network. The genes marked with blue circle have been validated by RT-qPCR. (PDF) [file pone.0131141.s002.pdf]

(A)

Top cellular functional categories among the total of 1334 CPD-regulated genes				
**Time after UVB**	**Associated Functions**	**CPD-regulated genes**	***p*-value**	**Score**
6 h	Cell Cycle, Gene Expression, Cancer	122	$2.3 \times 10^{-19} – 4.6 \times 10^{-2}$	130
24 h	Cellular Development, Cellular Growth and Proliferation, Cell Death and Survival	60	$1.4 \times 10^{-5} – 4.9 \times 10^{-2}$	68

(B)

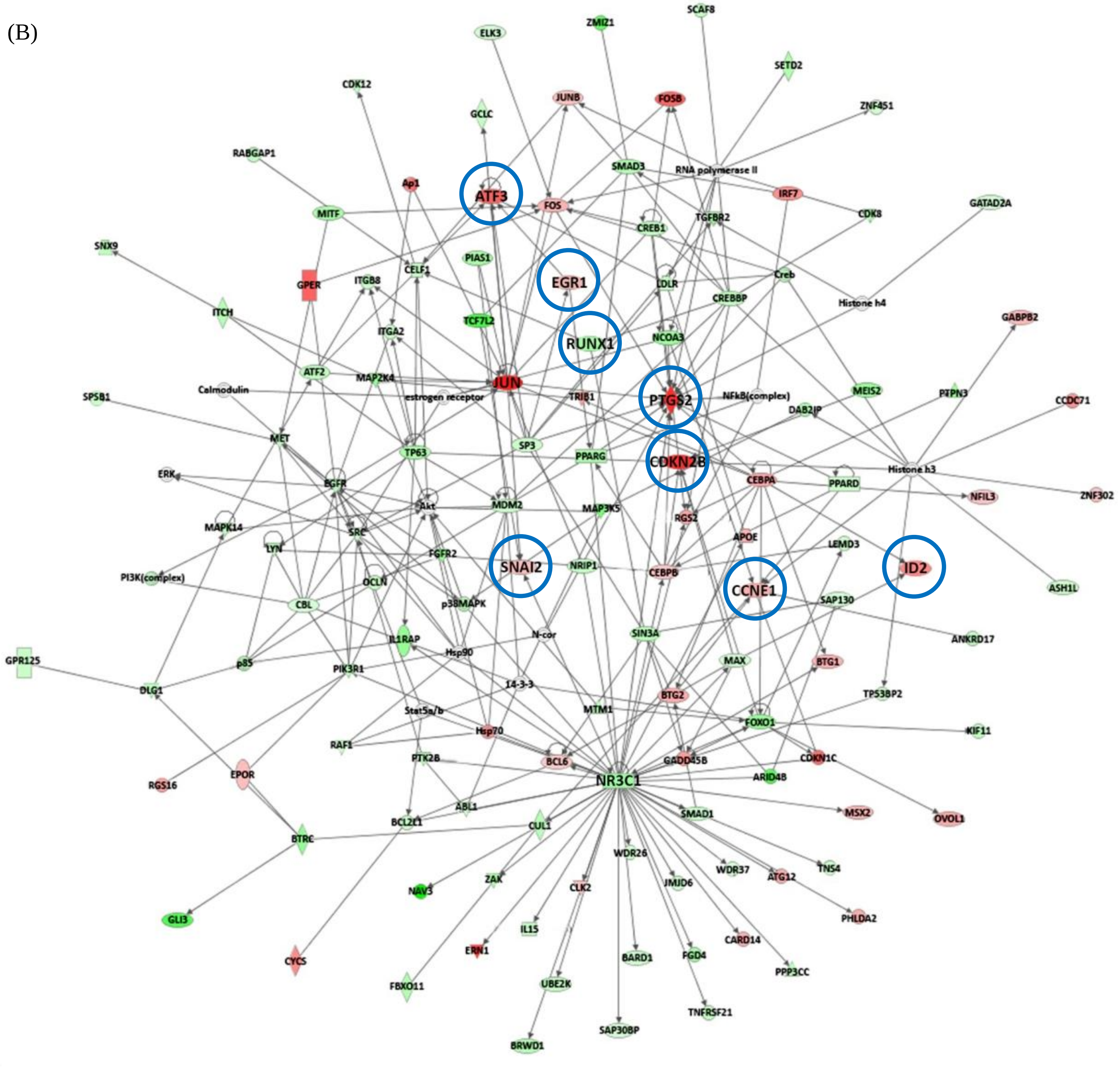

(C)

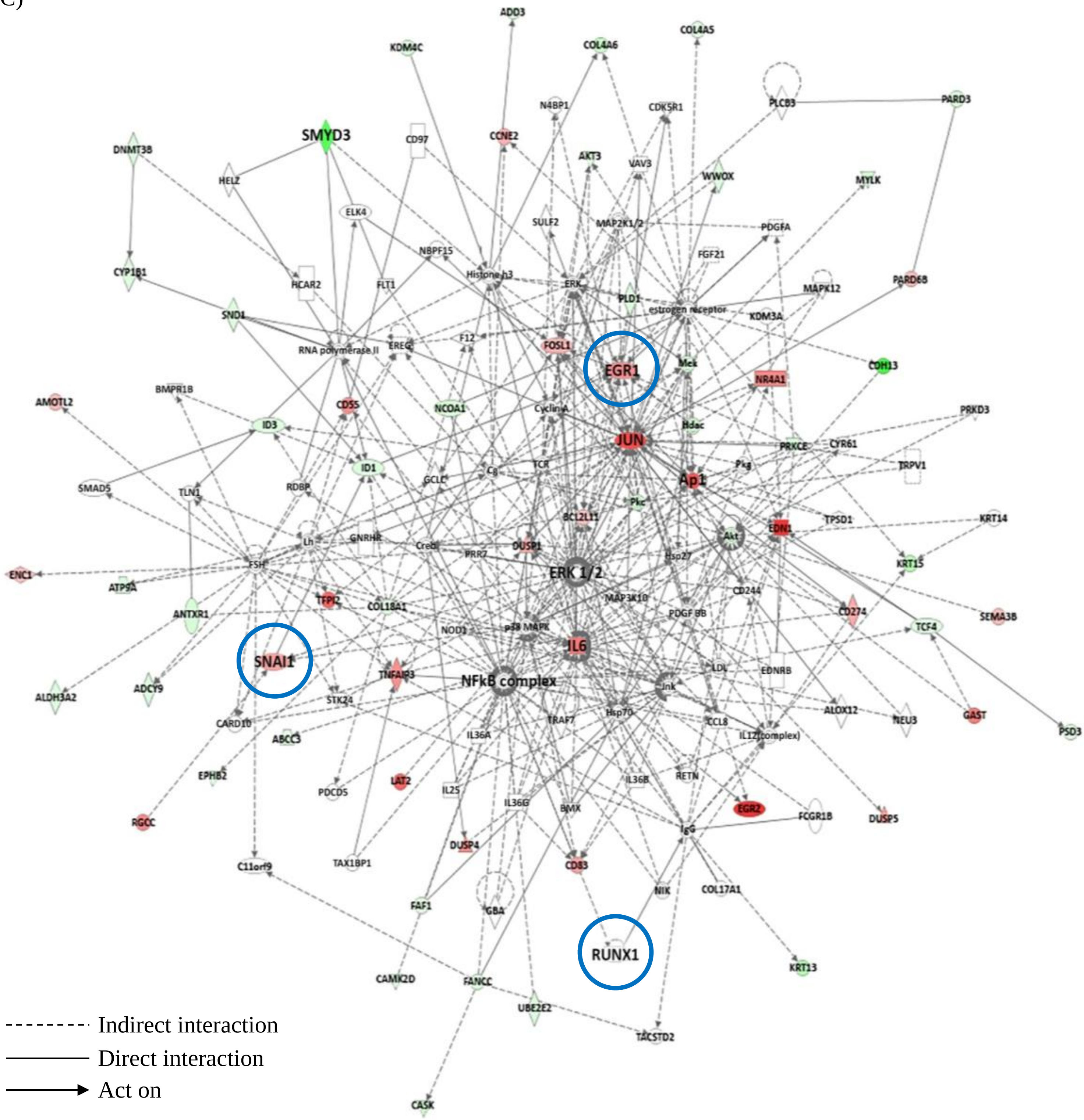

ADD3
COL4A5
KDM4C
COL4A6
N4BP1
CDK5R1
PLCB3
PARD3
SMYD3
DNMT3B
CD97
CCNE2
AKT3
VAV3
WWOX
MYLK
HELZ
ELK4
SULF2
MAP2K1/2
PDGFA
NBPF15
FGF21
CYP1B1
HCAR2
FLT1
Histone h3
ERK
MAPK12
PARD6B
SND1
PLD1
estrogen receptor
KDM3A
EREG
F12
FOSL1
RNA polymerase II
EGR1
Mek
CDH13
NR4A1
AMOTL2
BMPR1B
CD55
NCOA1
Cyclin A
PRKD3
ID3
Hdac
JUN
PRKCE
CYR61
ID1
TCR
Pkg
TRPV1
SMAD5
TLN1
RDBP
GCLC
Ap1
Pkc
BCL2L11
TPSD1
KRT14
EDN1
Lh
GNRHR
Creb
Akt
ENC1
FSH
PRR7
DUSP1
Hsp27
KRT15
ATP9A
ERK 1/2
CD244
MAP3K10
ANTXR1
TFPI2
COL18A1
PDGF BB
CD274
SEMA3B
NOD1
p38 MAPK
TCF4
IL6
SNAI1
TNFAIP3
NFkB complex
LDL
EDNRB
ALDH3A2
ADCY9
STK24
Jnk
CARD10
TRAF7
Hsp70
CCL8
ALOX12
NEU3
GAST
ABCC3
IL36A
IL12 (complex)
PSD3
EPHB2
IL36B
RETN
PDCD5
LAT2
IL25
RGCC
IL36G
BMX
EGR2
FCGR1B
DUSP5
IgG
DUSP4
C11orf9
TAX1BP1
CD83
NIK
COL17A1
FAF1
GBA
RUNX1
KRT13
CAMK2D
FANCC
UBE2E2
TACSTD2
CASK
Indirect interaction
Direct interaction
Act on
