# Supplementary figures and images for: Identification of Cyclobutane Pyrimidine Dimer-Responsive Genes Using UVB-Irradiated Human Keratinocytes Transfected with In Vitro-Synthesized Photolyase mRNA
Source: PLoS One. 2015 Jun 29;10(6):e0131141. doi: 10.1371/journal.pone.0131141 (PMC4488231; doi:10.1371/journal.pone.0131141)

(A)

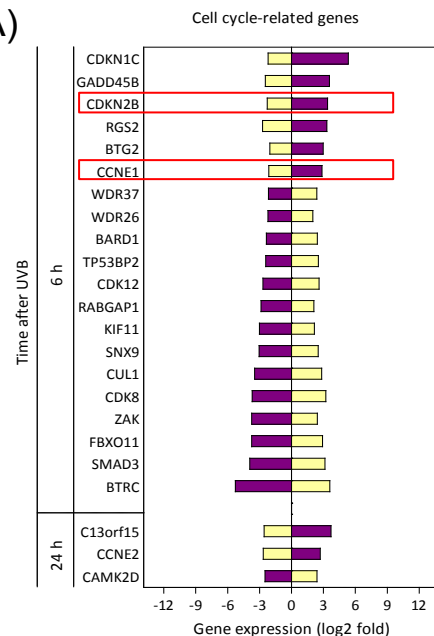

(B)

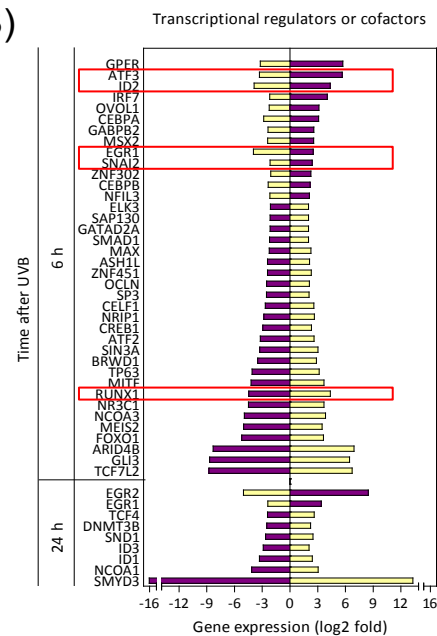

(C)

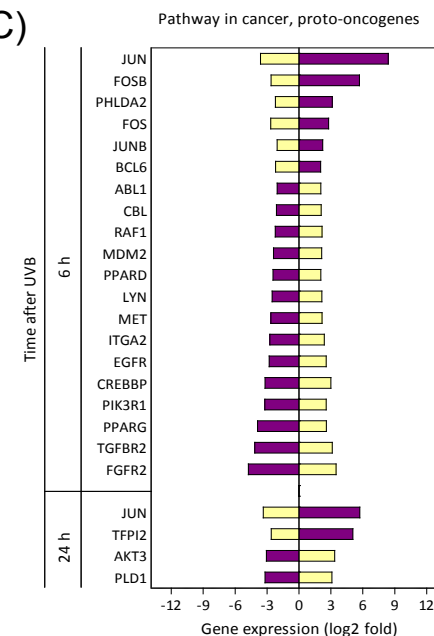

■ PL+UVB / non-irradiated  
■ active PL / inactive PL

(D)

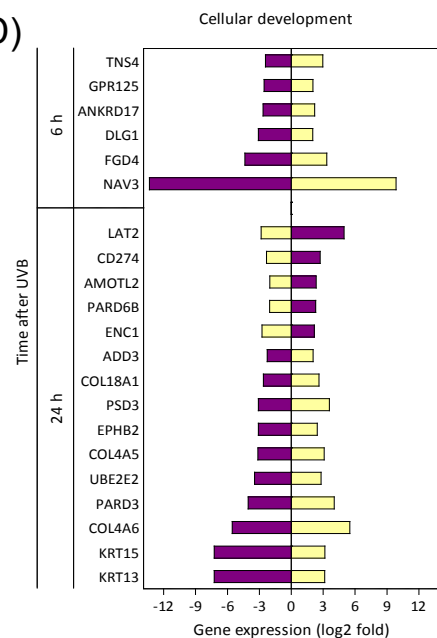

(E)

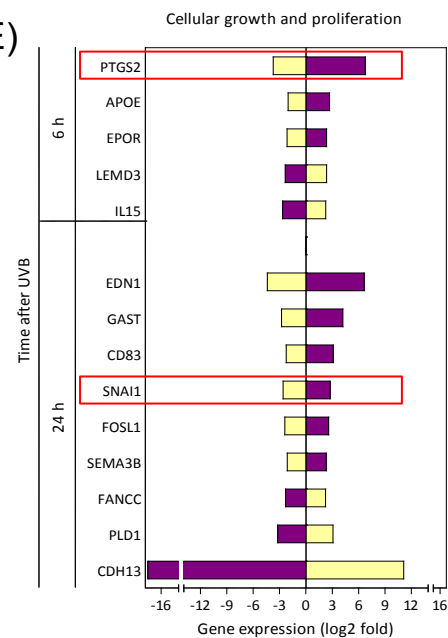

(F)

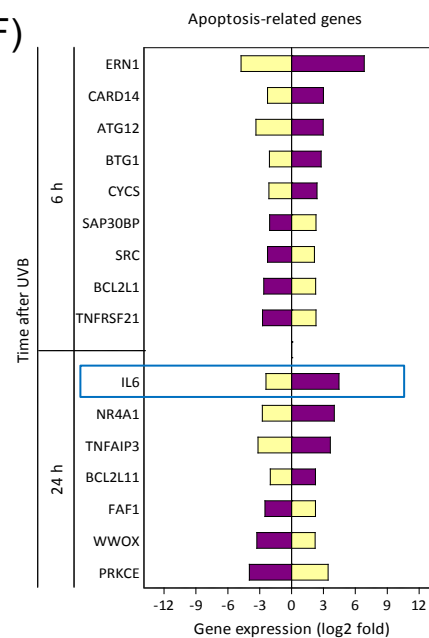

(G)

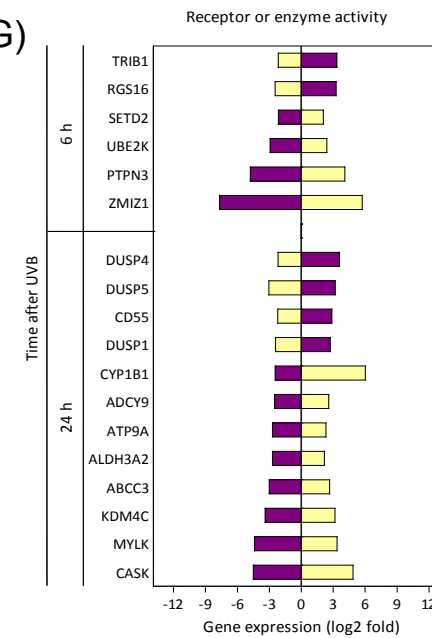

Supplement: S3 Fig — To analyze functional classification of CPD-responsive genes belonging to top networks, datasets derived from the results of network analysis were imported into the Database for Annotation, Visualization and Integrated Discovery (DAVID) tool. The list of these CPD-dependent genes, determined at 6 and 24 h after UVB irradiation is shown according to their cellular functions (panel A-G). Gene expression values measured in photolyase mRNA transfected and UVB irradiated cells (PL+UVB) were compared to those measured in non-UVB irradiated control cells, while photolyase mRNA transfected, UVB-irradiated and photoreactivated samples (active CPD-photolyase) were compared to those that were photolyase mRNA transfected and UVB irradiated, but left without photoreactivation (inactive CPD-photolyase). Cut-off values for changes in gene expression were set at ± 2-fold. To evaluate statistical analysis unpaired, Student’s t-test followed by Benjamini-Hochberg corrections were used. The genes marked with red boxes were selected for further investigation. The blue box in (F) represents a gene (IL6) that was confirmed in previous work [19] to have CPD-dependent changes in expression. (PDF) [file pone.0131141.s003.pdf]
