# Supplementary material for: Identification of Cyclobutane Pyrimidine Dimer-Responsive Genes Using UVB-Irradiated Human Keratinocytes Transfected with In Vitro-Synthesized Photolyase mRNA
Source: PLoS One. 2015 Jun 29;10(6):e0131141. doi: 10.1371/journal.pone.0131141 (PMC4488231; doi:10.1371/journal.pone.0131141)
Supplement: S1 Table — (DOCX) [file pone.0131141.s004.docx]

| **S1 Table. List of 270 upregulated CPD-dependent genes 6 h after UVB exposure.** | | | | | | | | | | | | | | | | | | | | | | | | | | | | | | | | | | | | |
| --- | --- | --- | --- | --- | --- | --- | --- | --- | --- | --- | --- | --- | --- | --- | --- | --- | --- | --- | --- | --- | --- | --- | --- | --- | --- | --- | --- | --- | --- | --- | --- | --- | --- | --- | --- | --- |
| **Accession Number** | | | | | **Gene symbol** | | | **UVB vs. non-irradiated fold change** | | | | | | | | | | | | | | | | | | | ***p* value** | **Active photolyase vs. inactive photolyase fold change** 🟋 | | | | | |  | ***p* value** |  |
| NM_001124 | | | | | ADM (J) | | | 3.01 | | | | | | | | | | | | | | | | | | | 8.06E-04 | -2.18 | | | | | |  | 4.20E-03 |  |
| NM_032804 | | | | | ADO | | | 3.30 | | | | | | | | | | | | | | | | | | | 9.95E-04 | -2.51 | | | | | |  | 1.53E-02 |  |
| NM_005165 | | | | | ALDOC | | | 2.12 | | | | | | | | | | | | | | | | | | | 1.76E-02 | -2.17 | | | | | |  | 2.47E-02 |  |
| NM_023039 | | | | | ANKRA2 | | | 2.80 | | | | | | | | | | | | | | | | | | | 5.97E-04 | -2.57 | | | | | |  | 4.95E-03 |  |
| NM_181726 | | | | | ANKRD37 | | | 3.35 | | | | | | | | | | | | | | | | | | | 1.20E-03 | -3.67 | | | | | |  | 3.81E-03 |  |
| NM_023016 | | | | | ANKRD57 | | | 2.98 | | | | | | | | | | | | | | | | | | | 5.10E-04 | -2.24 | | | | | |  | 2.51E-03 |  |
| NM_003734 | | | | | AOC3 | | | 12.26 | | | | | | | | | | | | | | | | | | | 4.03E-04 | -6.16 | | | | | |  | 3.23E-03 |  |
| NM_000041 | | | | | APOE | | | 2.69 | | | | | | | | | | | | | | | | | | | 1.28E-02 | -2.03 | | | | | |  | 3.01E-02 |  |
| NM_006673 | | | | | ARID5A | | | 2.50 | | | | | | | | | | | | | | | | | | | 2.57E-04 | -2.07 | | | | | |  | 2.88E-03 |  |
| NM_005738 | | | | | ARL4A | | | 2.64 | | | | | | | | | | | | | | | | | | | 6.52E-03 | -2.21 | | | | | |  | 2.29E-02 |  |
| NM_183376 | | | | | ARRDC4 | | | 4.30 | | | | | | | | | | | | | | | | | | | 5.15E-03 | -3.07 | | | | | |  | 1.28E-02 |  |
| NM_001040619 | | | | | ATF3 🟋 (J) | | | 5.65 | | | | | | | | | | | | | | | | | | | 2.65E-03 | -3.30 | | | | | |  | 6.36E-03 |  |
| AB002449 | | | | | ATG12 | | | 2.98 | | | | | | | | | | | | | | | | | | | 7.85E-03 | -3.35 | | | | | |  | 3.79E-03 |  |
| NM_033388 | | | | | ATG16L2 | | | 2.24 | | | | | | | | | | | | | | | | | | | 4.36E-03 | -2.05 | | | | | |  | 4.34E-03 |  |
| NM_080653 | | | | | ATP6V1E2 | | | 2.29 | | | | | | | | | | | | | | | | | | | 4.64E-02 | -2.20 | | | | | |  | ns. |  |
| NM_080605 | | | | | B3GALT6 | | | 3.38 | | | | | | | | | | | | | | | | | | | 7.10E-04 | -2.28 | | | | | |  | 4.43E-03 |  |
| NM_012342 | | | | | BAMBI | | | 3.30 | | | | | | | | | | | | | | | | | | | 3.25E-04 | -2.19 | | | | | |  | 7.73E-03 |  |
| NM_138931 | | | | | BCL6 (J) | | | 2.03 | | | | | | | | | | | | | | | | | | | 1.68E-03 | -2.20 | | | | | |  | 1.49E-02 |  |
| NM_153274 | | | | | BEST4 | | | 2.04 | | | | | | | | | | | | | | | | | | | 3.01E-02 | -2.03 | | | | | |  | ns. |  |
| NM_001731 | | | | | BTG1 | | | 2.79 | | | | | | | | | | | | | | | | | | | 6.75E-03 | -2.09 | | | | | |  | 3.43E-03 |  |
| NM_006763 | | | | | BTG2 (J) | | | 2.98 | | | | | | | | | | | | | | | | | | | 5.02E-04 | -2.03 | | | | | |  | 7.93E-03 |  |
| AB209777 | | | | | BTN2A1 | | | 6.37 | | | | | | | | | | | | | | | | | | | 1.05E-03 | -4.43 | | | | | |  | 3.47E-03 |  |
| BC050694 | | | | | C10orf110 | | | 2.87 | | | | | | | | | | | | | | | | | | | 2.68E-03 | -2.27 | | | | | |  | 6.17E-03 |  |
| NM_031450 | | | | | C11orf68 | | | 4.56 | | | | | | | | | | | | | | | | | | | 5.80E-04 | -2.67 | | | | | |  | 1.80E-02 |  |
| BC007973 | | | | | C12orf47 | | | 5.38 | | | | | | | | | | | | | | | | | | | 7.53E-04 | -3.64 | | | | | |  | 3.54E-03 |  |
| NM_207435 | | | | | C12orf76 | | | 4.50 | | | | | | | | | | | | | | | | | | | 1.78E-03 | -3.66 | | | | | |  | 4.56E-03 |  |
| NM_174891 | | | | | C14orf79 | | | 3.80 | | | | | | | | | | | | | | | | | | | 1.20E-03 | -2.23 | | | | | |  | 1.44E-02 |  |
| NM_182563 | | | | | C16orf79 | | | 2.92 | | | | | | | | | | | | | | | | | | | 5.46E-04 | -2.62 | | | | | |  | 3.54E-03 |  |
| NM_001014979 | | | | | C16orf93 | | | 3.94 | | | | | | | | | | | | | | | | | | | 5.97E-04 | -2.68 | | | | | |  | 5.87E-03 |  |
| NM_020233 | | | | | C17orf48 | | | 2.57 | | | | | | | | | | | | | | | | | | | 2.30E-03 | -2.21 | | | | | |  | 7.99E-03 |  |
| NM_001039842 | | | | C17orf90 | | | | | | | | | | | | | | | | | | 2.85 | | 9.04E-03 | | | | | | -2.21 | | 1.63E-02 | | | |  |
| NM_032895 | | | | C17orf91 | | | | | | | | | | | | | | | | | | 4.62 | | 4.03E-04 | | | | | | -2.71 | | 3.57E-03 | | | |  |
| NM_001012716 | | | | C18orf56 | | | | | | | | | | | | | | | | | | 3.12 | | 6.49E-03 | | | | | | -2.54 | | 2.50E-02 | | | |  |
| NM_014388 | | | | C1orf107 | | | | | | | | | | | | | | | | | | 2.92 | | 1.94E-03 | | | | | | -2.43 | | 7.99E-03 | | | |  |
| NM_174896 | | | | C1orf162 | | | | | | | | | | | | | | | | | | 9.03 | | 3.90E-04 | | | | | | -5.37 | | 3.11E-03 | | | |  |
| NM_001012971 | | | | C20orf106 | | | | | | | | | | | | | | | | | | 3.61 | | 6.43E-04 | | | | | | -2.80 | | 4.91E-03 | | | |  |
| NM_173793 | | | | C22orf39 | | | | | | | | | | | | | | | | | | 2.64 | | 6.13E-04 | | | | | | -2.06 | | 4.98E-03 | | | |  |
| NM_001170330 | | | | C4orf3 | | | | | | | | | | | | | | | | | | 2.29 | | 5.81E-03 | | | | | | 2.37 | | 6.94E-03 | | | |  |
| NM_001008739 | | | | C6orf226 | | | | | | | | | | | | | | | | | | 3.39 | | 3.60E-03 | | | | | | -2.15 | | 2.15E-02 | | | |  |
| AK092392 | | | | C6orf25 | | | | | | | | | | | | | | | | | | 4.50 | | 1.18E-02 | | | | | | -2.35 | | ns. | | | |  |
| NM_145267 | | | | C6orf57 | | | | | | | | | | | | | | | | | | 2.70 | | 3.49E-03 | | | | | | -2.77 | | 3.54E-03 | | | |  |
| NM_145111 | | | | C7orf38 | | | | | | | | | | | | | | | | | | 2.77 | | 1.24E-03 | | | | | | -2.20 | | 5.59E-03 | | | |  |
| NM_182597 | | | | C7orf53 | | | | | | | | | | | | | | | | | | 3.72 | | 3.50E-04 | | | | | | -2.92 | | 3.43E-03 | | | |  |
| NM_197964 | | | | C7orf55 | | | | | | | | | | | | | | | | | | 2.09 | | 1.54E-02 | | | | | | -2.00 | | 1.92E-02 | | | |  |
| NM_000606 | | | | C8G | | | | | | | | | | | | | | | | | | 4.18 | | 1.18E-03 | | | | | | -2.60 | | 9.06E-03 | | | |  |
| NM_019607 | | | | C8orf44 | | | | | | | | | | | | | | | | | | 2.32 | | 1.17E-02 | | | | | | -2.06 | | 2.24E-02 | | | |  |
| NM_033428 | | | | C9orf123 | | | | | | | | | | | | | | | | | | 5.28 | | 4.60E-04 | | | | | | -3.77 | | 4.25E-03 | | | |  |
| NM_032937 | | | | C9orf37 | | | | | | | | | | | | | | | | | | 3.35 | | 1.05E-03 | | | | | | -2.23 | | 9.04E-03 | | | |  |
| NM_017998 | | | | C9orf40 | | | | | | | | | | | | | | | | | | 3.21 | | 1.48E-02 | | | | | | -2.67 | | 3.36E-02 | | | |  |
| NM_018956 | | | | C9orf9 | | | | | | | | | | | | | | | | | | 2.49 | | 4.25E-02 | | | | | | -2.11 | | ns. | | | |  |
| NM_021189 | | | | CADM3 | | | | | | | | | | | | | | | | | | 2.05 | | 1.33E-02 | | | | | | -2.09 | | 3.08E-02 | | | |  |
| NM_024110 | | | | CARD14 | | | | | | | | | | | | | | | | | | 3.00 | | 6.46E-03 | | | | | | -2.26 | | 4.99E-03 | | | |  |
| AK023691 | | | | CCDC71 | | | | | | | | | | | | | | | | | | 4.02 | | 3.68E-04 | | | | | | -3.04 | | 4.83E-03 | | | |  |
| AK098422 | | | | CCDC71L | | | | | | | | | | | | | | | | | | 2.88 | | 1.23E-03 | | | | | | -2.38 | | 1.17E-02 | | | |  |
| NM_207310 | | | | CCDC74B | | | | | | | | | | | | | | | | | | 2.51 | | 8.52E-03 | | | | | | -2.37 | | 3.35E-02 | | | |  |
| NM_001238 | | | | CCNE1 🟋 | | | | | | | | | | | | | | | | | | 2.87 | | 5.46E-04 | | | | | | -2.14 | | 7.93E-03 | | | |  |
| NM_000076 | | | | CDKN1C (J) | | | | | | | | | | | | | | | | | | 5.34 | | 7.50E-04 | | | | | | -2.20 | | ns. | | | |  |
| BC018086 | | | | CDKN2AIPNL | | | | | | | | | | | | | | | | | | 3.10 | | 4.43E-04 | | | | | | -2.52 | | 3.57E-03 | | | |  |
| NM_078487 | | | | CDKN2B | | | | | | | | | | | | | | | | | | 7.76 | | 5.08E-04 | | | | | | -3.36 | | 1.50E-02 | | | |  |
| NM_004364 | | | | CEBPA 🟋 | | | | | | | | | | | | | | | | | | 3.11 | | 5.97E-04 | | | | | | -2.84 | | 2.60E-03 | | | |  |
| NM_005194 | | | | CEBPB 🟋 | | | | | | | | | | | | | | | | | | 2.18 | | 1.60E-03 | | | | | | -2.36 | | 1.04E-02 | | | |  |
| NM_001928 | | | | CFD | | | | | | | | | | | | | | | | | | 2.53 | | 1.02E-03 | | | | | | -2.16 | | 8.14E-03 | | | |  |
| NM_001008390 | | | | CGGBP1 (J) | | | | | | | | | | | | | | | | | | 2.20 | | 1.62E-03 | | | | | | -2.06 | | 5.81E-03 | | | |  |
| NM_001011667 | | | | CHCHD7 | | | | | | | | | | | | | | | | | | 3.20 | | 2.62E-03 | | | | | | -2.70 | | 8.64E-03 | | | |  |
| AK128423 | | | | | | CIRBP | | | | | | | | | | | | | 3.43 | | | | | 5.57E-04 | | | | | | -3.39 | | 7.53E-03 | | | |  |
| NM_006079 | | | | | | CITED2 | | | | | | | | | | | | | 2.88 | | | | | 5.76E-03 | | | | | | -2.61 | | 3.89E-03 | | | |  |
| NM_003993 | | | | | | CLK2 | | | | | | | | | | | | | 2.23 | | | | | 5.79E-03 | | | | | | -2.01 | | 1.65E-02 | | | |  |
| NM_006493 | | | | | | CLN5 | | | | | | | | | | | | | 2.66 | | | | | 1.93E-02 | | | | | | -2.29 | | 4.18E-02 | | | |  |
| AL832450 | | | | | | CMTM7 | | | | | | | | | | | | | 2.76 | | | | | 8.96E-03 | | | | | | -2.05 | | 5.09E-03 | | | |  |
| NM_144576 | | | | | | COQ10A | | | | | | | | | | | | | 3.60 | | | | | 1.14E-03 | | | | | | -2.26 | | 7.57E-03 | | | |  |
| NM_001031617 | | | | | | COX19 | | | | | | | | | | | | | 6.57 | | | | | 3.61E-04 | | | | | | -4.41 | | 2.58E-03 | | | |  |
| NM_005209 | | | | | | CRYBA2 | | | | | | | | | | | | | 3.01 | | | | | 2.37E-02 | | | | | | -5.14 | | ns. | | | |  |
| NM_017541 | | | | | | CRYGS | | | | | | | | | | | | | 4.58 | | | | | 4.16E-04 | | | | | | -4.05 | | 3.79E-03 | | | |  |
| NM_000396 | | | | | | CTSK | | | | | | | | | | | | | 2.24 | | | | | 2.50E-03 | | | | | | -2.17 | | 4.98E-03 | | | |  |
| NM_003478 | | | | | | CUL5 | | | | | | | | | | | | | 3.43 | | | | | 2.33E-03 | | | | | | -2.30 | | 1.51E-02 | | | |  |
| NM_018947 | | | | | | CYCS | | | | | | | | | | | | | 2.39 | | | | | 6.66E-03 | | | | | | -2.14 | | 1.15E-02 | | | |  |
| NM_022720 | | | | | | DGCR8 | | | | | | | | | | | | | 2.89 | | | | | 6.20E-03 | | | | | | -2.04 | | 1.44E-02 | | | |  |
| NM_014475 | | | | | | DHDH | | | | | | | | | | | | | 5.20 | | | | | 6.20E-04 | | | | | | -2.93 | | 3.43E-03 | | | |  |
| NM_024308 | | | | | | DHRS11 | | | | | | | | | | | | | 2.63 | | | | | 3.25E-04 | | | | | | -2.13 | | 8.66E-03 | | | |  |
| NM_014953 | | | | | | DIS3 | | | | | | | | | | | | | 2.79 | | | | | 1.50E-03 | | | | | | -2.34 | | 1.04E-02 | | | |  |
| NM_004405 | | | | | | DLX2 (J) | | | | | | | | | | | | | 3.93 | | | | | 5.78E-04 | | | | | | -3.30 | | 4.46E-03 | | | |  |
| NM_012328 | | | | | | DNAJB9 | | | | | | | | | | | | | 2.60 | | | | | 6.89E-04 | | | | | | -2.40 | | 2.80E-03 | | | |  |
| AF339771 | | | | | | DOCK9-AS2 | | | | | | | | | | | | | 5.18 | | | | | 5.64E-04 | | | | | | -4.26 | | 4.01E-03 | | | |  |
| NM_001955 | | | | | | EDN1 | | | | | | | | | | | | | 4.50 | | | | | 1.09E-02 | | | | | | -2.22 | | ns. | | | |  |
| NM_001964 | | | | | | EGR1 | | | | | | | | | | | | | 2.51 | | | | | 1.11E-02 | | | | | | -3.95 | | 1.89E-02 | | | |  |
| NM_001965 | | | | | | EGR4 | | | | | | | | | | | | | 2.99 | | | | | 1.39E-03 | | | | | | -2.05 | | 1.67E-02 | | | |  |
| NM_018696 | | | | | | ELAC1 | | | | | | | | | | | | | 2.88 | | | | | 2.26E-03 | | | | | | -2.69 | | 1.03E-02 | | | |  |
| NM_025165 | | | | | | ELL3 | | | | | | | | | | | | | 2.83 | | | | | 7.71E-04 | | | | | | -2.35 | | 4.07E-03 | | | |  |
| AF229804 | | | | | | ELP2P | | | | | | | | | | | | | 4.98 | | | | | 6.33E-04 | | | | | | -2.44 | | 8.57E-03 | | | |  |
| NM_000121 | | | | | | EPOR | | | | | | | | | | | | | 2.36 | | | | | 4.20E-03 | | | | | | -2.13 | | 3.17E-03 | | | |  |
| NM_152461 | | | | | | ERN1 | | | | | | | | | | | | | 6.84 | | | | | 2.67E-04 | | | | | | -4.76 | | 2.57E-03 | | | |  |
| AK093641 | | | | | | FAM120AOS | | | | | | | | | | | | | 3.90 | | | | | 1.27E-03 | | | | | | -3.14 | | 2.80E-03 | | | |  |
| ENST00000357303 | | | | | | FAM132B | | | | | | | | | | | | | 4.13 | | | | | 8.41E-04 | | | | | | -3.36 | | 7.00E-03 | | | |  |
| AK000005 | | | | | | FAM219B | | | | | | | | | | | | | 3.71 | | | | | 3.54E-03 | | | | | | -2.92 | | 5.96E-03 | | | |  |
| BC043603 | | | | | | FAM44A | | | | | | | | | | | | | 8.39 | | | | | 3.02E-04 | | | | | | -3.84 | | 3.36E-03 | | | |  |
| NM_017633 | | | | | | FAM46A | | | | | | | | | | | | | 3.94 | | | | | 2.19E-03 | | | | | | -2.66 | | 1.48E-02 | | | |  |
| NM_145175 | | | | | | FAM84A | | | | | | | | | | | | | 2.47 | | | | | 2.60E-03 | | | | | | -2.61 | | 4.21E-03 | | | |  |
| NM_201400 | | | | | | FAM86A | | | | | | | | | | | | | 2.87 | | | | | 4.79E-04 | | | | | | -3.90 | | ns. | | | |  |
| NM_032916 | | | | | | FAM86B2 | | | | | | | | | | | | | 2.08 | | | | | 6.85E-04 | | | | | | -2.07 | | ns. | | | |  |
| ENST00000382591 | | | | | | FAM90A7 | | | | | | | | | | | 8.78 | | | | | | | 4.03E-04 | | | | | | -4.57 | | 3.45E-03 | | | |  |
| NM_032301 | | | | | | FBXW9 | | | | | | | | | | | 2.57 | | | | | | | 1.51E-03 | | | | | | -2.01 | | 1.71E-02 | | | |  |
| NM_152429 | | | | | | FGFBP3 | | | | | | | | | | | 2.35 | | | | | | | 6.91E-03 | | | | | | -2.09 | | 3.27E-02 | | | |  |
| AK094772 | | | | | | FLJ37453 | | | | | | | | | | | 2.29 | | | | | | | 6.37E-04 | | | | | | -2.01 | | 3.43E-03 | | | |  |
| NM_005252 | | | | | | FOS 🟋 (J) | | | | | | | | | | | 2.79 | | | | | | | 1.39E-02 | | | | | | -2.66 | | ns. | | | |  |
| NM_006732 | | | | | | FOSB | | | | | | | | | | | 5.69 | | | | | | | 1.26E-03 | | | | | | -2.62 | | 2.38E-02 | | | |  |
| NM_001453 | | | | | | FOXC1 | | | | | | | | | | | 2.95 | | | | | | | 4.06E-04 | | | | | | -2.24 | | 2.94E-03 | | | |  |
| AY007211 | | | | | | FPGS | | | | | | | | | | | 2.94 | | | | | | | 1.16E-03 | | | | | | -2.52 | | 3.45E-03 | | | |  |
| NM_005860 | | | | | | FSTL3 | | | | | | | | | | | 3.40 | | | | | | | 4.50E-04 | | | | | | -2.13 | | 3.55E-03 | | | |  |
| NM_003507 | | | | | | FZD7 | | | | | | | | | | | 3.98 | | | | | | | 3.25E-04 | | | | | | -3.44 | | 2.57E-03 | | | |  |
| NM_144618 | | | | | | GABPB2 | | | | | | | | | | | 2.59 | | | | | | | 7.70E-03 | | | | | | -2.38 | | 1.69E-02 | | | |  |
| NM_021990 | | | | | | GABRE | | | | | | | | | | | 2.35 | | | | | | | 5.48E-03 | | | | | | -2.54 | | 9.27E-03 | | | |  |
| NM_015675 | | | | | | GADD45B 🟋 | | | | | | | | | | | 3.56 | | | | | | | 5.12E-04 | | | | | | -2.46 | | 8.06E-03 | | | |  |
| NM_024523 | | | | | | GCC1 | | | | | | | | | | | 2.27 | | | | | | | 8.18E-04 | | | | | | -2.07 | | 2.80E-03 | | | |  |
| NM_203448 | | | | | | GLIDR | | | | | | | | | | | 3.90 | | | | | | | 5.42E-04 | | | | | | -2.66 | | 2.44E-02 | | | |  |
| AK095071 | | | | | | GNL1 | | | | | | | | | | | 2.34 | | | | | | | 7.85E-03 | | | | | | -2.49 | | 5.59E-03 | | | |  |
| NM_000825 | | | | | | GNRH1 | | | | | | | | | | | 2.48 | | | | | | | 4.76E-04 | | | | | | -2.47 | | 2.57E-03 | | | |  |
| NM_001039966 | | | | | | GPER | | | | | | | | | | | 5.72 | | | | | | | 8.78E-04 | | | | | | -3.21 | | 1.92E-02 | | | |  |
| NM_003272 | | | | | | GPR137B | | | | | | | | | | | 4.55 | | | | | | | 2.76E-02 | | | | | | -2.01 | | 2.57E-03 | | | |  |
| NM_002098 | | | | | | GUCA1B | | | | | | | | | | | 4.90 | | | | | | | 5.25E-04 | | | | | | -3.52 | | 3.57E-03 | | | |  |
| NM_022482 | | | | | | GZF1 | | | | | | | | | | | 3.61 | | | | | | | 7.50E-04 | | | | | | -2.98 | | 3.79E-03 | | | |  |
| L06175 | | | | | | HCP5 | | | | | | | | | | | 2.22 | | | | | | | 3.03E-03 | | | | | | -2.20 | | 2.08E-02 | | | |  |
| NM_006460 | | | | | | HEXIM1 | | | | | | | | | | | 6.17 | | | | | | | 3.25E-04 | | | | | | -3.92 | | 3.57E-03 | | | |  |
| NM_002729 | | | | | | HHEX | | | | | | | | | | | 3.56 | | | | | | | 3.47E-03 | | | | | | -2.40 | | 7.86E-03 | | | |  |
| ENST00000369158 | | | | | | HIST2H3C | | | | | | | | | | | 3.94 | | | | | | | 3.71E-03 | | | | | | -2.96 | | 3.81E-02 | | | |  |
| NM_020834 | | | | | | HOMEZ | | | | | | | | | | | 2.53 | | | | | | | 7.16E-03 | | | | | | -2.68 | | 3.43E-03 | | | |  |
| NR_002795 | | | | | | HOXA11AS | | | | | | | | | | | 4.10 | | | | | | | 2.19E-02 | | | | | | -3.32 | | 4.23E-02 | | | |  |
| NM_006897 | | | | | | HOXC9 | | | | | | | | | | | 2.78 | | | | | | | 4.01E-04 | | | | | | -2.11 | | 3.43E-03 | | | |  |
| NM_021979 | | | | | | HSPA2 | | | | | | | | | | | 3.71 | | | | | | | 3.68E-04 | | | | | | -2.64 | | 3.11E-03 | | | |  |
| NM_007312 | | | | | | HYAL1 | | | | | | | | | | | 2.78 | | | | | | | 5.43E-03 | | | | | | -2.20 | | 2.38E-02 | | | |  |
| NM_002166 | | | | | | ID2 (J) | | | | | | | | | | | 4.35 | | | | | | | 1.83E-03 | | | | | | -3.89 | | 2.57E-03 | | | |  |
| NM_004907 | | | | | | IER2 | | | | | | | | | | | 3.14 | | | | | | | 3.25E-04 | | | | | | -2.29 | | 4.09E-03 | | | |  |
| NM_203434 | | | | | | IER5L | | | | | | | | | | | | | | | 2.72 | | | 2.33E-03 | | | | | | -2.56 | | 4.26E-03 | | | |  |
| NM_001039670 | | | | | | IFFO1 | | | | | | | | | | | | | | | 5.80 | | | 3.51E-04 | | | | | | -3.95 | | 3.11E-03 | | | |  |
| NM_004512 | | | | | | IL11RA | | | | | | | | | | | | | | | 4.34 | | | 2.69E-03 | | | | | | -3.59 | | 1.11E-02 | | | |  |
| NM_182972 | | | | | | IRF2BP2 | | | | | | | | | | | | | | | 2.51 | | | 3.08E-03 | | | | | | -2.00 | | 1.10E-02 | | | |  |
| NM_004031 | | | | | | IRF7 | | | | | | | | | | | | | | | 4.03 | | | 2.81E-02 | | | | | | -2.20 | | ns. | | | |  |
| BC007866 | | | | | | JMJD1C-AS1 | | | | | | | | | | | | | | | 3.91 | | | 9.92E-04 | | | | | | -2.69 | | 5.62E-03 | | | |  |
| NM_002228 | | | | | | JUN 🟋 (J) | | | | | | | | | | | | | | | 8.39 | | | 5.47E-04 | | | | | | -3.62 | | 1.24E-02 | | | |  |
| NM_002229 | | | | | | JUNB | | | | | | | | | | | | | | | 2.23 | | | 2.59E-03 | | | | | | -2.06 | | 5.15E-03 | | | |  |
| BC020847 | | | | | | KANSL1-AS1 | | | | | | | | | | | | | | | 4.39 | | | 1.61E-03 | | | | | | -3.10 | | 4.25E-03 | | | |  |
| NM_170720 | | | | | | KCNJ14 | | | | | | | | | | | | | | | 2.91 | | | 1.39E-03 | | | | | | -2.14 | | 3.59E-03 | | | |  |
| THC2664215 | | | | | | KCNQ1OT1 | | | | | | | | | | | | | | | 3.50 | | | 1.54E-02 | | | | | | -2.28 | | 3.54E-03 | | | |  |
| NM_014949 | | | | | | KIAA0907 | | | | | | | | | | | | | | | 3.09 | | | 6.44E-04 | | | | | | -2.30 | | 6.50E-03 | | | |  |
| NM_032529 | | | | | | KIAA1875 | | | | | | | | | | | | | | | 2.74 | | | 2.58E-03 | | | | | | -2.25 | | 1.81E-02 | | | |  |
| NM_004235 | | | | | | KLF4 (J) | | | | | | | | | | | | | | | 3.83 | | | 3.61E-04 | | | | | | -2.21 | | 3.45E-03 | | | |  |
| NM_052971 | | | | | | LEAP2 | | | | | | | | | | | | | | | 3.96 | | | 4.94E-04 | | | | | | -3.09 | | 3.54E-03 | | | |  |
| BC036622 | | | | | | LINC01003 | | | | | | | | | | | | | | | 7.19 | | | 5.03E-04 | | | | | | -4.74 | | 4.29E-03 | | | |  |
| NR_002809 | | | | | | LINC01089 | | | | | | | | | | | | | | | 4.50 | | | 1.17E-03 | | | | | | -3.53 | | 3.43E-03 | | | |  |
| AK127450 | | | | | | LOC439911 | | | | | | | | | | | | | | | 4.38 | | | 1.65E-03 | | | | | | -2.73 | | 1.30E-02 | | | |  |
| BC030123 | | | | | | LOC441461 | | | | | | | | | | | | | | | 4.41 | | | 2.13E-03 | | | | | | -2.84 | | 2.20E-02 | | | |  |
| BC036621 | | | | | | LOC644656 | | | | | | | | | | | | | | | 2.02 | | | 3.95E-03 | | | | | | -2.04 | | 6.07E-03 | | | |  |
| AK000901 | | | | | | LOC646719 | | | | | | | | | | | | | | | 3.39 | | | 1.72E-03 | | | | | | -2.16 | | 1.03E-02 | | | |  |
| BC052334 | | | | | | LOC729887 | | | | | | | | | | | | | | | 3.12 | | | 2.42E-03 | | | | | | -2.50 | | 7.33E-03 | | | |  |
| NM_018494 | | | | | | LRDD | | | | | | | | | | | | | | | 2.78 | | | 1.22E-03 | | | | | | -2.24 | | 7.23E-03 | | | |  |
| NM_014665 | | | | | | LRRC14 | | | | | | | | | | | | | | | 2.88 | | | 5.43E-03 | | | | | | -2.05 | | 1.72E-02 | | | |  |
| NM_017527 | | | | | | LY6K | | | | | | | | | | | | | | | 3.22 | | | 3.61E-04 | | | | | | -2.02 | | 9.16E-03 | | | |  |
| BC032375 | | | | | | MAN1B1-AS1 | | | | | | | | | | | | | | | 3.72 | | | 7.35E-03 | | | | | | -2.14 | | 3.69E-02 | | | |  |
| NM_014060 | | | | | | MCTS1 | | | | | | | | | | | | | | | 2.34 | | | 4.98E-04 | | | | | | -2.06 | | 3.72E-03 | | | |  |
| NM_001043229 | | | | | | METTL12 | | | | | | | | | | | | | | | 4.11 | | | 6.44E-04 | | | | | | -2.85 | | 9.94E-03 | | | |  |
| NM_138431 | | | | | | MFSD3 | | | | | | | | | | | | | | | 2.56 | | | 3.19E-03 | | | | | | -2.54 | | 1.60E-02 | | | |  |
| BC007360 | | | | | | MIR503HG | | | | | | | | | | | | | | | 2.34 | | | 6.94E-03 | | | | | | -2.51 | | 1.33E-02 | | | |  |
| NM_199054 | | | | | | MKNK2 | | | | | | | | | | | | | | | 2.38 | | | 1.65E-03 | | | | | | -2.30 | | 3.17E-03 | | | |  |
| ENST00000374865 | | | | | | MRPL50 | | | | | | | | | | | | | | | 2.46 | | | 5.76E-03 | | | | | | -2.26 | | 2.32E-02 | | | |  |
| NM_020998 | | | | | | MST1 | | | | | | | | | | | | | | | 3.19 | | | | 1.01E-02 | | | | | -2.38 | | 3.70E-02 | | | |  |
| NR_002729 | | | | | | MSTP2 | | | | | | | | | | | | | | | 4.10 | | | | 1.22E-02 | | | | | -3.32 | | 4.36E-02 | | | |  |
| NM_002449 | | | | | | MSX2 | | | | | | | | | | | | | | | 2.57 | | | | 4.39E-04 | | | | | -2.42 | | 4.34E-03 | | | |  |
| NM_182501 | | | | | | MTERFD2 | | | | | | | | | | | | | | | 2.38 | | | | 9.07E-04 | | | | | -2.00 | | 6.36E-03 | | | |  |
| NM_013262 | | | | | | MYLIP | | | | | | | | | | | | | | | 4.90 | | | | 2.58E-04 | | | | | -3.53 | | 2.58E-03 | | | |  |
| NM_199461 | | | | | | NANOS1 | | | | | | | | | | | | | | | 4.91 | | | | 4.40E-04 | | | | | -3.35 | | 3.90E-03 | | | |  |
| BC062368 | | | | | | NCBP2-AS2 | | | | | | | | | | | | | | | 3.05 | | | | 7.13E-04 | | | | | -2.28 | | 1.04E-02 | | | |  |
| ENST00000316149 | | | | | | NDUFAF4 | | | | | | | | | | | | | | | 3.45 | | | | 1.21E-03 | | | | | -3.15 | | 5.96E-03 | | | |  |
| NM_005384 | | | | | | NFIL3 | | | | | | | | | | | | | | | 2.10 | | | | 2.06E-03 | | | | | -2.17 | | 2.57E-03 | | | |  |
| ENST00000306750 | | | | | | NFS1 | | | | | | | | | | | | | | | 2.70 | | | | 1.17E-03 | | | | | -2.02 | | 2.44E-02 | | | |  |
| NM_198443 | | | | | | NRN1L | | | | | | | | | | | | | | | 12.40 | | | | 5.80E-04 | | | | | -7.15 | | 7.10E-03 | | | |  |
| ENST00000219169 | | | | | | NUTF2 | | | | | | | | | | | | | | | 2.49 | | | | 2.71E-03 | | | | | -2.38 | | 2.43E-02 | | | |  |
| ENST00000375855 | | | | | | NXNL2 | | | | | | | | | | | | | | | 7.92 | | | | 4.80E-04 | | | | | -4.25 | | 3.79E-03 | | | |  |
| NM_024623 | | | | | | OGFOD2 | | | | | | | | | | | | | | | 2.37 | | | | 2.99E-03 | | | | | -2.21 | | 9.94E-03 | | | |  |
| NM_004561 | | | | | | OVOL1 | | | | | | | | | | | | | | | 3.13 | | | | 4.85E-04 | | | | | -2.24 | | 4.01E-03 | | | |  |
| BC040619 | | | | | | OXR1 | | | | | | | | | | | | | | | 2.80 | | | | 3.03E-03 | | | | | -2.32 | | 4.26E-03 | | | |  |
| NM_024897 | | | | | | PAQR6 | | | | | | | | | | | | | | | 4.68 | | | | 1.05E-03 | | | | | -3.42 | | 5.64E-03 | | | |  |
| NM_152268 | | | | | | PARS2 | | | | | | | | | | | | | | | 2.57 | | | | 1.42E-03 | | | | | -2.06 | | 4.78E-03 | | | |  |
| NM_024028 | | | | | | PCYOX1L | | | | | | | | | | | | | | | 5.26 | | | | 1.96E-03 | | | | | -4.17 | | 6.36E-03 | | | |  |
| NM_001018053 | | | | | | PFKFB2 | | | | | | | | | | | | | | | 3.31 | | | | 3.51E-04 | | | | | -2.71 | | 3.54E-03 | | | |  |
| NM_152595 | | | | | | PGBD4 | | | | | | | | | | | | | | | 2.36 | | | | 2.41E-03 | | | | | -2.01 | | 8.03E-03 | | | |  |
| NM_007350 | | | | | | PHLDA1 | | | | | | | | | | | | | | | 4.22 | | | | 1.27E-03 | | | | | -5.40 | | 3.43E-03 | | | |  |
| NM_003311 | | | | | | PHLDA2 (J) | | | | | | | | | | | | | | | 3.13 | | | | 3.51E-04 | | | | | -2.22 | | 1.04E-02 | | | |  |
| NM_006875 | | | | | | PIM2 | | | | | | | | | | | | | | | 2.52 | | | | 3.47E-03 | | | | | -2.25 | | 1.69E-02 | | | |  |
| NM_022819 | | | | | | PLA2G2F | | | | | | | | | | | | | | | 2.41 | | | | 3.48E-02 | | | | | -2.69 | | ns. | | | |  |
| NM_021127 | | | | | | PMAIP1 (J) | | | | | | | | | | | | | | | 3.64 | | | | 2.01E-03 | | | | | -2.95 | | 4.54E-03 | | | |  |
| NM_022135 | | | | | | POPDC2 | | | | | | | | | | | | | | | 6.97 | | | | 4.56E-04 | | | | | -4.41 | | 1.02E-02 | | | |  |
| NM_001040664 | | | | | | PPAN-P2RY11 | | | | | | | | | | | | | | | 4.41 | | | | 1.32E-03 | | | | | -2.62 | | 3.55E-03 | | | |  |
| BC033025 | | | | | | PPAPDC1B | | | | | | | | | | | | | | | 3.86 | | | | 3.83E-04 | | | | | -2.95 | | 3.79E-03 | | | |  |
| NM_024664 | | | | | | PPCS | | | | | | | | | | | | | | | 2.09 | | | | 1.40E-02 | | | | | -2.06 | | 8.57E-03 | | | |  |
| NM_014068 | | | | | | PSORS1C1 | | | | | | | | | | | | | | | 2.17 | | | | 3.59E-02 | | | | | -2.01 | | ns. | | | |  |
| NM_004158 | | | | | | PSPN | | | | | | | | | | | | | | | 2.47 | | | | 5.64E-03 | | | | | -2.09 | | 1.40E-02 | | | |  |
| NM_000963 | | | | | | PTGS2🟋 (J) | | | | | | | | | | | | | | | 6.75 | | | | 3.11E-02 | | | | | -3.72 | | ns. | | | |  |
| NM_016084 | | | | | | RASD1 | | | | | | | | | | | | | | | 3.57 | | | | 7.48E-03 | | | | | -2.99 | | ns. | | | |  |
| NM_017805 | | | | | | RASIP1 | | | | | | | | | | | | | | | 2.48 | | | | 3.36E-02 | | | | | -2.02 | | ns. | | | |  |
| NM_001007279 | | | | | | RASL10A | | | | | | | | | | | | | | | 2.39 | | | | 4.15E-02 | | | | | -2.31 | | ns. | | | |  |
| NM_206827 | | | | | | RASL11A | | | | | 3.24 | | | | | | | | | | | | | | 5.42E-04 | | | | | -2.38 | | 1.75E-02 | | | |  |
| NM_203393 | | | | | | RBAKDN | | | | | 3.52 | | | | | | | | | | | | | | 1.51E-03 | | | | | -2.37 | | 5.36E-03 | | | |  |
| NM_002928 | | | | | | RGS16 | | | | | 3.28 | | | | | | | | | | | | | | 8.44E-03 | | | | | -2.46 | | 4.05E-02 | | | |  |
| NM_002923 | | | | | | RGS2 (J) | | | | | 3.33 | | | | | | | | | | | | | | 5.63E-03 | | | | | -2.72 | | 1.71E-02 | | | |  |
| BC032393 | | | | | | RNF170 | | | | | 3.21 | | | | | | | | | | | | | | 5.81E-03 | | | | | -2.93 | | 1.64E-02 | | | |  |
| NM_002948 | | | | | | RPL15 | | | | | 2.56 | | | | | | | | | | | | | | 2.17E-03 | | | | | -2.17 | | 1.95E-02 | | | |  |
| ENST00000356931 | | | | | | RPL27A | | | | | 3.74 | | | | | | | | | | | | | | 6.85E-04 | | | | | -2.49 | | 8.34E-03 | | | |  |
| ENST00000360741 | | | | | | RPL37 | | | | | 2.03 | | | | | | | | | | | | | | 1.72E-02 | | | | | -2.05 | | 1.63E-02 | | | |  |
| NM_015659 | | | | | | RSL1D1 | | | | | 3.17 | | | | | | | | | | | | | | 1.92E-03 | | | | | -2.38 | | 8.66E-03 | | | |  |
| NM_020317 | | | | | | RSRP1 | | | | | 3.90 | | | | | | | | | | | | | | 6.21E-04 | | | | | -3.23 | | 4.01E-03 | | | |  |
| NM_002960 | | | | | | S100A3 | | | | | 2.28 | | | | | | | | | | | | | | 1.58E-03 | | | | | -2.20 | | 4.07E-03 | | | |  |
| NM_005870 | | | | | | SAP18 | | | | | 5.03 | | | | | | | | | | | | | | 5.09E-04 | | | | | -3.31 | | 4.21E-03 | | | |  |
| NM_002970 | | | | | | SAT1 | | | | | 2.69 | | | | | | | | | | | | | | 2.92E-03 | | | | | -2.38 | | 1.07E-02 | | | |  |
| NM_016558 | | | | | | SCAND1 | | | | | 3.54 | | | | | | | | | | | | | | 1.39E-03 | | | | | -2.45 | | 1.25E-02 | | | |  |
| NM_015490 | | | | | | SEC31B | | | | | 3.04 | | | | | | | | | | | | | | 2.37E-02 | | | | | -3.81 | | 2.84E-02 | | | |  |
| NM_145204 | | | | | | SENP8 | | | | | 2.72 | | | | | | | | | | | | | | 1.23E-02 | | | | | -2.15 | | ns. | | | |  |
| AK094846 | | | | | | SERF2 | | | | | 2.74 | | | | | | | | | | | | | | 1.72E-03 | | | | | -2.28 | | 1.30E-02 | | | |  |
| NM_013376 | | | | | | SERTAD1 | | | | | 4.86 | | | | | | | | | | | | | | 4.27E-04 | | | | | -3.47 | | 5.01E-03 | | | |  |
| ENST00000367001 | | | | | | SLC30A1 | | | | | 2.21 | | | | | | | | | | | | | | 8.90E-03 | | | | | -2.16 | | 4.62E-03 | | | |  |
| NM_005985 | | | | | | SNAI1 | | | | | 6.44 | | | | | | | | | | | | | | 4.27E-04 | | | | | -3.44 | | 4.80E-02 | | | |  |
| NM_003068 | | | | | | SNAI2 🟋 | | | | | 2.39 | | | | | | | | | | | | | | 7.84E-03 | | | | | -2.15 | | 2.70E-02 | | | |  |
| NR_003138 | | | | | | SNHG10 | | | | | 3.36 | | | | | | | | | | | | | | 2.94E-03 | | | | | -2.99 | | 1.32E-02 | | | |  |
| AF130050 | | | | | | SNHG20 | | | | | 3.23 | | | | | | | | | | | | | | 4.01E-04 | | | | | -2.52 | | 3.81E-03 | | | |  |
| BC002724 | | | | | | SNX5 | | | | | 16.21 | | | | | | | | | | | | | | 2.67E-04 | | | | | -5.28 | | 3.38E-03 | | | |  |
| NM_003745 | | | | | | SOCS1 | | | | | 3.83 | | | | | | | | | | | | | | 9.01E-04 | | | | | -2.02 | | 1.46E-02 | | | |  |
| NR_002330 | | | | | | ST7OT1 | | | | | 6.43 | | | | | | | | | | | | | | 3.25E-04 | | | | | -3.75 | | 5.32E-03 | | | |  |
| NM_003192 | | | | | | TBCC | | | | | 2.79 | | | | | | | | | | | | | | 4.60E-04 | | | | | -2.24 | | 3.93E-03 | | | |  |
| BC032312 | | | | | | TBRG1 | | | | | 2.62 | | | | | | | | | | | | | | 7.75E-04 | | | | | -2.23 | | 9.94E-03 | | | |  |
| NM_020147 | | | | | | THAP10 | | | | | 3.94 | | | | | | | | | | | | | | 5.64E-04 | | | | | -3.04 | | 8.41E-03 | | | |  |
| NM_031435 | | | | | | THAP2 | | | | | 4.48 | | | | | | | | | | | | | | 4.12E-04 | | | | | -4.18 | | 3.45E-03 | | | |  |
| NM_017736 | | | | | | THUMPD1 | | | | | 2.43 | | | | | | | | | | | | | | 4.36E-03 | | | | | -2.07 | | 1.72E-02 | | | |  |
| NM_003273 | | | | | | TM7SF2 | | | | | 2.61 | | | | | | | | | | | | | | 1.72E-03 | | | | | -2.12 | | 1.50E-02 | | | |  |
| AK126594 | | | | TMA7 | | | | | | | | | | | | 2.60 | | | | | | | | | 1.15E-03 | | | | | -2.26 | | 6.06E-03 | | | |  |
| AK090478 | | | | TMC8 | | | | | | | | | | | | 3.81 | | | | | | | | | 1.05E-02 | | | | | -2.54 | | 4.26E-02 | | | |  |
| NM_153342 | | | | TMEM150A | | | | | | | | | | | | 3.03 | | | | | | | | | 5.02E-04 | | | | | -2.18 | | 6.44E-03 | | | |  |
| NM_030577 | | | | TMEM177 | | | | | | | | | | | | 2.67 | | | | | | | | | 9.09E-04 | | | | | -2.24 | | 3.54E-03 | | | |  |
| NM_144638 | | | | TMEM42 | | | | | | | | | | | | 2.70 | | | | | | | | | 2.11E-03 | | | | | -2.13 | | 5.64E-03 | | | |  |
| NM_001042463 | | | | TMEM80 | | | | | | | | | | | | 2.96 | | | | | | | | | 7.50E-04 | | | | | -2.44 | | 4.45E-03 | | | |  |
| NM_203411 | | | | TMEM88 | | | | | | | | | | | | 13.91 | | | | | | | | | 1.01E-03 | | | | | -5.75 | | 4.47E-03 | | | |  |
| NM_005749 | | | | TOB1 | | | | | | | | | | | | 3.34 | | | | | | | | | 3.61E-04 | | | | | -2.42 | | 3.77E-03 | | | |  |
| NM_016381 | | | | TREX1 | | | | | | | | | | | | 4.48 | | | | | | | | | 8.18E-04 | | | | | -2.90 | | 5.00E-03 | | | |  |
| NM_025195 | | | | TRIB1 | | | | | | | | | | | | 3.34 | | | | | | | | | 2.20E-03 | | | | | -2.17 | | ns. | | | |  |
| NM_213590 | | | | TRIM13 | | | | | | | | | | | | 2.28 | | | | | | | | | 3.09E-02 | | | | | -2.11 | | ns. | | | |  |
| NM_033017 | | | | TRIM4 | | | | | | | | | | | | 2.34 | | | | | | | | | 1.92E-03 | | | | | -2.30 | | 2.90E-03 | | | |  |
| NM_001069 | | | | TUBB2A | | | | | | | | | | | | 3.45 | | | | | | | | | 6.89E-04 | | | | | -2.12 | | 1.22E-02 | | | |  |
| NM_003353 | | | | UCN | | | | | | | | | | | | 3.54 | | | | | | | | | 4.55E-04 | | | | | -2.84 | | 3.14E-03 | | | |  |
| NM_182566 | | | | VMO1 | | | | | | | | | | | | 3.79 | | | | | | | | | 9.10E-04 | | | | | -2.89 | | 4.23E-03 | | | |  |
| NM_152718 | | | | VWCE | | | | | | | | | | | | 3.48 | | | | | | | | | 2.93E-03 | | | | | -2.02 | | 1.28E-02 | | | |  |
| BC002482 | | | | WDR55 | | | | | | | | | | | | 2.72 | | | | | | | | | 7.91E-04 | | | | | -2.22 | | 3.48E-03 | | | |  |
| NM_018566 | | | | YOD1 | | | | | | | | | | | | 4.55 | | | | | | | | | 6.48E-04 | | | | | -2.68 | | 1.02E-02 | | | |  |
| NM_175907.4 | | | | ZADH2 | | | | | | | | | | | | 5.58 | | | | | | | | | 4.10E-04 | | | | | -4.15 | | 3.43E-03 | | | |  |
| NM_014950 | | | | ZBTB1 | | | | | | | | | | | | 3.15 | | | | | | | | | 3.40E-03 | | | | | -2.63 | | 1.95E-02 | | | |  |
| NM_014830 | | | | ZBTB39 | | | | | | | | | | | | 2.87 | | | | | | | | | 4.98E-04 | | | | | -2.20 | | 2.51E-03 | | | |  |
| NM_006626 | | | | ZBTB6 | | | | | | | | | | | | 2.58 | | | | | | | | | 1.64E-02 | | | | | -2.39 | | 8.64E-03 | | | |  |
| NM_033089 | | | | ZCCHC3 | | | | | | | | | | | | 2.48 | | | | | | | | | 4.16E-04 | | | | | -2.01 | | 2.57E-03 | | | |  |
| AK056630 | | | | ZKSCAN1 | | | | | | | | | | | | 2.55 | | | | | | | | | 1.35E-02 | | | | | -2.38 | | 2.12E-02 | | | |  |
| NM_001012981 | | | | ZKSCAN2 | | | | | | | | | | | | 2.64 | | | | | | | | | 6.44E-04 | | | | | -2.32 | | 7.45E-03 | | | |  |
| NM_032265 | | | | ZMYND15 | | | | | | | | | | | | 3.30 | | | | | | | | | 6.97E-04 | | | | | -2.21 | | 4.25E-03 | | | |  |
| NM_005773 | | | | ZNF256 | | | | | | | | | | | | 2.30 | | | | | | | | | 7.50E-04 | | | | | -2.00 | | 7.99E-03 | | | |  |
| NM_003417 | | | | ZNF264 | | | | | | | | | | | | 3.07 | | | | | | | | | 9.82E-03 | | | | | -2.88 | | 5.83E-03 | | | |  |
| NM_145288 | | | | ZNF296 | | | | | | | | | | | | 3.43 | | | | | | | | | 2.34E-03 | | | | | -2.34 | | 3.11E-03 | | | |  |
| NM_018443 | | | | ZNF302 | | | | | | | | | | | | 2.26 | | | | | | | | | 1.22E-03 | | | | | -2.08 | | 4.78E-03 | | | |  |
| AF533250 | | | | ZNF397 | | | | | | | | | | | | 2.25 | | | | | | | | | 2.93E-03 | | | | | -2.08 | | 2.46E-02 | | | |  |
| NM_024691 | | | | ZNF419 | | | | | | | | | | | | 3.09 | | | | | | | | | 6.33E-04 | | | | | -2.32 | | 5.31E-03 | | | |  |
| NM_001001661 | | | | ZNF425 | | | | | | | | | | | | 7.21 | | | | | | | | | 4.65E-04 | | | | | -3.57 | | 1.75E-02 | | | |  |
| NM_001077195 | | | | ZNF436 | | | | | | | | | | | 2.02 | | | | | | | | | | 1.53E-03 | | | | -2.08 | | | 8.84E-03 | | | |  |
| NM_152303 | | | | ZNF554 | | | | | | | | | | | 2.70 | | | | | | | | | | 3.24E-03 | | | | -2.30 | | | 3.45E-03 | | | |  |
| NM_016202 | | | | ZNF580 | | | | | | | | | | | 2.91 | | | | | | | | | | 4.96E-03 | | | | -2.06 | | | ns. | | | |  |
| NM_025040 | | | | ZNF614 | | | | | | | | | | | 2.89 | | | | | | | | | | 3.68E-04 | | | | -2.12 | | | 2.80E-03 | | | |  |
| NM_145295 | | | | ZNF627 | | | | | | | | | | | 2.03 | | | | | | | | | | 2.13E-03 | | | | -2.32 | | | 5.21E-03 | | | |  |
| NM_152458 | | | | ZNF785 | | | | | | | | | | | 2.56 | | | | | | | | | | 2.14E-02 | | | | -2.18 | | | 2.50E-02 | | | |  |
| **List of 738 downregulated CPD-dependent genes 6 h after UVB exposure.** | | | | | | | | | | | | | | | | | | | | | | | | | | | | | | | | | | | |  |
| NM_005157 | | | | ABL1 | | | | | | | | | | | -2.06 | | | | | | | | | | 1.72E-02 | | | | 2.05 | | | 3.65E-02 | | | |  |
| NM_145804 | | | | ABTB2 | | | | | | | | | | | -5.54 | | | | | | | | | | 8.65E-04 | | | | 5.80 | | | 3.45E-03 | | | |  |
| NM_012287 | | | | ACAP2 | | | | | | | | | | | -3.28 | | | | | | | | | | 1.17E-03 | | | | 2.26 | | | 1.02E-02 | | | |  |
| NM_001105 | | | | ACVR1 | | | | | | | | | | | -3.81 | | | | | | | | | | 1.06E-03 | | | | 3.23 | | | 7.10E-03 | | | |  |
| NM_153207 | | | | AEBP2 | | | | | | | | | | | -3.23 | | | | | | | | | | 5.06E-04 | | | | 2.42 | | | 4.07E-03 | | | |  |
| NM_014423 | | | | AFF4 | | | | | | | | | | | -2.82 | | | | | | | | | | 9.52E-04 | | | | 2.71 | | | 3.93E-03 | | | |  |
| NM_031946 | | | | AGAP3 | | | | | | | | | | | -2.27 | | | | | | | | | | 2.87E-03 | | | | 2.01 | | | 2.12E-02 | | | |  |
| NM_015446 | | | | AHCTF1 | | | | | | | | | | | -3.36 | | | | | | | | | | 4.12E-04 | | | | 3.21 | | | 4.80E-03 | | | |  |
| NM_018836 | | | | AJAP1 | | | | | | | | | | | -3.18 | | | | | | | | | | 6.74E-03 | | | | 2.44 | | | ns. | | | |  |
| NM_007202 | | | | AKAP10 | | | | | | | | | | | -2.42 | | | | | | | | | | 1.62E-03 | | | | 2.64 | | | 5.59E-03 | | | |  |
| NM_006738 | | | | AKAP13 | | | | | | | | | | | -17.87 | | | | | | | | | | 3.61E-04 | | | | 12.79 | | | 3.11E-03 | | | |  |
| NM_017749 | | | | AMBRA1 | | | | | | | | | | | -4.63 | | | | | | | | | | 3.67E-04 | | | | 4.43 | | | 2.58E-03 | | | |  |
| NM_130847 | | | | AMOTL1 | | | | | | | | | | | -3.36 | | | | | | | | | | 4.98E-04 | | | | 3.55 | | | 5.15E-03 | | | |  |
| NM_017747 | | | | ANKHD1 | | | | | | | | | | | -2.71 | | | | | | | | | | 5.57E-04 | | | | 2.36 | | | 4.26E-03 | | | |  |
| NM_013275 | | | | ANKRD11 | | | | | | | | | | | -4.53 | | | | | | | | | | 9.87E-04 | | | | 3.78 | | | 4.01E-03 | | | |  |
| NM_015208 | | | | ANKRD12 | | | | | | | | | | | -3.56 | | | | | | | | | | 4.74E-04 | | | | 2.58 | | | 2.60E-03 | | | |  |
| AL136717 | | | | ANKRD13C | | | | | | | | | | | -3.44 | | | | | | | | | | 8.32E-04 | | | | 2.92 | | | 5.20E-03 | | | |  |
| NM_032217 | | | | ANKRD17 | | | | | | | | | | | -2.65 | | | | | | | | | | 4.95E-04 | | | | 2.20 | | | 3.43E-03 | | | |  |
| NM_032290 | | | | ANKRD32 | | | | | | | | | | | -2.55 | | | | | | | | | | 6.59E-04 | | | | 2.38 | | | 4.05E-03 | | | |  |
| BC018597 | | | | ANKRD33B | | | | | | | | | | | -2.20 | | | | | | | | | | 7.85E-03 | | | | 2.29 | | | 2.42E-02 | | | |  |
| NM_020970 | | | | ANKRD36B | | | | | | | | | | | -2.57 | | | | | | | | | | 8.09E-03 | | | | 2.21 | | | 1.83E-02 | | | |  |
| NM_015245 | | | | ANKS1A | | | | | | | | | | | -5.10 | | | | | | | | | | 5.08E-04 | | | | 4.08 | | | 3.11E-03 | | | |  |
| NM_001030007 | | | | AP1G1 | | | | | | | | | | | -2.69 | | | | | | | | | | 4.60E-04 | | | | 2.86 | | | 3.76E-03 | | | |  |
| NM_015230 | | | | ARAP2 | | | | | | | | | | | -5.30 | | | | | | | | | | 6.34E-04 | | | | 3.72 | | | 3.81E-03 | | | |  |
| NM_001039479 | | | | AREL1 | | | | | | | | | | | -2.65 | | | | | | | | | | 1.52E-03 | | | | 2.23 | | | 1.48E-02 | | | |  |
| NM_006421 | | | | ARFGEF1 | | | | | | | | | | | -3.41 | | | | | | | | | | 2.30E-03 | | | | 2.39 | | | 2.44E-02 | | | |  |
| NM_018287 | | | | | | ARHGAP12 | | | | | | | | | | -6.06 | | | | | | | | | 3.90E-04 | | | | | | 4.51 | 3.45E-03 | | | |  |
| NM_001006634 | | | | | | ARHGAP17 | | | | | | | | | | -2.62 | | | | | | | | | 3.36E-03 | | | | | | 2.75 | 5.26E-03 | | | |  |
| NM_020824 | | | | | | ARHGAP21 | | | | | | | | | | -3.58 | | | | | | | | | 4.32E-04 | | | | | | 3.02 | 3.93E-03 | | | |  |
| NM_025251 | | | | | | ARHGAP39 | | | | | | | | | | -3.93 | | | | | | | | | 3.61E-04 | | | | | | 3.43 | 2.60E-03 | | | |  |
| NM_018125 | | | | | | ARHGEF10L | | | | | | | | | | -4.59 | | | | | | | | | 6.44E-03 | | | | | | 3.06 | ns. | | | |  |
| NM_003899 | | | | | | ARHGEF7 | | | | | | | | | | -4.81 | | | | | | | | | 5.43E-04 | | | | | | 4.14 | 3.54E-03 | | | |  |
| NM_006015 | | | | | | ARID1A | | | | | | | | | | -2.98 | | | | | | | | | 5.03E-03 | | | | | | 2.89 | 4.60E-03 | | | |  |
| NM_152641 | | | | | | ARID2 | | | | | | | | | | -7.95 | | | | | | | | | 5.64E-04 | | | | | | 6.59 | 3.55E-03 | | | |  |
| NM_016374 | | | | | | ARID4B | | | | | | | | | | -8.32 | | | | | | | | | 7.10E-04 | | | | | | 6.93 | 4.80E-03 | | | |  |
| NM_032199 | | | | | | ARID5B | | | | | | | | | | -4.52 | | | | | | | | | 3.14E-03 | | | | | | 2.87 | 2.93E-02 | | | |  |
| AK000336 | | | | | | ARIH1 | | | | | | | | | | -2.50 | | | | | | | | | 6.44E-03 | | | | | | 2.26 | 9.94E-03 | | | |  |
| NM_014154 | | | | | | ARMC8 | | | | | | | | | | -5.48 | | | | | | | | | 7.42E-04 | | | | | | 3.46 | 3.79E-03 | | | |  |
| NM_001030273 | | | | | | ARNTL | | | | | | | | | | -3.35 | | | | | | | | | 5.84E-03 | | | | | | 3.19 | 2.30E-02 | | | |  |
| NM_024590 | | | | | | ARSJ | | | | | | | | | | -2.89 | | | | | | | | | 3.68E-04 | | | | | | 2.23 | 4.67E-03 | | | |  |
| NM_003887 | | | | | | ASAP2 | | | | | | | | | | -4.02 | | | | | | | | | 5.12E-04 | | | | | | 2.89 | 5.64E-03 | | | |  |
| NM_018489 | | | | | | ASH1L | | | | | | | | | | -2.43 | | | | | | | | | 5.20E-03 | | | | | | 2.12 | 3.15E-02 | | | |  |
| NM_015338 | | | | | | ASXL1 | | | | | | | | | | -3.35 | | | | | | | | | 1.22E-03 | | | | | | 3.01 | 5.96E-03 | | | |  |
| NM_018263 | | | | | | ASXL2 | | | | | | | | | | -3.15 | | | | | | | | | 6.44E-04 | | | | | | 2.67 | 5.59E-03 | | | |  |
| ENST00000238789 | | | | | | ATAD2B | | | | | | | | | | -5.23 | | | | | | | | | 4.29E-04 | | | | | | 3.54 | 3.79E-03 | | | |  |
| NM_001880 | | | | | | ATF2 | | | | | | | | | | -3.21 | | | | | | | | | 5.08E-04 | | | | | | 2.64 | 3.54E-03 | | | |  |
| NM_018179 | | | | | | ATF7IP | | | | | | | | | | -2.74 | | | | | | | | | 4.52E-04 | | | | | | 2.32 | 5.34E-03 | | | |  |
| NM_032852 | | | | | | ATG4C | | | | | | | | | | -2.22 | | | | | | | | | 4.82E-04 | | | | | | 2.16 | 4.25E-03 | | | |  |
| ENST00000378954 | | | | | | ATL2 | | | | | | | | | | -3.86 | | | | | | | | | 7.73E-04 | | | | | | 2.88 | 4.94E-03 | | | |  |
| NM_139321 | | | | | | ATRN | | | | | | | | | | -4.22 | | | | | | | | | 4.77E-04 | | | | | | 2.71 | 5.41E-03 | | | |  |
| NM_002973 | | | | | | ATXN2 | | | | | | | | | | -2.86 | | | | | | | | | 2.91E-03 | | | | | | 2.68 | 2.60E-03 | | | |  |
| NM_015570 | | | | | | AUTS2 | | | | | | | | | | -3.46 | | | | | | | | | 3.90E-03 | | | | | | 2.73 | 9.16E-03 | | | |  |
| NM_015060 | | | | | | AVL9 | | | | | | | | | | -3.29 | | | | | | | | | 5.43E-04 | | | | | | 2.82 | 4.14E-03 | | | |  |
| NM_004776 | | | | | | B4GALT5 | | | | | | | | | | -3.08 | | | | | | | | | 3.25E-04 | | | | | | 2.54 | 2.58E-03 | | | |  |
| AF527552 | | | | | | BAGE | | | | | | | | | | -2.67 | | | | | | | | | 1.09E-03 | | | | | | 2.33 | 5.21E-03 | | | |  |
| NM_017451 | | | | | | BAIAP2 | | | | | | | | | | -2.16 | | | | | | | | | 2.04E-02 | | | | | | 2.32 | 3.82E-02 | | | |  |
| NM_079837 | | | | | | BANP | | | | | | | | | | -3.04 | | | | | | | | | 7.03E-04 | | | | | | 2.73 | 3.45E-03 | | | |  |
| NM_000465 | | | | | | BARD1 | | | | | | | | | | -2.36 | | | | | | | | | 7.51E-04 | | | | | | 2.43 | 3.45E-03 | | | |  |
| ENST00000320547 | | | | | | BAT2L | | | | | | -2.28 | | | | | | | | | | | | | 1.17E-02 | | | | | | 2.05 | 1.19E-02 | | | |  |
| NM_013448 | | | | | | BAZ1A | | | | | | -3.91 | | | | | | | | | | | | | 4.45E-04 | | | | | | 3.03 | 4.34E-03 | | | |  |
| NM_032408 | | | | | | BAZ1B | | | | | | -2.21 | | | | | | | | | | | | | 3.04E-03 | | | | | | 2.11 | 7.15E-03 | | | |  |
| NM_003567 | | | | | | BCAR3 (J) | | | | | | -5.70 | | | | | | | | | | | | | 4.60E-04 | | | | | | 6.26 | 3.43E-03 | | | |  |
| NM_022893 | | | | | | BCL11A | | | | | | -4.78 | | | | | | | | | | | | | 4.78E-04 | | | | | | 3.66 | ns. | | | |  |
| NM_138578 | | | | | | BCL2L1 🟋 | | | | | | -2.62 | | | | | | | | | | | | | 1.61E-03 | | | | | | 2.27 | 8.84E-03 | | | |  |
| NM_018429 | | | | | | BDP1 | | | | | | -4.20 | | | | | | | | | | | | | 3.61E-04 | | | | | | 3.56 | 2.69E-03 | | | |  |
| NM_016252 | | | | | | BIRC6 | | | | | | -2.58 | | | | | | | | | | | | | 1.11E-03 | | | | | | 2.07 | 4.43E-03 | | | |  |
| NM_000057 | | | | | | BLM | | | | | | -3.03 | | | | | | | | | | | | | 3.25E-04 | | | | | | 2.81 | 2.07E-03 | | | |  |
| NM_004329 | | | | | | BMPR1A | | | | | | -7.39 | | | | | | | | | | | | | 3.25E-04 | | | | | | 4.95 | 1.51E-03 | | | |  |
| NM_001203 | | | | | | BMPR1B | | | | | | -11.43 | | | | | | | | | | | | | 3.51E-04 | | | | | | 5.83 | ns. | | | |  |
| NM_182641 | | | | | | BPTF | | | | | | -3.70 | | | | | | | | | | | | | 6.62E-04 | | | | | | 2.70 | 3.70E-03 | | | |  |
| NM_145685 | | | | | | BRF1 | | | | | | -2.11 | | | | | | | | | | | | | 1.66E-02 | | | | | | 2.15 | 6.20E-03 | | | |  |
| NM_020180 | | | | | | BRUNOL4 | | | | | | -2.00 | | | | | | | | | | | | | 3.01E-03 | | | | | | 2.17 | 7.38E-03 | | | |  |
| NM_018963 | | | | | | BRWD1 | | | | | | -3.49 | | | | | | | | | | | | | 7.08E-04 | | | | | | 2.86 | 6.71E-03 | | | |  |
| NM_003972 | | | | | | BTAF1 | | | | | | -3.29 | | | | | | | | | | | | | 4.64E-03 | | | | | | 2.75 | 9.59E-03 | | | |  |
| NM_032320 | | | | | | BTBD10 | | | | | | -2.74 | | | | | | | | | | | | | 1.03E-03 | | | | | | 2.37 | 3.54E-03 | | | |  |
| NM_001002860 | | | | | | BTBD7 | | | | | | -2.57 | | | | | | | | | | | | | 1.64E-02 | | | | | | 2.64 | 3.36E-02 | | | |  |
| NM_033637 | | | | | | BTRC | | | | | | -5.27 | | | | | | | | | | | | | 4.33E-04 | | | | | | 3.60 | 3.79E-03 | | | |  |
| NM_015652 | | | | | | C10orf12 | | | | | | -4.00 | | | | | | | | | | | | | 5.80E-04 | | | | | | 3.28 | 4.50E-03 | | | |  |
| ENST00000380270 | | | | | | C10orf18 | | | | | | -2.12 | | | | | | | | | | | | | 1.46E-03 | | | | | | 2.17 | 3.14E-03 | | | |  |
| ENST00000369889 | | | | | | C10orf26 | | | | | | -2.81 | | | | | | | | | | | | | 1.92E-03 | | | | | | 2.48 | 6.80E-03 | | | |  |
| NM_153810 | | | | | | C10orf46 | | | | | | -2.22 | | | | | | | | | | | | | 1.58E-02 | | | | | | 2.32 | 1.16E-02 | | | |  |
| NM_020193 | | | | | | C11orf30 | | | | | | -3.31 | | | | | | | | | | | | | 5.57E-04 | | | | | | 2.97 | 4.07E-03 | | | |  |
| NM_020374 | | | | | | C12orf4 | | | | | | -2.34 | | | | | | | | | | | | | 6.85E-04 | | | | | | 2.44 | 3.79E-03 | | | |  |
| NM_194278 | | | | | | C14orf43 | | | | | | -2.16 | | | | | | | | | | | | | 8.22E-04 | | | | | | 2.11 | 2.57E-03 | | | |  |
| NM_145055 | | | | | | C18orf25 | | | | | | -2.78 | | | | | | | | | | | | | 1.44E-03 | | | | | | 2.96 | 7.72E-03 | | | |  |
| NM_020156 | | | | | | C1GALT1 | | | | | | -2.47 | | | | | | | | | | | | | 7.13E-03 | | | | | | 2.50 | 1.80E-02 | | | |  |
| NM_016227 | | | | | | C1orf9 | | | | | | -2.44 | | | | | | | | | | | | | 7.61E-04 | | | | | | 2.32 | 3.81E-03 | | | |  |
| NM_001009608 | | | | | | C20orf94 | | | | | | -3.04 | | | | | | | | | | | | | 4.88E-04 | | | | | | 2.41 | 3.60E-03 | | | |  |
| NM_178496 | | | | | | C3orf59 | | | | | | -3.48 | | | | | | | | | | | | | 1.05E-03 | | | | | | 3.44 | 4.67E-03 | | | |  |
| NM_198468 | | | | | | C6orf167 | | | | | | -3.79 | | | | | | | | | | | | | 1.50E-03 | | | | | | 2.60 | 1.47E-02 | | | |  |
| ENST00000374049 | | | | | | C9orf91 | | | | | | -2.06 | | | | | | | | | | | | | 5.36E-03 | | | | | | 2.02 | 8.64E-03 | | | |  |
| NM_016289 | | | | CAB39 | | | | | | | | | | | | | | -2.28 | | | | | | | 5.12E-03 | | | | | | 2.12 | 1.09E-02 | | | |  |
| NM_138375 | | | | CABLES1 | | | | | | | | | | | | | | -3.75 | | | | | | | 4.96E-03 | | | | | | 3.70 | 1.40E-02 | | | |  |
| NM_203459 | | | | CAMSAP1L1 | | | | | | | | | | | | | | -3.62 | | | | | | | 5.20E-03 | | | | | | 2.91 | 1.04E-02 | | | |  |
| NM_001002259 | | | | CAPRIN2 | | | | | | | | | | | | | | -2.29 | | | | | | | 8.39E-04 | | | | | | 2.13 | 1.25E-02 | | | |  |
| NM_005188 | | | | CBL | | | | | | | | | | | | | | -2.12 | | | | | | | 5.12E-03 | | | | | | 2.09 | 7.35E-03 | | | |  |
| NM_170662 | | | | CBLB | | | | | | | | | | | | | | -5.70 | | | | | | | 1.95E-03 | | | | | | 5.12 | 8.64E-03 | | | |  |
| NM_144978 | | | | CCDC138 | | | | | | | | | | | | | | -2.86 | | | | | | | 2.57E-04 | | | | | | 2.46 | 2.41E-03 | | | |  |
| BC110042 | | | | CCDC85C | | | | | | | | | | | | | | -2.63 | | | | | | | 4.25E-03 | | | | | | 2.35 | 7.03E-03 | | | |  |
| NM_019044 | | | | CCDC93 | | | | | | | | | | | | | | -2.70 | | | | | | | 6.47E-04 | | | | | | 2.77 | 3.54E-03 | | | |  |
| NM_003718 | | | | CDC2L5 | | | | | | | | | | | | | | -3.94 | | | | | | | 5.58E-04 | | | | | | 3.36 | 4.01E-03 | | | |  |
| NM_001038707 | | | | CDC42SE1 | | | | | | | | | | | | | | -2.27 | | | | | | | 7.26E-04 | | | | | | 2.18 | 3.11E-03 | | | |  |
| NM_020240 | | | | CDC42SE2 | | | | | | | | | | | | | | -4.17 | | | | | | | 2.80E-04 | | | | | | 3.32 | 2.88E-03 | | | |  |
| NM_024529 | | | | CDC73 | | | | | | | | | | | | | | -3.42 | | | | | | | 4.10E-04 | | | | | | 2.40 | 9.63E-03 | | | |  |
| NM_016507 | | | | CDK12 | | | | | | | | | | | | | | -2.68 | | | | | | | 4.32E-04 | | | | | | 2.59 | 3.45E-03 | | | |  |
| NM_001799 | | | | CDK7 | | | | | | | | | | | | | | -2.24 | | | | | | | 2.25E-03 | | | | | | 2.01 | 3.17E-03 | | | |  |
| NM_001260 | | | | CDK8 | | | | | | | | | | | | | | -3.69 | | | | | | | 4.61E-04 | | | | | | 3.23 | 2.07E-03 | | | |  |
| NM_170752 | | | | CDYL | | | | | | | | | | | | | | -6.22 | | | | | | | 3.68E-04 | | | | | | 5.27 | 3.45E-03 | | | |  |
| NM_006560 | | | | CELF1 | | | | | | | | | | | | | | -2.67 | | | | | | | 4.98E-04 | | | | | | 2.58 | 2.58E-03 | | | |  |
| NM_001809 | | | | CENPA | | | | | | | | | | | | | | -3.28 | | | | | | | 3.61E-04 | | | | | | 2.04 | 4.21E-03 | | | |  |
| NM_001812 | | | | CENPC1 | | | | | | | | | | | | | | -3.61 | | | | | | | 5.10E-04 | | | | | | 2.55 | 6.81E-03 | | | |  |
| NM_025009 | | | | CEP135 | | | | | | | | | | | | | | -2.82 | | | | | | | 3.25E-04 | | | | | | 2.31 | 2.58E-03 | | | |  |
| NM_014985 | | | | CEP152 | | | | | | | | | | | | | | -2.63 | | | | | | | 3.51E-03 | | | | | | 2.73 | 7.89E-03 | | | |  |
| NM_032142 | | | | CEP192 | | | | | | | | | | | | | | -2.53 | | | | | | | 1.34E-03 | | | | | | 2.43 | 8.34E-03 | | | |  |
| NM_014810 | | | | CEP350 | | | | | | | | | | | | | | -5.22 | | | | | | | 4.32E-04 | | | | | | 4.16 | 3.43E-03 | | | |  |
| NM_001270 | | | | CHD1 | | | | | | | | | | | | | | -2.74 | | | | | | | 1.34E-03 | | | | | | 2.72 | 2.57E-03 | | | |  |
| NM_001271 | | | | CHD2 | | | | | | | | | | | | | | -3.30 | | | | | | | 4.03E-04 | | | | | | 2.63 | 3.43E-03 | | | |  |
| NM_032221 | | | | CHD6 | | | | | | | | | | | | | | -2.95 | | | | | | | 4.16E-04 | | | | | | 2.18 | 3.45E-03 | | | |  |
| NM_017780 | | | | CHD7 | | | | | | | | | | | | | | -7.38 | | | | | | | 6.25E-04 | | | | | | 6.46 | 3.47E-03 | | | |  |
| NM_025134 | | | | CHD9 | | | | | | | | | | | | | | -8.20 | | | | | | | 2.80E-04 | | | | | | 4.29 | 2.80E-03 | | | |  |
| NM_012110 | | | | CHIC2 | | | | | | | | | | | | | | -2.86 | | | | | | | 4.28E-03 | | | | | | 2.38 | 3.03E-02 | | | |  |
| NM_018413 | | | | CHST11 | | | | | | | | | | | | | | -3.11 | | | | | | | 2.72E-03 | | | | | | 2.41 | 9.82E-03 | | | |  |
| NM_015892 | | | | CHST15 | | | | | | | | | | | | | | -3.93 | | | | | | | 1.28E-03 | | | | | | 4.45 | 4.31E-03 | | | |  |
| NM_014918 | | | | CHSY1 | | | | | | | | | | | | | | -2.01 | | | | | | | 9.15E-03 | | | | | | 2.17 | 1.22E-02 | | | |  |
| NM_015226 | | | | CLEC16A | | | | | | | | | | | | | | -3.43 | | | | | | | 1.78E-03 | | | | | | 2.46 | 3.14E-03 | | | |  |
| NM_002956 | | | | CLIP1 | | | | | | | | | | | | | | -3.61 | | | | | | | 4.52E-04 | | | | | | 2.21 | 3.79E-03 | | | |  |
| NM_024692 | | | | CLIP4 | | | | | | | | | | | | | | -3.30 | | | | | | | 1.34E-03 | | | | | | 2.26 | 1.90E-02 | | | |  |
| NM_004898 | | | | CLOCK | | | | | | | | | | | | | | -3.98 | | | | | | | 4.03E-04 | | | | | | 3.26 | 3.43E-03 | | | |  |
| NM_014515 | | | | CNOT2 | | | | | | | | | | | | | | -3.32 | | | | | | | 1.05E-03 | | | | | | 2.83 | 3.45E-03 | | | |  |
| NM_013316 | | | | CNOT4 | | | | | | | | | | | | | | -4.47 | | | | | | | 4.09E-04 | | | | | | 3.93 | 3.54E-03 | | | |  |
| NM_015455 | | | | CNOT6 | | | | | | | | | | | | | | -2.50 | | | | | | | 4.69E-04 | | | | | | 2.09 | 4.40E-03 | | | |  |
| NM_144571 | | | | CNOT6L | | | | | | | | | | | | | | -3.02 | | | | | | | 3.68E-04 | | | | | | 2.09 | 7.78E-03 | | | |  |
| NM_152609 | | | | CNST | | | | | | | | | | | | | | -2.92 | | | | | | | 6.40E-03 | | | | | | 2.20 | 6.74E-03 | | | |  |
| NM_031431 | | | | COG3 | | | | | | | | | | | | | | -2.37 | | | | | | | 5.29E-03 | | | | | | 2.19 | 4.11E-02 | | | |  |
| AK021957 | | | | COL27A1 | | | | | | | | | | | | | | -2.34 | | | | | | | 1.57E-02 | | | | | | 2.38 | 6.36E-03 | | | |  |
| NM_005713 | | | | COL4A3BP | | | | | | | | | | | | | | -3.41 | | | | | | | 4.55E-04 | | | | | | 2.59 | 4.80E-03 | | | |  |
| NM_001303 | | | | COX10 | | | | | | | | | | | | | | -3.68 | | | | | | | 2.80E-04 | | | | | | 2.98 | 2.58E-03 | | | |  |
| NM_134442 | | | | CREB1 | | | | | | | | | | | | | | -2.93 | | | | | | | 6.85E-04 | | | | | | 2.32 | 3.79E-03 | | | |  |
| BC063666 | | | | CREB3L2 | | | | | | | | | | | | | | -2.75 | | | | | | | 8.99E-04 | | | | | | 2.31 | 7.11E-03 | | | |  |
| NM_004380 | | | | CREBBP | | | | | | | | | | | | | | -3.20 | | | | | | | 1.68E-03 | | | | | | 2.99 | 3.43E-03 | | | |  |
| NM_022769 | | | | CRTC3 | | | | | | | | | | | | | | -3.09 | | | | | | | 1.49E-03 | | | | | | 2.76 | 5.94E-03 | | | |  |
| NM_203481 | | | | CTBP2 | | | | | | | | | | | | | | -2.40 | | | | | | | 1.32E-03 | | | | | | 2.09 | 1.85E-02 | | | |  |
| NM_006565 | | | | CTCF | | | | | | | | | | | | | | -2.47 | | | | | | | 4.02E-03 | | | | | | 2.52 | 3.45E-03 | | | |  |
| NM_016396 | | | | CTDSPL2 | | | | | | | | | | | | | | -2.86 | | | | | | | 5.72E-04 | | | | | | 2.27 | 3.54E-03 | | | |  |
| NM_018704 | | | | CTTNBP2NL | | | | | | | | | | | | | | -2.69 | | | | | | | 2.86E-03 | | | | | | 2.78 | 1.61E-02 | | | |  |
| NM_003592 | | | | CUL1 | | | | | | | | | | | | | | -3.47 | | | | | | | 4.98E-04 | | | | | | 2.84 | 3.62E-03 | | | |  |
| NM_018360 | | | | CXorf15 | | | | | | | | | | | | | | -2.12 | | | | | | | 1.19E-02 | | | | | | 2.25 | 3.16E-02 | | | |  |
| NM_004762 | | | | CYTH1 | | | | | | | | | | | | | | -3.07 | | | | | | | 4.98E-04 | | | | | | 2.60 | 8.57E-03 | | | |  |
| NM_004227 | | | | CYTH3 | | | | | | | | | | | | | | -2.72 | | | | | | | 1.85E-03 | | | | | | 2.59 | 1.18E-02 | | | |  |
| NM_015330 | | | | CYTSA | | | | | | | | | | | | | | -3.29 | | | | | | | 3.89E-04 | | | | | | 2.97 | 2.53E-03 | | | |  |
| NM_001044723 | | | | CSNK1G3 | | | | | | | | | | | | | | -3.36 | | | | | | | 4.01E-04 | | | | | | 2.48 | 3.45E-03 | | | |  |
| NM_001077204 | | | | CSPP1 | | | | | | | | | | | | | | -4.45 | | | | | | | 7.69E-04 | | | | | | 3.90 | 5.31E-03 | | | |  |
| NM_032552 | | | | DAB2IP | | | | | | | | | | | | | | -3.81 | | | | | | | 2.83E-03 | | | | | | 2.61 | 2.13E-02 | | | |  |
| NM_003861 | | | DCAF5 | | | | | | | | | | -2.33 | | | | | | | | | | | | 1.42E-03 | | | | | | 2.46 | 6.53E-03 | | | |  |
| AK025344 | | | DCP1A | | | | | | | | | | -2.30 | | | | | | | | | | | | 3.29E-03 | | | | | | 2.33 | 5.24E-03 | | | |  |
| NM_004398 | | | DDX10 | | | | | | | | | | -4.75 | | | | | | | | | | | | 2.05E-04 | | | | | | 3.15 | 2.41E-03 | | | |  |
| NM_007242.5 | | | DDX19B | | | | | | | | | | -2.22 | | | | | | | | | | | | 5.36E-04 | | | | | | 2.27 | 3.47E-03 | | | |  |
| NM_022779 | | | DDX31 | | | | | | | | | | -2.30 | | | | | | | | | | | | 3.60E-03 | | | | | | 2.41 | 1.72E-02 | | | |  |
| NM_198459 | | | DENND2C | | | | | | | | | | -3.60 | | | | | | | | | | | | 1.57E-03 | | | | | | 3.63 | 1.04E-02 | | | |  |
| NM_015213 | | | DENND5A | | | | | | | | | | -2.83 | | | | | | | | | | | | 1.87E-02 | | | | | | 2.13 | ns. | | | |  |
| NM_014662 | | | DEPDC5 | | | | | | | | | | -5.13 | | | | | | | | | | | | 7.41E-04 | | | | | | 4.61 | 2.88E-03 | | | |  |
| NM_152879 | | | DGKD | | | | | | | | | | -2.89 | | | | | | | | | | | | 2.66E-03 | | | | | | 2.66 | 4.98E-03 | | | |  |
| NM_018180 | | | DHX32 | | | | | | | | | | -2.19 | | | | | | | | | | | | 2.91E-03 | | | | | | 2.32 | 3.79E-03 | | | |  |
| NM_021931 | | | DHX35 | | | | | | | | | | -2.10 | | | | | | | | | | | | 7.77E-04 | | | | | | 2.02 | 3.23E-03 | | | |  |
| NM_173602 | | | DIP2B | | | | | | | | | | -5.80 | | | | | | | | | | | | 5.10E-04 | | | | | | 4.35 | 3.20E-03 | | | |  |
| NM_152383 | | | DIS3L2 | | | | | | | | | | -2.23 | | | | | | | | | | | | 7.69E-04 | | | | | | 2.04 | 3.45E-03 | | | |  |
| AL831922 | | | DLG1 | | | | | | | | | | -3.10 | | | | | | | | | | | | 2.67E-04 | | | | | | 2.02 | 1.99E-03 | | | |  |
| AL833283 | | | DNMBP | | | | | | | | | | -3.39 | | | | | | | | | | | | 1.85E-03 | | | | | | 3.74 | 4.07E-03 | | | |  |
| NM_015375 | | | DSTYK | | | | | | | | | | -2.23 | | | | | | | | | | | | 4.03E-04 | | | | | | 2.43 | 2.88E-03 | | | |  |
| NM_183041 | | | DTNBP1 | | | | | | | | | | -3.51 | | | | | | | | | | | | 5.50E-04 | | | | | | 2.58 | 2.88E-03 | | | |  |
| NM_017803 | | | DUS2L | | | | | | | | | | -2.39 | | | | | | | | | | | | 7.03E-03 | | | | | | 2.38 | 1.76E-02 | | | |  |
| NM_130436 | | | DYRK1A | | | | | | | | | | -5.98 | | | | | | | | | | | | 4.74E-04 | | | | | | 5.16 | 3.45E-03 | | | |  |
| NM_003566 | | | EEA1 | | | | | | | | | | -4.66 | | | | | | | | | | | | 7.69E-04 | | | | | | 2.69 | 2.80E-03 | | | |  |
| NM_005228 | | | EGFR | | | | | | | | | | -2.80 | | | | | | | | | | | | 1.21E-03 | | | | | | 2.55 | 2.78E-03 | | | |  |
| NM_024757 | | | EHMT1 | | | | | | | | | | -3.62 | | | | | | | | | | | | 8.83E-04 | | | | | | 2.49 | 8.27E-03 | | | |  |
| NM_172373 | | | ELF1 | | | | | | | | | | -2.74 | | | | | | | | | | | | 1.27E-03 | | | | | | 2.72 | 7.30E-03 | | | |  |
| NM_005230 | | | ELK3 | | | | | | | | | | -2.11 | | | | | | | | | | | | 4.46E-03 | | | | | | 2.00 | 4.08E-02 | | | |  |
| NM_012081 | | | ELL2 | | | | | | | | | | -2.62 | | | | | | | | | | | | 1.78E-03 | | | | | | 2.25 | 1.48E-02 | | | |  |
| NM_020354 | | | ENTPD7 | | | | | | | | | | -2.39 | | | | | | | | | | | | 3.77E-03 | | | | | | 2.12 | 2.36E-02 | | | |  |
| NM_015630 | | | EPC2 | | | | | | | | | | -4.44 | | | | | | | | | | | | 4.98E-04 | | | | | | 3.59 | 4.09E-03 | | | |  |
| NM_014964 | | | EPN2 | | | | | | | | | | -2.87 | | | | | | | | | | | | 4.49E-03 | | | | | | 2.68 | 7.09E-03 | | | |  |
| NM_018695 | | | ERBB2IP | | | | | | | | | | -2.86 | | | | | | | | | | | | 4.09E-04 | | | | | | 2.19 | 4.26E-03 | | | |  |
| NM_020207 | | | ERCC6L2 | | | | | | | | | | -5.16 | | | | | | | | | | | | 6.44E-04 | | | | | | 4.06 | 6.06E-03 | | | |  |
| NM_207332 | | | ERICH1 | | | | | | | | | | -2.52 | | | | | | | | | | | | 6.33E-04 | | | | | | 2.21 | 6.36E-03 | | | |  |
| NM_052911 | | | ESCO1 | | | | | | | | | | -2.62 | | | | | | | | | | | | 4.09E-04 | | | | | | 2.34 | 3.79E-03 | | | |  |
| NM_001987 | | | | | | | ETV6 | | | | | | | | -3.67 | | | | | | | | | | 2.58E-04 | | | | | | 2.51 | 2.97E-03 | | | |  |
| NM_005665 | | | | | | | EVI5 | | | | | | | | -5.56 | | | | | | | | | | 4.60E-04 | | | | | | 3.64 | 3.23E-03 | | | |  |
| NM_000127 | | | | | | | EXT1 | | | | | | | | -5.70 | | | | | | | | | | 3.25E-04 | | | | | | 3.46 | 3.83E-03 | | | |  |
| NM_001440 | | | | | | | EXTL3 | | | | | | | | -2.43 | | | | | | | | | | 1.20E-03 | | | | | | 2.11 | 5.40E-03 | | | |  |
| NM_138348 | | | | | | | FAM105B | | | | | | | | -2.03 | | | | | | | | | | 4.02E-03 | | | | | | 2.18 | 1.25E-02 | | | |  |
| ENST00000258884 | | | | | | | FAM108C1 | | | | | | | | -3.84 | | | | | | | | | | 8.56E-03 | | | | | | 3.02 | 2.54E-02 | | | |  |
| NM_173511 | | | | | | | FAM117B | | | | | | | | -3.83 | | | | | | | | | | 3.25E-04 | | | | | | 2.45 | 3.48E-03 | | | |  |
| NM_173822 | | | | | | | FAM126B | | | | | | | | -3.24 | | | | | | | | | | 1.90E-03 | | | | | | 2.97 | 3.79E-03 | | | |  |
| NM_032181 | | | | | | | FAM176A | | | | | | | | -2.34 | | | | | | | | | | 2.23E-03 | | | | | | 2.02 | 1.41E-02 | | | |  |
| NM_018999 | | | | | | | FAM190B | | | | | | | | -6.42 | | | | | | | | | | 3.61E-04 | | | | | | 4.12 | 3.77E-03 | | | |  |
| NM_003704 | | | | | | | FAM193A | | | | | | | | -2.68 | | | | | | | | | | 3.32E-03 | | | | | | 3.49 | 5.81E-03 | | | |  |
| NM_001098801 | | | | | | | FAM210A | | | | | | | | -2.77 | | | | | | | | | | 1.39E-03 | | | | | | 2.66 | 4.25E-03 | | | |  |
| NM_019600 | | | | | | | FAM214A | | | | | | | | -2.87 | | | | | | | | | | 5.10E-04 | | | | | | 2.39 | 4.70E-03 | | | |  |
| NM_019054 | | | | | | | FAM35A | | | | | | | | -2.90 | | | | | | | | | | 4.74E-04 | | | | | | 2.30 | 4.34E-03 | | | |  |
| NM_014661 | | | | | | | FAM53B | | | | | | | | -2.90 | | | | | | | | | | 2.56E-03 | | | | | | 2.43 | 9.11E-03 | | | |  |
| NM_022751 | | | | | | | FAM59A | | | | | | | | -4.57 | | | | | | | | | | 2.67E-04 | | | | | | 3.86 | 3.42E-03 | | | |  |
| NM_021238 | | | | | | | FAM60A | | | | | | | | -2.77 | | | | | | | | | | 6.65E-04 | | | | | | 2.66 | 3.53E-03 | | | |  |
| NM_001040450 | | | | | | | FAM63B | | | | | | | | -2.42 | | | | | | | | | | 2.22E-02 | | | | | | 2.30 | 1.79E-02 | | | |  |
| AK024927 | | | | | | | FAM83B | | | | | | | | -3.41 | | | | | | | | | | 3.00E-03 | | | | | | 2.61 | 8.84E-03 | | | |  |
| NM_020937 | | | | | | | FANCM | | | | | | | | -2.48 | | | | | | | | | | 1.34E-02 | | | | | | 2.17 | ns. | | | |  |
| NM_005245 | | | | | | | FAT1 | | | | | | | | -2.67 | | | | | | | | | | 8.23E-03 | | | | | | 2.14 | 1.65E-02 | | | |  |
| NM_033535 | | | | | | | FBXL5 | | | | | | | | -2.24 | | | | | | | | | | 2.57E-04 | | | | | | 2.28 | 2.51E-03 | | | |  |
| NM_025133 | | | | | | | FBXO11 | | | | | | | | -3.74 | | | | | | | | | | 3.50E-04 | | | | | | 2.92 | 3.45E-03 | | | |  |
| NM_017943 | | | | | | | FBXO34 | | | | | | | | -3.69 | | | | | | | | | | 7.98E-04 | | | | | | 3.14 | 5.39E-03 | | | |  |
| NM_018994 | | | | | | | FBXO42 | | | | | | | | -2.90 | | | | | | | | | | 5.17E-04 | | | | | | 2.69 | 3.51E-03 | | | |  |
| NM_012300 | | | | | | | FBXW11 | | | | | | | | -4.52 | | | | | | | | | | 9.33E-04 | | | | | | 3.72 | 9.04E-03 | | | |  |
| NM_138782 | | | | | | | FCHO2 | | | | | | | | -4.09 | | | | | | | | | | 4.79E-04 | | | | | | 3.28 | 3.45E-03 | | | |  |
| NM_014824 | | | | | | | FCHSD2 | | | | | | | | -8.20 | | | | | | | | | | 4.32E-04 | | | | | | 4.38 | 6.91E-03 | | | |  |
| NM_139241 | | | | | | | FGD4 | | | | | | | | -4.37 | | | | | | | | | | 3.61E-04 | | | | | | 3.34 | 6.80E-03 | | | |  |
| NM_018351 | | | | | | | FGD6 | | | | | | | | -2.84 | | | | | | | | | | 2.92E-03 | | | | | | 2.39 | 2.57E-02 | | | |  |
| NM_002006 | | | | | | | FGF2 | | | | | | | | -2.70 | | | | | | | | | | 3.76E-03 | | | | | | 2.19 | 1.76E-02 | | | |  |
| NM_022970 | | | | | | | FGFR2 | | | | | | | | -4.76 | | | | | | | | | | 4.07E-04 | | | | | | 3.49 | 6.27E-03 | | | |  |
| NM_002017 | | | | | | | FLI1 | | | | | | | | -5.86 | | | | | | | | | | 1.43E-02 | | | | | | 3.42 | ns. | | | |  |
| AK123481 | | | | | | | FLJ41487 | | | | | | | -2.47 | | | | | | | | | | | | 2.81E-02 | | | | 3.16 | | | 3.53E-03 | | |  |
| NM_001024948 | | | | | | | FNBP1L | | | | | | | -3.58 | | | | | | | | | | | | 6.25E-04 | | | | 3.48 | | | 4.01E-03 | | |  |
| NM_022763 | | | | | | | FNDC3B | | | | | | | -19.10 | | | | | | | | | | | | 2.58E-04 | | | | 12.12 | | | 2.53E-03 | | |  |
| NM_014947 | | | | | | | FOXJ3 | | | | | | | -6.47 | | | | | | | | | | | | 5.03E-04 | | | | 5.52 | | | 3.81E-03 | | |  |
| NM_004514 | | | | | | | FOXK2 | | | | | | | -3.60 | | | | | | | | | | | | 7.79E-04 | | | | 3.20 | | | 4.07E-03 | | |  |
| NM_002015 | | | | | | | FOXO1 | | | | | | | -5.22 | | | | | | | | | | | | 1.67E-03 | | | | 3.63 | | | 1.61E-02 | | |  |
| NM_032682 | | | | | | | FOXP1 | | | | | | | -3.21 | | | | | | | | | | | | 4.55E-04 | | | | 2.26 | | | 5.64E-03 | | |  |
| NM_032892 | | | | | | | FRMD5 | | | | | | | -7.51 | | | | | | | | | | | | 4.00E-04 | | | | 5.41 | | | 6.43E-03 | | |  |
| NM_001042481 | | | | | | | FRMD6 | | | | | | | -2.89 | | | | | | | | | | | | 2.02E-03 | | | | 3.26 | | | 4.01E-03 | | |  |
| NM_015030 | | | | | | | FRYL | | | | | | | -3.54 | | | | | | | | | | | | 3.25E-04 | | | | 2.45 | | | 3.54E-03 | | |  |
| NM_032664 | | | | | | | FUT10 | | | | | | | -4.72 | | | | | | | | | | | | 7.20E-04 | | | | 4.17 | | | 4.43E-03 | | |  |
| NM_005254 | | | | | | | GABPB1 | | | | | | | -2.66 | | | | | | | | | | | | 2.21E-03 | | | | 2.13 | | | 3.06E-02 | | |  |
| AK024670 | | | | | | | GATAD2A | | | | | | | -2.16 | | | | | | | | | | | | 6.34E-03 | | | | 2.01 | | | 6.94E-03 | | |  |
| ENST00000258451 | | | | | | | GCC2 | | | | | | | -2.45 | | | | | | | | | | | | 4.94E-03 | | | | 2.41 | | | 1.48E-02 | | |  |
| NM_001498 | | | | | | | GCLC | | | | | | | -2.80 | | | | | | | | | | | | 1.74E-03 | | | | 2.37 | | | 5.00E-03 | | |  |
| NM_017686 | | | | | | | GDAP2 | | | | | | | -2.73 | | | | | | | | | | | | 1.46E-04 | | | | 2.64 | | | 1.58E-03 | | |  |
| NM_018988 | | | | | | | GFOD1 | | | | | | | -3.54 | | | | | | | | | | | | 1.88E-03 | | | | 3.70 | | | 6.00E-03 | | |  |
| NM_015575 | | | | | | | GIGYF2 | | | | | | | -7.82 | | | | | | | | | | | | 4.32E-04 | | | | 6.91 | | | 3.48E-03 | | |  |
| NM_138426 | | | | | | | GLCCI1 | | | | | | | -5.86 | | | | | | | | | | | | 5.84E-04 | | | | 4.08 | | | 4.07E-03 | | |  |
| NM_000168 | | | | | | | GLI3 | | | | | | | -8.70 | | | | | | | | | | | | 1.47E-03 | | | | 6.45 | | | 6.16E-03 | | |  |
| NM_014905 | | | | | | | GLS | | | | | | | -2.39 | | | | | | | | | | | | 1.18E-03 | | | | 2.20 | | | 3.43E-03 | | |  |
| NM_015349 | | | | | | | GLTSCR1L | | | | | | | -4.43 | | | | | | | | | | | | 3.25E-04 | | | | 3.44 | | | 2.07E-03 | | |  |
| NM_007353 | | | | | | | GNA12 | | | | | | | -2.71 | | | | | | | | | | | | 4.61E-04 | | | | 2.19 | | | 3.54E-03 | | |  |
| NM_002069 | | | | | | | GNAI1 | | | | | | | -2.65 | | | | | | | | | | | | 5.68E-04 | | | | 2.66 | | | 3.43E-03 | | |  |
| NM_053004 | | | | | | | GNB1L | | | | | | | -3.29 | | | | | | | | | | | | 2.23E-03 | | | | 2.29 | | | 5.63E-03 | | |  |
| NM_002078 | | | | | | | GOLGA4 | | | | | | | -3.42 | | | | | | | | | | | | 7.64E-04 | | | | 2.05 | | | 1.62E-02 | | |  |
| NM_022130 | | | | | | | GOLPH3 | | | | | | | -2.36 | | | | | | | | | | | | 5.46E-03 | | | | 2.02 | | | 2.09E-02 | | |  |
| NM_017786 | | | | | | | GOLSYN | | | | | | | -2.03 | | | | | | | | | | | | 7.08E-04 | | | | 2.08 | | | 3.79E-03 | | |  |
| NM_001037533 | | | | | | | GON4L | | | | | | | -2.17 | | | | | | | | | | | | 3.95E-03 | | | | 2.03 | | | 3.79E-03 | | |  |
| NM_018040 | | | | | | | GPATCH2 | | | | | | | -5.09 | | | | | | | | | | | | 3.82E-04 | | | | 3.46 | | | 3.59E-03 | | |  |
| NM_001002909 | | | | | | | GPATCH8 | | | | | | | -3.22 | | | | | | | | | | | | 1.91E-03 | | | | 3.14 | | | 5.15E-03 | | |  |
| NM_022913 | | | | | GPBP1 | | | | | | | | -2.96 | | | | | | | | | | | | | 9.60E-04 | | | | | 2.68 | 6.26E-03 | | | |  |
| NM_021639 | | | | | GPBP1L1 | | | | | | | | -2.83 | | | | | | | | | | | | | 5.10E-04 | | | | | 2.22 | 4.21E-03 | | | |  |
| NM_153840 | | | | | GPR110 | | | | | | | | -2.74 | | | | | | | | | | | | | 1.79E-03 | | | | | 2.42 | 4.73E-03 | | | |  |
| NM_145290 | | | | | GPR125 | | | | | | | | -2.55 | | | | | | | | | | | | | 4.60E-04 | | | | | 2.04 | 7.03E-03 | | | |  |
| AK122643 | | | | | GPR39 | | | | | | | | -6.18 | | | | | | | | | | | | | 3.25E-04 | | | | | 4.29 | 2.57E-03 | | | |  |
| NM_023927 | | | | | GRAMD3 | | | | | | | | -3.18 | | | | | | | | | | | | | 6.16E-03 | | | | | 2.86 | 2.03E-02 | | | |  |
| NM_024915 | | | | | GRHL2 | | | | | | | | -6.70 | | | | | | | | | | | | | 4.60E-04 | | | | | 4.18 | 4.56E-03 | | | |  |
| NM_005685 | | | | | GTF2IRD1 | | | | | | | | -4.16 | | | | | | | | | | | | | 3.83E-04 | | | | | 3.46 | 3.45E-03 | | | |  |
| NM_207331 | | | | | GUSPB1 | | | | | | | | -3.75 | | | | | | | | | | | | | 1.34E-03 | | | | | 2.28 | 3.00E-02 | | | |  |
| NM_053005 | | | | | HCCA2 | | | | | | | | -2.50 | | | | | | | | | | | | | 4.02E-03 | | | | | 2.29 | 1.02E-02 | | | |  |
| NM_012080 | | | | | HDHD1A | | | | | | | | -3.31 | | | | | | | | | | | | | 2.00E-03 | | | | | 2.68 | 7.89E-03 | | | |  |
| NM_019024 | | | | | HEATR5B | | | | | | | | -3.32 | | | | | | | | | | | | | 3.25E-04 | | | | | 2.86 | 2.60E-03 | | | |  |
| NM_001017972 | | | | | HERC4 | | | | | | | | -2.77 | | | | | | | | | | | | | 3.86E-04 | | | | | 2.22 | 3.11E-03 | | | |  |
| NM_003325 | | | | | HIRA | | | | | | | | -2.17 | | | | | | | | | | | | | 2.00E-02 | | | | | 2.25 | 2.64E-02 | | | |  |
| NM_005322 | | | | | HIST1H1B | | | | | | | | -4.88 | | | | | | | | | | | | | 7.43E-06 | | | | | 2.22 | 1.25E-02 | | | |  |
| NM_005320 | | | | | HIST1H1D | | | | | | | | -5.57 | | | | | | | | | | | | | 1.38E-05 | | | | | 2.13 | 3.03E-02 | | | |  |
| NM_002114 | | | | | HIVEP1 | | | | | | | | -6.87 | | | | | | | | | | | | | 1.78E-03 | | | | | 6.69 | 3.93E-03 | | | |  |
| NM_006734 | | | | | HIVEP2 | | | | | | | | -7.52 | | | | | | | | | | | | | 5.80E-04 | | | | | 6.73 | 4.04E-03 | | | |  |
| BX648950 | | | | | HMBOX1 | | | | | | | | -2.74 | | | | | | | | | | | | | 6.26E-03 | | | | | 2.24 | 8.60E-03 | | | |  |
| NM_018200 | | | | | HMG20A | | | | | | | | -2.97 | | | | | | | | | | | | | 6.25E-04 | | | | | 2.61 | 3.48E-03 | | | |  |
| NM_003483 | | | | | HMGA2 (J) | | | | | | | | -10.72 | | | | | | | | | | | | | 8.40E-04 | | | | | 8.46 | 4.43E-03 | | | |  |
| NM_000859 | | | | | HMGCR | | | | | | | | -2.15 | | | | | | | | | | | | | 2.11E-03 | | | | | 2.02 | 1.15E-02 | | | |  |
| NM_000861 | | | | | HRH1 | | | | | | | | -2.06 | | | | | | | | | | | | | 4.11E-03 | | | | | 2.13 | 2.12E-02 | | | |  |
| NM_005114 | | | | | HS3ST1 (J) | | | | | | | | -2.69 | | | | | | | | | | | | | 9.33E-04 | | | | | 2.23 | 9.17E-03 | | | |  |
| NM_019859 | | | | | HTR7 | | | | | | | | -3.93 | | | | | | | | | | | | | 9.09E-04 | | | | | 2.59 | 1.13E-02 | | | |  |
| NR_002774 | | | | | HTR7P | | | | | | | | -2.93 | | | | | | | | | | | | | 9.43E-04 | | | | | 2.21 | 5.68E-03 | | | |  |
| NM_001079526 | | | | | IKZF2 | | | | | | | | -6.46 | | | | | | | | | | | | | 5.05E-04 | | | | | 3.50 | 8.72E-03 | | | |  |
| NM_172174 | | | | | IL15 | | | | | | | | -2.66 | | | | | | | | | | | | | 3.66E-04 | | | | | 2.25 | 3.81E-03 | | | |  |
| NM_002182 | | | | | IL1RAP | | | | | | | | -5.10 | | | | | | | | | | | | | 5.10E-04 | | | | | 2.88 | 7.00E-03 | | | |  |
| NM_000418 | | | | | IL4R | | | | | | | | -2.36 | | | | | | | | | | | | | 5.58E-04 | | | | | 2.20 | 6.79E-03 | | | |  |
| NM_017553 | | | | | INO80 | | | | | | | | -3.03 | | | | | | | | | | | | | 3.61E-04 | | | | | 2.94 | 2.58E-03 | | | |  |
| NM_012141 | | INTS6 | | | | | | | | | | | -2.79 | | | | | | | | | | | | 4.54E-03 | | | | | | 2.48 | 8.19E-03 | | | |  |
| NM_015434 | | INTS7 | | | | | | | | | | | -2.44 | | | | | | | | | | | | 1.05E-03 | | | | | | 2.66 | 2.80E-03 | | | |  |
| NM_022755 | | IPPK | | | | | | | | | | | -2.41 | | | | | | | | | | | | 1.61E-02 | | | | | | 2.25 | 2.85E-02 | | | |  |
| NM_002199 | | IRF2 | | | | | | | | | | | -6.16 | | | | | | | | | | | | 5.97E-04 | | | | | | 4.37 | 6.94E-03 | | | |  |
| NM_031483 | | ITCH 🟋 | | | | | | | | | | | -3.59 | | | | | | | | | | | | 1.93E-03 | | | | | | 2.77 | 3.45E-03 | | | |  |
| NM_002203 | | ITGA2 (J) | | | | | | | | | | | -2.74 | | | | | | | | | | | | 8.68E-04 | | | | | | 2.37 | 9.76E-03 | | | |  |
| NM_002214 | | ITGB8 | | | | | | | | | | | -2.77 | | | | | | | | | | | | 9.83E-04 | | | | | | 2.32 | 3.60E-03 | | | |  |
| NM_002221 | | ITPKB | | | | | | | | | | | -3.61 | | | | | | | | | | | | 7.43E-04 | | | | | | 2.59 | 4.20E-03 | | | |  |
| NM_004973 | | JARID2 | | | | | | | | | | | -3.16 | | | | | | | | | | | | 5.10E-04 | | | | | | 2.55 | 4.29E-03 | | | |  |
| NM_032776 | | JMJD1C | | | | | | | | | | | -4.06 | | | | | | | | | | | | 1.69E-03 | | | | | | 3.29 | 4.07E-03 | | | |  |
| NM_015167 | | JMJD6 | | | | | | | | | | | -2.36 | | | | | | | | | | | | 3.31E-03 | | | | | | 2.28 | 7.00E-03 | | | |  |
| NM_152405 | | JMY | | | | | | | | | | | -2.53 | | | | | | | | | | | | 8.62E-04 | | | | | | 2.50 | 3.45E-03 | | | |  |
| NM_015443 | | KANSL1 | | | | | | | | | | | -4.76 | | | | | | | | | | | | 4.32E-04 | | | | | | 4.02 | 3.54E-03 | | | |  |
| NM_032116 | | KATNAL1 | | | | | | | | | | | -4.33 | | | | | | | | | | | | 8.60E-04 | | | | | | 4.01 | 5.51E-03 | | | |  |
| NM_020122 | | KCMF1 | | | | | | | | | | | -4.87 | | | | | | | | | | | | 4.55E-04 | | | | | | 4.54 | 3.45E-03 | | | |  |
| NM_006618 | | KDM5B | | | | | | | | | | | -2.57 | | | | | | | | | | | | 4.09E-04 | | | | | | 2.14 | 3.11E-03 | | | |  |
| NM_021140 | | KDM6A | | | | | | | | | | | -6.13 | | | | | | | | | | | | 4.85E-04 | | | | | | 4.28 | 4.80E-03 | | | |  |
| NM_014743 | | KIAA0232 | | | | | | | | | | | -2.60 | | | | | | | | | | | | 1.81E-03 | | | | | | 2.32 | 4.34E-03 | | | |  |
| NM_014734 | | KIAA0247 | | | | | | | | | | | -3.69 | | | | | | | | | | | | 5.10E-04 | | | | | | 2.97 | 3.81E-03 | | | |  |
| NM_014686 | | KIAA0355 | | | | | | | | | | | -2.94 | | | | | | | | | | | | 1.94E-03 | | | | | | 2.53 | 7.35E-03 | | | |  |
| AB023164 | | KIAA0947 | | | | | | | | | | | -2.25 | | | | | | | | | | | | 8.35E-04 | | | | | | 2.02 | 5.96E-03 | | | |  |
| AB037853 | | KIAA1432 | | | | | | | | | | | -4.95 | | | | | | | | | | | | 4.74E-04 | | | | | | 3.51 | 4.43E-03 | | | |  |
| AL136736 | | KIAA1549 | | | | | | | | | | | -3.96 | | | | | | | | | | | | 1.70E-03 | | | | | | 3.34 | 6.89E-03 | | | |  |
| NM_004523 | | KIF11 | | | | | | | | | | | -3.02 | | | | | | | | | | | | 4.27E-04 | | | | | | 2.15 | 5.48E-03 | | | |  |
| NM_022113 | | KIF13A | | | | | | | | | | | -2.47 | | | | | | | | | | | | 7.86E-03 | | | | | | 2.07 | 3.04E-02 | | | |  |
| NM_015074 | | KIF1B | | | | | | | | | | | -4.21 | | | | | | | | | | | | 4.55E-04 | | | | | | 2.88 | 2.88E-03 | | | |  |
| NM_016195 | | KIF20B | | | | | | | | | | | -3.43 | | | | | | | | | | | | 1.31E-02 | | | | | | 2.24 | 3.19E-02 | | | |  |
| NM_004520 | | KIF2A | | | | | | | | | | | -3.76 | | | | | | | | | | | | 4.52E-04 | | | | | | 3.28 | 3.79E-03 | | | |  |
| NM_018240 | | KIRREL | | | | | | | | | | | -2.79 | | | | | | | | | | | | 1.26E-03 | | | | | | 2.01 | 1.49E-02 | | | |  |
| NM_000899 | | KITLG | | | | | | | | | | | -3.53 | | | | | | | | | | | | 5.02E-04 | | | | | | 2.58 | 6.49E-03 | | | |  |
| NM_003709 | | KLF7 (J) | | | | | | | | | | | -3.52 | | | | | | | | | | | | 3.25E-04 | | | | | | 3.12 | 3.79E-03 | | | |  |
| NM_014997 | | | | | KLHDC10 | | | | | | | | | -2.46 | | | | | | | | | | | | 6.14E-04 | | | | 2.24 | | | 4.24E-03 | | |  |
| NM_007246 | | | | | KLHL2 | | | | | | | | | -2.93 | | | | | | | | | | | | 9.43E-04 | | | | 2.50 | | | 8.84E-03 | | |  |
| NM_014458 | | | | | KLHL20 | | | | | | | | | -2.42 | | | | | | | | | | | | 8.60E-04 | | | | 2.24 | | | 4.26E-03 | | |  |
| NM_032438 | | | | | L3MBTL3 | | | | | | | | | -4.27 | | | | | | | | | | | | 5.82E-03 | | | | 3.39 | | | 2.57E-02 | | |  |
| NM_199188 | | | | | LARP4 | | | | | | | | | -2.18 | | | | | | | | | | | | 1.83E-03 | | | | 2.16 | | | 1.52E-02 | | |  |
| NM_015155 | | | | | LARP4B | | | | | | | | | -3.35 | | | | | | | | | | | | 7.10E-04 | | | | 2.96 | | | 1.99E-03 | | |  |
| NM_032440 | | | | | LCOR | | | | | | | | | -2.66 | | | | | | | | | | | | 6.13E-04 | | | | 2.39 | | | 5.21E-03 | | |  |
| NM_000527 | | | | | LDLR | | | | | | | | | -2.50 | | | | | | | | | | | | 5.21E-03 | | | | 2.35 | | | 2.26E-02 | | |  |
| NM_014319 | | | | | LEMD3 | | | | | | | | | -2.36 | | | | | | | | | | | | 4.61E-04 | | | | 2.36 | | | 3.54E-03 | | |  |
| NM_018490 | | | | | LGR4 | | | | | | | | | -4.48 | | | | | | | | | | | | 4.61E-04 | | | | 3.51 | | | 3.55E-03 | | |  |
| NM_005779 | | | | | LHFPL2 | | | | | | | | | -7.74 | | | | | | | | | | | | 1.19E-03 | | | | 7.51 | | | 4.54E-03 | | |  |
| NM_033343 | | | | | LHX4 | | | | | | | | | -6.35 | | | | | | | | | | | | 4.39E-04 | | | | 4.30 | | | 3.79E-03 | | |  |
| NM_173083 | | | | | LIN9 | | | | | | | | | -2.53 | | | | | | | | | | | | 9.43E-04 | | | | 2.02 | | | 7.80E-03 | | |  |
| NM_014916 | | | | | LMTK2 | | | | | | | | | -2.31 | | | | | | | | | | | | 8.28E-03 | | | | 2.28 | | | 2.27E-02 | | |  |
| NM_058169 | | | | | LOH12CR1 | | | | | | | | | -3.54 | | | | | | | | | | | | 3.25E-04 | | | | 2.64 | | | 2.41E-03 | | |  |
| NM_057159 | | | | | LPAR1 | | | | | | | | | -5.19 | | | | | | | | | | | | 2.59E-03 | | | | 3.94 | | | 1.80E-02 | | |  |
| NM_024830 | | | | | LPCAT1 | | | | | | | | | -2.38 | | | | | | | | | | | | 8.17E-03 | | | | 2.50 | | | 2.25E-02 | | |  |
| NM_145693 | | | | | LPIN1 | | | | | | | | | -2.01 | | | | | | | | | | | | 2.16E-03 | | | | 2.18 | | | 1.68E-02 | | |  |
| NM_001164211 | | | | | LRCH1 | | | | | | | | | -15.45 | | | | | | | | | | | | 1.29E-03 | | | | 10.64 | | | 7.93E-03 | | |  |
| NM_002336 | | | | | LRP6 | | | | | | | | | -5.02 | | | | | | | | | | | | 5.80E-04 | | | | 3.18 | | | 5.77E-03 | | |  |
| NM_033300 | | | | | LRP8 (J) | | | | | | | | | -2.96 | | | | | | | | | | | | 8.41E-04 | | | | 2.35 | | | 8.57E-03 | | |  |
| NM_018214 | | | | | LRRC1 | | | | | | | | | -4.71 | | | | | | | | | | | | 1.29E-03 | | | | 2.92 | | | 1.83E-02 | | |  |
| NM_032270 | | | | | LRRC8C | | | | | | | | | -2.86 | | | | | | | | | | | | 2.87E-03 | | | | 3.03 | | | 1.72E-02 | | |  |
| NM_033631 | | | | | LUZP1 | | | | | | | | | -2.26 | | | | | | | | | | | | 1.39E-02 | | | | 2.43 | | | 5.77E-03 | | |  |
| NM_002350 | | | | | LYN | | | | | | | | | -2.52 | | | | | | | | | | | | 6.24E-04 | | | | 2.15 | | | 4.34E-03 | | |  |
| NM_000081 | | | | | LYST | | | | | | | | | -5.40 | | | | | | | | | | | | 3.51E-04 | | | | 4.40 | | | 3.11E-03 | | |  |
| NM_182762 | | | | | MACC1 | | | | | | | | | -4.33 | | | | | | | | | | | | 1.03E-03 | | | | 3.24 | | | 8.56E-03 | | |  |
| NM_003010 | | | | | MAP2K4 | | | | | | | | | -4.98 | | | | | | | | | | | | 1.13E-03 | | | | 3.95 | | | 8.03E-03 | | |  |
| NM_005923 | | | | | MAP3K5 | | | | | | | | | -6.25 | | | | | | | | | | | | 2.70E-04 | | | | 3.08 | | | 1.28E-02 | | |  |
| NM_033141 | | | | | MAP3K9 | | | | | | | | | -2.13 | | | | | | | | | | | | 4.39E-03 | | | | 2.17 | | | 6.99E-03 | | |  |
| NM_001315 | | | | | MAPK14 | | | | | | | | | -3.05 | | | | | | | | | | | | 1.46E-04 | | | | 2.55 | | | 1.58E-03 | | |  |
| NM_005885 | | | | | | | MARCH6 | | | | | | | | | -2.41 | | | | | | | | | 9.83E-04 | | | | | | 2.14 | 6.74E-03 | | | |  |
| NM_018650 | | | | | | | MARK1 | | | | | | | | | -5.02 | | | | | | | | | 1.23E-03 | | | | | | 3.90 | 7.11E-03 | | | |  |
| NM_020690 | | | | | | | MASK-BP3 | | | | | | | | | -2.34 | | | | | | | | | 3.68E-04 | | | | | | 2.07 | 2.51E-03 | | | |  |
| ENST00000261569 | | | | | | | MAST4 | | | | | | | | | -3.11 | | | | | | | | | 9.33E-04 | | | | | | 3.09 | 6.98E-03 | | | |  |
| NM_145113 | | | | | | | MAX | | | | | | | | | -2.24 | | | | | | | | | 4.63E-03 | | | | | | 2.27 | 8.64E-03 | | | |  |
| NM_144778 | | | | | | | MBNL2 | | | | | | | | | -3.61 | | | | | | | | | 2.50E-03 | | | | | | 2.38 | 1.13E-02 | | | |  |
| NM_001080480 | | | | | | | MBOAT1 | | | | | | | | | -3.22 | | | | | | | | | 3.51E-04 | | | | | | 2.30 | 2.41E-03 | | | |  |
| NM_001025100 | | | | | | | MBP | | | | | | | | | -3.62 | | | | | | | | | 5.54E-04 | | | | | | 2.69 | 4.09E-03 | | | |  |
| EU076749 | | | | | | | MDM2 🟋 | | | | | | | | | -2.39 | | | | | | | | | 7.66E-04 | | | | | | 2.13 | 5.21E-03 | | | |  |
| NM_005121 | | | | | | | MED13 | | | | | | | | | -3.28 | | | | | | | | | 5.63E-04 | | | | | | 2.86 | 3.45E-03 | | | |  |
| NM_015335 | | | | | | | MED13L | | | | | | | | | -6.79 | | | | | | | | | 9.31E-04 | | | | | | 4.53 | 3.14E-03 | | | |  |
| NM_170677 | | | | | | | MEIS2 | | | | | | | | | -5.02 | | | | | | | | | 7.26E-04 | | | | | | 3.47 | 3.43E-03 | | | |  |
| NM_000245 | | | | | | | MET | | | | | | | | | -2.64 | | | | | | | | | 1.45E-03 | | | | | | 2.17 | 2.57E-02 | | | |  |
| NM_006838 | | | | | | | METAP2 | | | | | | | | | -2.21 | | | | | | | | | 7.13E-04 | | | | | | 2.25 | 3.11E-03 | | | |  |
| NM_152636 | | | | | | | METT5D1 | | | | | | | | | -3.50 | | | | | | | | | 3.68E-04 | | | | | | 2.21 | 2.82E-03 | | | |  |
| NM_024770 | | | | | | | METTL8 | | | | | | | | | -2.39 | | | | | | | | | 5.47E-04 | | | | | | 2.04 | 5.86E-03 | | | |  |
| NM_001164273 | | | | | | | MGA | | | | | | | | | -4.66 | | | | | | | | | 4.74E-04 | | | | | | 3.78 | 2.88E-03 | | | |  |
| NR_003682 | | | | | | | MGC70870 | | | | | | | | | -2.76 | | | | | | | | | 5.08E-04 | | | | | | 2.41 | 3.45E-03 | | | |  |
| NM_014632 | | | | | | | MICAL2 | | | | | | | | | -3.04 | | | | | | | | | 3.61E-04 | | | | | | 2.69 | 7.69E-03 | | | |  |
| NM_015241 | | | | | | | MICAL3 | | | | | | | | | -3.26 | | | | | | | | | 5.57E-03 | | | | | | 2.90 | 3.59E-03 | | | |  |
| NM_033290 | | | | | | | MID1 | | | | | | | | | -3.19 | | | | | | | | | 2.43E-03 | | | | | | 2.50 | 6.94E-03 | | | |  |
| NM_012216 | | | | | | | MID2 | | | | | | | | | -2.81 | | | | | | | | | 5.10E-04 | | | | | | 2.42 | 6.89E-03 | | | |  |
| NM_198159 | | | | | | | MITF | | | | | | | | | -4.22 | | | | | | | | | 3.24E-03 | | | | | | 3.67 | 8.20E-03 | | | |  |
| NM_020831 | | | | | | | MKL1 | | | | | | | | | -6.80 | | | | | | | | | 6.85E-04 | | | | | | 6.42 | 3.54E-03 | | | |  |
| NM_014048 | | | | | | | MKL2 | | | | | | | | | -9.11 | | | | | | | | | 3.51E-04 | | | | | | 5.64 | 2.57E-03 | | | |  |
| NM_013255 | | | | | | | MKLN1 | | | | | | | | | -4.74 | | | | | | | | | 4.60E-04 | | | | | | 3.12 | 4.20E-03 | | | |  |
| NM_182931 | | | | | | | MLL5 | | | | | | | | | -3.04 | | | | | | | | | 1.55E-03 | | | | | | 3.10 | 5.15E-03 | | | |  |
| NM_001009569 | | | | | | | MLLT10 | | | | | | | | | -3.36 | | | | | | | | | 4.09E-04 | | | | | | 3.15 | 5.83E-03 | | | |  |
| NM_173468 | | | | | | | MOBKL1A | | | | | | | | | -2.87 | | | | | | | | | 1.10E-03 | | | | | | 2.03 | 2.00E-02 | | | |  |
| NM_024761 | | | | | | | MOBKL2B | | | | | | | | | -4.62 | | | | | | | | | 1.34E-03 | | | | | | 4.22 | 5.71E-03 | | | |  |
| NM_022474 | | | | | | | MPP5 | | | | | | | | | -2.32 | | | | | | | | | 7.68E-03 | | | | | | 2.17 | 1.80E-02 | | | |  |
| NM_002451 | | | | | MTAP | | | | -2.89 | | | | | | | | | | | | | | | | | 1.40E-03 | | | | | 2.77 | 4.29E-03 | | | |  |
| NM_007358 | | | | | MTF2 | | | | -2.02 | | | | | | | | | | | | | | | | | 5.79E-03 | | | | | 2.15 | 4.07E-03 | | | |  |
| NM_000252 | | | | | MTM1 | | | | -3.18 | | | | | | | | | | | | | | | | | 4.98E-04 | | | | | 2.71 | 5.43E-03 | | | |  |
| NM_021090 | | | | | MTMR3 | | | | -3.63 | | | | | | | | | | | | | | | | | 4.03E-04 | | | | | 3.22 | 4.70E-03 | | | |  |
| NM_012334 | | | | | MYO10 | | | | -2.98 | | | | | | | | | | | | | | | | | 3.07E-03 | | | | | 2.35 | 7.83E-03 | | | |  |
| NM_004998 | | | | | MYO1E | | | | -3.48 | | | | | | | | | | | | | | | | | 7.41E-04 | | | | | 2.31 | 8.34E-03 | | | |  |
| NM_006766 | | | | | MYST3 | | | | -3.19 | | | | | | | | | | | | | | | | | 7.78E-03 | | | | | 2.79 | 6.99E-03 | | | |  |
| NM_153029 | | | | | N4BP1 | | | | -3.08 | | | | | | | | | | | | | | | | | 8.62E-04 | | | | | 2.63 | 4.09E-03 | | | |  |
| NM_182964 | | | | | NAV2 | | | | -4.09 | | | | | | | | | | | | | | | | | 2.64E-02 | | | | | 2.09 | ns. | | | |  |
| NM_014903 | | | | | NAV3 | | | | -13.33 | | | | | | | | | | | | | | | | | 1.32E-03 | | | | | 9.85 | 5.15E-03 | | | |  |
| NM_006153 | | | | | NCK1 | | | | -2.51 | | | | | | | | | | | | | | | | | 1.42E-03 | | | | | 2.20 | 3.77E-03 | | | |  |
| NM_181659 | | | | | NCOA3 | | | | -4.94 | | | | | | | | | | | | | | | | | 6.66E-04 | | | | | 3.84 | 3.45E-03 | | | |  |
| NM_014071 | | | | | NCOA6 | | | | -3.18 | | | | | | | | | | | | | | | | | 4.60E-03 | | | | | 3.02 | 5.41E-03 | | | |  |
| NM_198400 | | | | | NEDD4 | | | | -2.92 | | | | | | | | | | | | | | | | | 1.50E-03 | | | | | 2.16 | 4.01E-03 | | | |  |
| NM_015277 | | | | | NEDD4L | | | | -4.41 | | | | | | | | | | | | | | | | | 4.45E-04 | | | | | 2.57 | 6.21E-03 | | | |  |
| NM_014397 | | | | | NEK6 | | | | -3.50 | | | | | | | | | | | | | | | | | 1.55E-03 | | | | | 2.84 | 4.80E-03 | | | |  |
| NM_001042492 | | | | | NF1 | | | | -5.13 | | | | | | | | | | | | | | | | | 2.67E-04 | | | | | 2.76 | 3.14E-03 | | | |  |
| NM_138714 | | | | | NFAT5 | | | | -4.52 | | | | | | | | | | | | | | | | | 1.68E-03 | | | | | 3.68 | 4.70E-03 | | | |  |
| NM_173164 | | | | | NFATC3 | | | | -2.92 | | | | | | | | | | | | | | | | | 5.48E-04 | | | | | 2.43 | 3.17E-03 | | | |  |
| NM_198514 | | | | | NHLRC2 | | | | -2.16 | | | | | | | | | | | | | | | | | 5.61E-03 | | | | | 2.07 | 1.30E-02 | | | |  |
| NM_020921 | | | | | NIN | | | | -3.25 | | | | | | | | | | | | | | | | | 8.62E-04 | | | | | 2.18 | ns. | | | |  |
| NM_133433 | | | | | NIPBL | | | | -3.75 | | | | | | | | | | | | | | | | | 1.09E-03 | | | | | 2.97 | 1.48E-02 | | | |  |
| NM_001018077 | | | | | NR3C1 | | | | -4.49 | | | | | | | | | | | | | | | | | 4.98E-04 | | | | | 3.67 | 3.45E-03 | | | |  |
| NM_013960 | | | | | NRG1 | | | | -4.49 | | | | | | | | | | | | | | | | | 2.58E-04 | | | | | 2.69 | 3.83E-02 | | | |  |
| NM_003489 | | | | | NRIP1 | | | | -2.83 | | | | | | | | | | | | | | | | | 5.05E-03 | | | | | 2.64 | 9.04E-03 | | | |  |
| NM_003873 | | | | | NRP1 | | | | -4.84 | | | | | | | | | | | | | | | | | 1.09E-03 | | | | | 3.08 | ns. | | | |  |
| NM_201266 | | | | | NRP2 | | | | -3.46 | | | | | | | | | | | | | | | | | 7.04E-04 | | | | | 2.45 | 6.58E-03 | | | |  |
| NM_022455 | | | | | NSD1 | | | | -3.21 | | | | | | | | | | | | | | | | | 7.50E-04 | | | | | 2.85 | 3.38E-03 | | | |  |
| NM_014840 | | | | | NUAK1 | | | | -3.28 | | | | | | | | | | | | | | | | | 1.27E-03 | | | | | 3.40 | 6.14E-03 | | | |  |
| NM_005124 | | | | | NUP153 | | | | -4.19 | | | | | | | | | | | | | | | | | 5.57E-04 | | | | | 3.72 | 3.54E-03 | | | |  |
| NM_138285 | | | | | NUP35 | | | | -3.64 | | | | | | | | | | | | | | | | | 4.03E-04 | | | | | 2.83 | 3.36E-03 | | | |  |
| NM_002538 | | | | | OCLN (J) | | | | -2.53 | | | | | | | | | | | | | | | | | 3.69E-03 | | | | | 2.09 | 2.14E-02 | | | |  |
| NM_006190 | | | | | ORC2L | | | | -2.49 | | | | | | | | | | | | | | | | | 1.44E-03 | | | | | 2.49 | 5.90E-03 | | | |  |
| NM_181747 | | | | | ORC5L | | | | -2.23 | | | | | | | | | | | | | | | | | 3.31E-03 | | | | | 2.32 | 4.13E-03 | | | |  |
| NM_017784 | | | | | OSBPL10 | | | | | | | | | | | -3.91 | | | | | | | | | 1.59E-03 | | | | | | 2.40 | 4.40E-02 | | | |  |
| NM_021220 | | | | | OVOL2 | | | | | | | | | | | -2.19 | | | | | | | | | 3.51E-04 | | | | | | 2.51 | 2.57E-03 | | | |  |
| AK096148 | | | | | OXR1 | | | | | | | | | | | -2.41 | | | | | | | | | 2.98E-03 | | | | | | 2.50 | 7.13E-03 | | | |  |
| NM_005109 | | | | | OXSR1 | | | | | | | | | | | -2.32 | | | | | | | | | 6.73E-04 | | | | | | 2.06 | 1.00E-02 | | | |  |
| NM_001184970 | | | | | PACSIN2 | | | | | | | | | | | -3.77 | | | | | | | | | 4.55E-04 | | | | | | 2.29 | 7.55E-03 | | | |  |
| NM_000430 | | | | | PAFAH1B1 | | | | | | | | | | | -3.58 | | | | | | | | | 9.95E-04 | | | | | | 2.73 | 6.87E-03 | | | |  |
| NM_018440 | | | | | PAG1 | | | | | | | | | | | -3.04 | | | | | | | | | 1.83E-02 | | | | | | 2.80 | 1.72E-02 | | | |  |
| NM_006451 | | | | | PAIP1 | | | | | | | | | | | -2.75 | | | | | | | | | 5.25E-04 | | | | | | 2.41 | 5.37E-03 | | | |  |
| NM_175854 | | | | | PAN3 | | | | | | | | | | | -4.14 | | | | | | | | | 6.62E-04 | | | | | | 2.95 | 2.57E-03 | | | |  |
| NM_015368 | | | | | PANX1 | | | | | | | | | | | -2.38 | | | | | | | | | 2.52E-03 | | | | | | 2.41 | 4.42E-03 | | | |  |
| NM_024615 | | | | | PARP8 | | | | | | | | | | | -3.26 | | | | | | | | | 1.15E-03 | | | | | | 2.90 | 3.48E-03 | | | |  |
| NM_006315 | | | | | PCGF3 | | | | | | | | | | | -2.23 | | | | | | | | | 1.03E-03 | | | | | | 2.01 | 4.80E-03 | | | |  |
| NM_002603 | | | | | PDE7A | | | | | | | | | | | -3.93 | | | | | | | | | 2.33E-03 | | | | | | 2.84 | 5.55E-03 | | | |  |
| NM_015032 | | | | | PDS5B | | | | | | | | | | | -4.16 | | | | | | | | | 3.51E-04 | | | | | | 3.01 | 3.79E-03 | | | |  |
| NM_004565 | | | | | PEX14 | | | | | | | | | | | -4.10 | | | | | | | | | 4.09E-04 | | | | | | 2.77 | 3.45E-03 | | | |  |
| NM_014721 | | | | | PHACTR2 | | | | | | | | | | | -4.99 | | | | | | | | | 5.57E-04 | | | | | | 2.72 | ns. | | | |  |
| NM_001048183 | | | | | PHACTR4 | | | | | | | | | | | -3.05 | | | | | | | | | 8.60E-04 | | | | | | 2.49 | 4.98E-03 | | | |  |
| NM_024947 | | | | | PHC3 | | | | | | | | | | | -2.16 | | | | | | | | | 5.13E-04 | | | | | | 2.27 | 3.00E-03 | | | |  |
| NM_015288 | | | | | PHF15 | | | | | | | | | | | -2.86 | | | | | | | | | 3.62E-03 | | | | | | 2.80 | 5.62E-03 | | | |  |
| NM_005392 | | | | | PHF2 | | | | | | | | | | | -2.77 | | | | | | | | | 7.45E-04 | | | | | | 2.04 | 3.79E-03 | | | |  |
| NM_016436 | | | | | PHF20 | | | | | | | | | | | -4.78 | | | | | | | | | 1.40E-03 | | | | | | 3.79 | 5.31E-03 | | | |  |
| NM_015153 | | | | | PHF3 | | | | | | | | | | | -2.74 | | | | | | | | | 1.18E-02 | | | | | | 2.17 | ns. | | | |  |
| NM_015107 | | | | | PHF8 | | | | | | | | | | | -2.50 | | | | | | | | | 1.63E-02 | | | | | | 2.10 | ns. | | | |  |
| NM_145753 | | | | | PHLDB2 | | | | | | | | | | | -3.03 | | | | | | | | | 1.21E-02 | | | | | | 2.01 | 4.48E-02 | | | |  |
| NM_015020 | | | | | PHLPP2 | | | | | | | | | | | -2.20 | | | | | | | | | 1.73E-02 | | | | | | 2.77 | 6.06E-03 | | | |  |
| NM_016166 | | | | | PIAS1 | | | | | | | | | | | -4.32 | | | | | | | | | 5.23E-04 | | | | | | 3.51 | 4.55E-03 | | | |  |
| NM_002647 | | | | | PIK3C3 | | | | | | | | | | | -2.95 | | | | | | | | | 2.27E-03 | | | | | | 2.13 | 1.69E-02 | | | |  |
| NM_181523 | | | | | PIK3R1 | | | | | | | | | | | -3.21 | | | | | | | | | 6.26E-04 | | | | | | 2.56 | ns. | | | |  |
| NM_015040 | | | | | PIKFYVE | | | | | | | | | | | -3.23 | | | | | | | | | 1.46E-03 | | | | | | 2.76 | 9.82E-03 | | | |  |
| NM_017884 | | | | | PINX1 | | | | | | | | | | | -2.10 | | | | | | | | | 9.11E-04 | | | | | | 2.26 | 4.62E-03 | | | |  |
| NM_005028 | | | | | PIP4K2A | | | | | | | | | | | -2.79 | | | | | | | | | 5.39E-04 | | | | | | 2.09 | 1.75E-02 | | | |  |
| NM_012399 | | | | | PITPNB | | | | | | | | | | | -2.40 | | | | | | | | | 4.10E-04 | | | | | | 2.05 | 3.54E-03 | | | |  |
| NM_020845 | | | | | PITPNM2 | | | | | | | | | | | -6.19 | | | | | | | | | 1.05E-03 | | | | | | 4.49 | 3.79E-03 | | | |  |
| NM_004571 | | | | | PKNOX1 | | | | | | | | | | | -2.50 | | | | | | | | | 5.57E-04 | | | | | | 2.35 | 2.80E-03 | | | |  |
| NM_015549 | | | | | PLEKHG3 | | | | | | | | | | | -2.42 | | | | | | | | | 1.49E-02 | | | | | | 2.83 | 3.64E-02 | | | |  |
| NM_000303 | | | | | | | PMM2 | | | | | | | | | -3.20 | | | | | | | | | 2.32E-03 | | | | | | 3.26 | 4.66E-03 | | | |  |
| NM_199420 | | | | | | | POLQ | | | | | | | | | -2.56 | | | | | | | | | 4.55E-04 | | | | | | 2.39 | 5.59E-03 | | | |  |
| NM_018082 | | | | | | | POLR3B | | | | | | | | | -4.48 | | | | | | | | | 3.51E-04 | | | | | | 3.55 | 2.88E-03 | | | |  |
| NM_002697 | | | | | | | POU2F1 | | | | | | | | | -3.45 | | | | | | | | | 2.58E-04 | | | | | | 2.20 | 3.87E-03 | | | |  |
| NM_006238 | | | | | | | PPARD | | | | | | | | | -2.45 | | | | | | | | | 2.39E-03 | | | | | | 2.06 | 5.59E-03 | | | |  |
| NM_138711 | | | | | | | PPARG | | | | | | | | | -3.89 | | | | | | | | | 3.50E-04 | | | | | | 2.57 | 3.54E-03 | | | |  |
| NM_133263 | | | | | | | PPARGC1B | | | | | | | | | -3.84 | | | | | | | | | 1.56E-03 | | | | | | 3.95 | 3.36E-03 | | | |  |
| NM_003626 | | | | | | | PPFIA1 | | | | | | | | | -4.12 | | | | | | | | | 9.94E-04 | | | | | | 3.66 | 5.32E-03 | | | |  |
| NM_177968 | | | | | | | PPM1B (J) | | | | | | | | | -2.91 | | | | | | | | | 3.63E-03 | | | | | | 2.38 | 7.41E-03 | | | |  |
| NM_002480 | | | | | | | PPP1R12A | | | | | | | | | -3.25 | | | | | | | | | 1.46E-03 | | | | | | 2.45 | 9.22E-03 | | | |  |
| NM_032105 | | | | | | | PPP1R12B | | | | | | | | | -2.48 | | | | | | | | | 3.76E-03 | | | | | | 2.05 | 8.00E-03 | | | |  |
| NM_018461 | | | | | | | PPP2R2D | | | | | | | | | -2.24 | | | | | | | | | 2.05E-03 | | | | | | 2.19 | 9.86E-03 | | | |  |
| NM_002718 | | | | | | | PPP2R3A | | | | | | | | | -3.49 | | | | | | | | | 2.26E-03 | | | | | | 2.37 | 6.94E-03 | | | |  |
| NM_005605 | | | | | | | PPP3CC | | | | | | | | | -3.11 | | | | | | | | | 6.25E-04 | | | | | | 2.62 | 5.21E-03 | | | |  |
| NM_001042388 | | | | | | | PPP4R1 | | | | | | | | | -2.29 | | | | | | | | | 3.01E-03 | | | | | | 2.06 | 8.98E-03 | | | |  |
| NM_199437 | | | | | | | PRDM10 | | | | | | | | | -2.77 | | | | | | | | | 4.32E-04 | | | | | | 2.35 | 3.14E-03 | | | |  |
| NM_006255 | | | | | | | PRKCH | | | | | | | | | -3.85 | | | | | | | | | 6.90E-04 | | | | | | 2.26 | 1.78E-02 | | | |  |
| NM_020719 | | | | | | | PRR12 | | | | | | | | | -2.54 | | | | | | | | | 1.79E-02 | | | | | | 3.11 | 3.60E-03 | | | |  |
| NM_000950 | | | | | | | PRRG1 | | | | | | | | | -4.20 | | | | | | | | | 2.43E-03 | | | | | | 3.23 | 6.79E-03 | | | |  |
| NM_173174 | | | | | | | PTK2B (J) | | | | | | | | | -2.96 | | | | | | | | | 7.84E-03 | | | | | | 2.52 | 3.60E-03 | | | |  |
| NM_002827 | | | | | | | PTPN1 | | | | | | | | | -2.15 | | | | | | | | | 2.59E-03 | | | | | | 2.09 | 8.26E-03 | | | |  |
| NM_005401 | | | | | | | PTPN14 | | | | | | | | | -2.68 | | | | | | | | | 1.27E-03 | | | | | | 2.39 | 1.93E-02 | | | |  |
| NM_007039 | | | | | | | PTPN21 | | | | | | | | | -2.18 | | | | | | | | | 4.96E-03 | | | | | | 2.37 | 2.07E-02 | | | |  |
| NM_002829 | | | | | | | PTPN3 | | | | | | | | | -4.78 | | | | | | | | | 5.23E-04 | | | | | | 4.08 | 5.41E-03 | | | |  |
| NM_001020658 | | | | | | | PUM1 | | | | | | | | | -5.57 | | | | | | | | | 4.03E-04 | | | | | | 4.26 | 3.79E-03 | | | |  |
| NM_015317 | | | | | | | PUM2 | | | | | | | | | -4.81 | | | | | | | | | 1.55E-03 | | | | | | 3.89 | 4.49E-03 | | | |  |
| NM_021252 | | | | | | | RAB18 | | | | | | | | | -2.14 | | | | | | | | | 1.27E-03 | | | | | | 2.02 | 6.48E-03 | | | |  |
| NM_020673 | | | | | | | RAB22A | | | | | | | | | -2.19 | | | | | | | | | 9.48E-04 | | | | | | 2.08 | 4.09E-03 | | | |  |
| ENST00000358951 | | | | | | | RAB3GAP2 | | | | | | | | | -2.61 | | | | | | | | | 5.62E-04 | | | | | | 2.09 | 2.81E-03 | | | |  |
| NM_004703 | | | | | | | RABEP1 | | | | | | | | | -3.10 | | | | | | | | | 2.67E-04 | | | | | | 2.32 | 3.36E-03 | | | |  |
| NM_012197 | | | | | | | RABGAP1 | | | | | | | | | -2.86 | | | | | | | | | 1.85E-03 | | | | | | 2.10 | 1.82E-02 | | | |  |
| NM_014504 | RABGEF1 | | | | | | | | | | | -2.00 | | | | | | | | | | | | | | 2.30E-03 | | | | | 2.37 | 9.26E-03 | | | |  |
| NM_020165 | RAD18 | | | | | | | | | | | -2.39 | | | | | | | | | | | | | | 5.57E-04 | | | | | 2.44 | 2.51E-03 | | | |  |
| NM_012415 | RAD54B | | | | | | | | | | | -3.14 | | | | | | | | | | | | | | 3.16E-04 | | | | | 2.52 | 2.88E-03 | | | |  |
| NM_015106 | RAD54L2 | | | | | | | | | | | -3.15 | | | | | | | | | | | | | | 2.45E-03 | | | | | 3.16 | 4.95E-03 | | | |  |
| NM_002880 | RAF1 | | | | | | | | | | | -2.23 | | | | | | | | | | | | | | 1.77E-03 | | | | | 2.16 | 2.88E-03 | | | |  |
| NM_030665 | RAI1 | | | | | | | | | | | -3.73 | | | | | | | | | | | | | | 4.74E-04 | | | | | 3.36 | 4.62E-03 | | | |  |
| NM_005402 | RALA | | | | | | | | | | | -2.81 | | | | | | | | | | | | | | 5.08E-04 | | | | | 2.50 | 3.14E-03 | | | |  |
| NM_014990 | RALGAPA1 | | | | | | | | | | | -3.13 | | | | | | | | | | | | | | 5.05E-03 | | | | | 2.06 | ns. | | | |  |
| NM_020336 | RALGAPB | | | | | | | | | | | -2.53 | | | | | | | | | | | | | | 1.11E-03 | | | | | 2.19 | 3.43E-03 | | | |  |
| NM_005493 | RANBP9 | | | | | | | | | | | -2.13 | | | | | | | | | | | | | | 1.77E-03 | | | | | 2.04 | 3.45E-03 | | | |  |
| NM_014247 | RAPGEF2 | | | | | | | | | | | -10.08 | | | | | | | | | | | | | | 4.32E-04 | | | | | 7.93 | 3.43E-03 | | | |  |
| NM_213589 | RAPH1 | | | | | | | | | | | -2.93 | | | | | | | | | | | | | | 2.09E-03 | | | | | 2.93 | 4.34E-03 | | | |  |
| NM_170692 | RASAL2 | | | | | | | | | | | -10.71 | | | | | | | | | | | | | | 2.05E-04 | | | | | 8.50 | 1.51E-03 | | | |  |
| NM_182663 | RASSF5 | | | | | | | | | | | -2.47 | | | | | | | | | | | | | | 2.52E-03 | | | | | 2.32 | 9.94E-03 | | | |  |
| NM_014892 | RBM16 | | | | | | | | | | | -2.74 | | | | | | | | | | | | | | 2.57E-03 | | | | | 2.86 | 5.70E-03 | | | |  |
| NM_021239 | RBM25 | | | | | | | | | | | -2.19 | | | | | | | | | | | | | | 7.23E-03 | | | | | 2.16 | 6.47E-03 | | | |  |
| NM_022118 | RBM26 | | | | | | | | | | | -2.14 | | | | | | | | | | | | | | 1.40E-03 | | | | | 2.02 | 5.57E-03 | | | |  |
| NM_016836 | RBMS1 (J) | | | | | | | | | | | -3.16 | | | | | | | | | | | | | | 2.87E-03 | | | | | 2.70 | 5.59E-03 | | | |  |
| NM_005349 | RBPJ | | | | | | | | | | | -2.84 | | | | | | | | | | | | | | 4.69E-04 | | | | | 2.56 | 3.11E-03 | | | |  |
| NG_013298 | RBPJP7 | | | | | | | | | | | -2.15 | | | | | | | | | | | | | | 4.01E-03 | | | | | 2.04 | 1.17E-02 | | | |  |
| NM_172071 | RC3H1 | | | | | | | | | | | -2.29 | | | | | | | | | | | | | | 8.89E-04 | | | | | 2.06 | 5.01E-03 | | | |  |
| NM_005772 | RCL1 | | | | | | | | | | | -2.50 | | | | | | | | | | | | | | 8.56E-04 | | | | | 2.53 | 3.26E-03 | | | |  |
| D31888 | RCOR1 | | | | | | | | | | | -4.01 | | | | | | | | | | | | | | 3.61E-04 | | | | | 3.60 | 2.94E-03 | | | |  |
| NM_016316 | REV1 | | | | | | | | | | | -3.54 | | | | | | | | | | | | | | 4.09E-04 | | | | | 2.88 | 3.45E-03 | | | |  |
| NM_002912 | REV3L | | | | | | | | | | | -3.77 | | | | | | | | | | | | | | 1.67E-03 | | | | | 3.01 | 1.82E-02 | | | |  |
| NM_022841 | RFX7 | | | | | | | | | | | -2.78 | | | | | | | | | | | | | | 1.24E-03 | | | | | 2.39 | 3.35E-03 | | | |  |
| NM_033103 | RHPN2 | | | | | | | | | | | -2.65 | | | | | | | | | | | | | | 1.85E-03 | | | | | 2.63 | 1.62E-02 | | | |  |
| NM_152756 | RICTOR | | | | | | | | | | | -4.98 | | | | | | | | | | | | | | 7.58E-04 | | | | | 3.99 | 5.12E-03 | | | |  |
| NM_014715 | RICS | | | | | | | | | | | -2.59 | | | | | | | | | | | | | | 5.26E-03 | | | | | 2.23 | 1.11E-02 | | | |  |
| NM_018151 | RIF1 | | | | | | | | | | | -2.26 | | | | | | | | | | | | | | 1.55E-03 | | | | | 2.09 | 5.40E-03 | | | |  |
| NM_017610 | RNF111 | | | | | | | | | | | -2.94 | | | | | | | | | | | | | | 4.32E-04 | | | | | 2.73 | 2.80E-03 | | | |  |
| NM_018320 | RNF121 | | | | | | | | | | | -2.14 | | | | | | | | | | | | | | 3.42E-03 | | | | | 2.15 | 8.35E-03 | | | |  |
| NM_182757 | | | | | | | RNF144B | | | | | | | | | | | | -3.28 | | | | | | 2.25E-03 | | | | | | 2.61 | 1.25E-02 | | | |  |
| NM_001098638 | | | | | | | RNF169 | | | | | | | | | | | | -3.40 | | | | | | 1.13E-03 | | | | | | 3.06 | 4.25E-03 | | | |  |
| NM_001077239 | | | | | | | RNF214 | | | | | | | | | | | | -2.65 | | | | | | 3.61E-04 | | | | | | 2.64 | 2.41E-03 | | | |  |
| NM_207111 | | | | | | | RNF216 | | | | | | | | | | | | -3.44 | | | | | | 3.68E-04 | | | | | | 3.01 | 2.41E-03 | | | |  |
| NM_152553 | | | | | | | RNF217 | | | | | | | | | | | | -3.45 | | | | | | 3.88E-03 | | | | | | 3.69 | 1.52E-02 | | | |  |
| NM_007219 | | | | | | | RNF24 | | | | | | | | | | | | -2.19 | | | | | | 1.05E-03 | | | | | | 2.35 | 4.48E-03 | | | |  |
| NM_017763 | | | | | | | RNF43 | | | | | | | | | | | | -2.56 | | | | | | 2.00E-02 | | | | | | 2.39 | ns. | | | |  |
| NM_003800 | | | | | | | RNGTT | | | | | | | | | | | | -7.79 | | | | | | 4.74E-04 | | | | | | 5.05 | 5.94E-03 | | | |  |
| NM_005012 | | | | | | | ROR1 | | | | | | | | | | | | -5.21 | | | | | | 8.42E-05 | | | | | | 2.26 | 3.79E-03 | | | |  |
| NM_015209 | | | | | | | RP1-21O18.1 | | | | | | | | | | | | -5.64 | | | | | | 5.80E-04 | | | | | | 3.49 | 1.73E-02 | | | |  |
| NM_015203 | | | | | | | RPRD2 | | | | | | | | | | | | -3.03 | | | | | | 5.03E-03 | | | | | | 2.65 | 5.99E-03 | | | |  |
| NM_004755 | | | | | | | RPS6KA5 | | | | | | | | | | | | -3.05 | | | | | | 1.17E-02 | | | | | | 2.15 | 3.67E-02 | | | |  |
| NM_012424 | | | | | | | RPS6KC1 | | | | | | | | | | | | -3.59 | | | | | | 2.58E-03 | | | | | | 2.13 | 2.07E-03 | | | |  |
| ENST00000379938 | | | | | | | RREB1 | | | | | | | | | | | | -4.28 | | | | | | 4.16E-03 | | | | | | 4.47 | 3.54E-03 | | | |  |
| NM_198467 | | | | | | | RSBN1L | | | | | | | | | | | | -8.34 | | | | | | 2.53E-03 | | | | | | 9.94 | 3.84E-03 | | | |  |
| NM_016578 | | | | | | | RSF1 | | | | | | | | | | | | -4.18 | | | | | | 2.65E-04 | | | | | | 3.43 | 2.60E-03 | | | |  |
| NM_145307 | | | | | | | RTKN2 | | | | | | | | | | | | -4.23 | | | | | | 1.08E-03 | | | | | | 2.89 | 6.53E-03 | | | |  |
| NM_178568 | | | | | | | RTN4RL1 | | | | | | | | | | | | -4.57 | | | | | | 2.48E-03 | | | | | | 4.74 | 7.99E-03 | | | |  |
| NM_001001890 | | | | | | | RUNX1 | | | | | | | | | | | | -4.45 | | | | | | 1.58E-03 | | | | | | 4.36 | 3.77E-03 | | | |  |
| NM_015589 | | | | | | | SAMD4A | | | | | | | | | | | | -2.52 | | | | | | 1.60E-03 | | | | | | 2.41 | 2.92E-02 | | | |  |
| NM_024545 | | | | | | | SAP130 | | | | | | | | | | | | -2.16 | | | | | | 1.10E-03 | | | | | | 2.01 | 3.43E-03 | | | |  |
| NM_013260 | | | | | | | SAP30BP | | | | | | | | | | | | -2.07 | | | | | | 2.91E-03 | | | | | | 2.30 | 6.22E-03 | | | |  |
| NM_015278 | | | | | | | SASH1 | | | | | | | | | | | | -3.70 | | | | | | 3.25E-04 | | | | | | 2.72 | 3.14E-03 | | | |  |
| NM_194292 | | | | | | | SASS6 | | | | | | | | | | | | -3.26 | | | | | | 1.22E-03 | | | | | | 2.64 | 7.07E-03 | | | |  |
| NM_173690 | | | | | | | SCAI | | | | | | | | | | | | -6.76 | | | | | | 4.32E-04 | | | | | | 4.58 | 3.17E-03 | | | |  |
| NM_014575 | | | | | | | SCHIP1 | | | | | | | | | | | | -4.81 | | | | | | 3.61E-04 | | | | | | 4.18 | 2.85E-03 | | | |  |
| NM_012430 | | | | | | | SEC22A | | | | | | | | | | | | -2.58 | | | | | | 3.25E-04 | | | | | | 2.50 | 2.53E-03 | | | |  |
| NM_032970 | | | | | | | SEC22C | | | | | | | | | | | | -2.22 | | | | | | 1.42E-03 | | | | | | 2.49 | 3.36E-03 | | | |  |
| NM_007190 | | | | | | | SEC23IP | | | | | | | | | | | | -2.33 | | | | | | 6.30E-04 | | | | | | 2.04 | 6.17E-03 | | | |  |
| NM_021982 | | | | | | | SEC24A | | | | | | | | | | | | -2.63 | | | | | | 1.13E-03 | | | | | | 2.26 | 4.95E-03 | | | |  |
| NM_006323 | | | | | | | SEC24B | | | | | | | | | | | | -4.35 | | | | | | 6.44E-04 | | | | | | 3.90 | 4.95E-03 | | | |  |
| NM_033505 | | | | | | | SELI | | | | | | | | | | | | -2.20 | | | | | | 6.44E-04 | | | | | | 2.11 | 5.63E-03 | | | |  |
| NM_006080 | | | | | SEMA3A | | | | | | | | | | | | | | -3.39 | | | | | | 3.16E-03 | | | | | | 2.17 | 4.11E-02 | | | |  |
| NM_014554 | | | | | SENP1 | | | | | | | | | | | | | | -2.40 | | | | | | 1.14E-03 | | | | | | 2.10 | 7.16E-03 | | | |  |
| NM_014159 | | | | | SETD2 | | | | | | | | | | | | | | -2.15 | | | | | | 1.47E-03 | | | | | | 2.07 | 6.66E-03 | | | |  |
| NM_015046 | | | | | SETX | | | | | | | | | | | | | | -3.19 | | | | | | 9.81E-03 | | | | | | 2.79 | 4.11E-02 | | | |  |
| NM_001005159 | | | | | SFMBT1 | | | | | | | | | | | | | | -2.46 | | | | | | 6.85E-04 | | | | | | 2.43 | 3.11E-03 | | | |  |
| NM_004592 | | | | | SFRS8 | | | | | | | | | | | | | | -2.68 | | | | | | 6.85E-04 | | | | | | 2.73 | 3.79E-03 | | | |  |
| AB073386 | | | | | SGEF | | | | | | | | | | | | | | -4.63 | | | | | | 1.13E-03 | | | | | | 4.54 | ns. | | | |  |
| NM_147156 | | | | | SGMS1 | | | | | | | | | | | | | | -3.85 | | | | | | 3.50E-04 | | | | | | 2.85 | 3.86E-03 | | | |  |
| NM_014631 | | | | | SH3PXD2A | | | | | | | | | | | | | | -6.94 | | | | | | 8.35E-04 | | | | | | 4.99 | 4.07E-03 | | | |  |
| NM_020870 | | | | | SH3RF1 | | | | | | | | | | | | | | -4.52 | | | | | | 1.91E-04 | | | | | | 4.12 | 2.07E-03 | | | |  |
| NM_024577 | | | | | SH3TC2 | | | | | | | | | | | | | | -3.60 | | | | | | 3.34E-03 | | | | | | 3.44 | 1.79E-02 | | | |  |
| NM_003028 | | | | | SHB | | | | | | | | | | | | | | -3.88 | | | | | | 8.19E-04 | | | | | | 3.08 | 3.57E-03 | | | |  |
| NM_007373 | | | | | SHOC2 | | | | | | | | | | | | | | -3.34 | | | | | | 1.27E-03 | | | | | | 2.52 | 4.54E-03 | | | |  |
| NM_018130 | | | | | SHQ1 | | | | | | | | | | | | | | -2.74 | | | | | | 4.61E-03 | | | | | | 2.48 | 8.49E-03 | | | |  |
| NM_020859 | | | | | SHROOM3 | | | | | | | | | | | | | | -3.88 | | | | | | 1.57E-03 | | | | | | 3.57 | 9.94E-03 | | | |  |
| NM_025164 | | | | | SIK3 | | | | | | | | | | | | | | -2.80 | | | | | | 4.06E-03 | | | | | | 2.25 | 3.47E-02 | | | |  |
| NM_015477 | | | | | SIN3A | | | | | | | | | | | | | | -3.27 | | | | | | 9.93E-04 | | | | | | 3.02 | 4.35E-03 | | | |  |
| BC031691 | | | | | SLAIN2 | | | | | | | | | | | | | | -2.21 | | | | | | 1.04E-03 | | | | | | 2.07 | 1.34E-02 | | | |  |
| NM_006749 | | | | | SLC20A2 | | | | | | | | | | | | | | -5.08 | | | | | | 8.50E-04 | | | | | | 3.75 | 9.52E-03 | | | |  |
| NM_003705 | | | | | SLC25A12 | | | | | | | | | | | | | | -5.51 | | | | | | 4.55E-04 | | | | | | 3.67 | 3.48E-03 | | | |  |
| NM_080669 | | | | | SLC46A1 | | | | | | | | | | | | | | -2.06 | | | | | | 1.60E-02 | | | | | | 2.77 | 9.91E-03 | | | |  |
| NM_013272 | | | | | SLCO3A1 (J) | | | | | | | | | | | | | | -4.93 | | | | | | 1.17E-05 | | | | | | 2.39 | 2.60E-03 | | | |  |
| NM_005900 | | | | | SMAD1 | | | | | | | | | | | | | | -2.21 | | | | | | 4.07E-04 | | | | | | 2.00 | 2.41E-03 | | | |  |
| NM_005902 | | | | | SMAD3 | | | | | | | | | | | | | | -3.92 | | | | | | 1.70E-03 | | | | | | 3.15 | 4.78E-03 | | | |  |
| NM_020159 | | | | | SMARCAD1 | | | | | | | | | | | | | | -3.36 | | | | | | 2.67E-04 | | | | | | 2.87 | 3.14E-03 | | | |  |
| AK002200 | | | | | SMC4 | | | | | | | | | | | | | | -3.00 | | | | | | 5.57E-04 | | | | | | 2.73 | 4.01E-03 | | | |  |
| NM_032560 | | | | | SMEK1 | | | | | | | | | | | | | | -2.52 | | | | | | 7.13E-03 | | | | | | 2.45 | 2.07E-02 | | | |  |
| NM_017575 | | | | | SMG6 | | | | | | | | | | | | | | -2.55 | | | | | | 4.98E-04 | | | | | | 2.00 | 2.51E-03 | | | |  |
| NM_020429 | | | | | SMURF1 | | | | | | | | | | | | | | -2.86 | | | | | | 1.43E-03 | | | | | | 2.40 | 9.86E-03 | | | |  |
| NM_022739 | | | | | SMURF2 (J) | | | | | | | | | | | | | | -3.95 | | | | | | 1.44E-03 | | | | | | 3.18 | 8.73E-03 | | | |  |
| NM_015132 | | | | | SNX13 | | | | | | | | | | | | | | -3.18 | | | | | | 3.16E-03 | | | | | | 2.50 | 2.85E-02 | | | |  |
| NM_016224 | | | | | SNX9 | | | | | | | | | | | | | | -3.04 | | | | | | 2.26E-03 | | | | | | 2.50 | 2.26E-02 | | | |  |
| NM_005686 | | | | | SOX13 | | | | | | | | | | | | | | | | | | -2.49 | | 2.12E-03 | | | | | | 2.38 | 4.01E-03 | | | |  |
| BX648857 | | | | | SP3 | | | | | | | | | | | | | | | | | | -2.54 | | 1.27E-03 | | | | | | 2.06 | 9.36E-03 | | | |  |
| NM_153023 | | | | | SPATA13 | | | | | | | | | | | | | | | | | | -3.47 | | 1.74E-03 | | | | | | 3.39 | 3.79E-03 | | | |  |
| NM_023071 | | | | | SPATS2 | | | | | | | | | | | | | | | | | | -2.65 | | 1.58E-03 | | | | | | 2.09 | 1.15E-02 | | | |  |
| NM_015001 | | | | | SPEN | | | | | | | | | | | | | | | | | | -2.65 | | 2.24E-02 | | | | | | 3.38 | 4.43E-03 | | | |  |
| NM_015087 | | | | | SPG20 | | | | | | | | | | | | | | | | | | -2.54 | | 3.16E-04 | | | | | | 2.02 | 2.88E-03 | | | |  |
| NM_020148 | | | | | SPIRE1 | | | | | | | | | | | | | | | | | | -3.59 | | 1.91E-04 | | | | | | 2.50 | 3.45E-03 | | | |  |
| NM_001001664 | | | | | SPOPL | | | | | | | | | | | | | | | | | | -2.55 | | 1.20E-03 | | | | | | 2.31 | 7.18E-03 | | | |  |
| NM_152594 | | | | | SPRED1 | | | | | | | | | | | | | | | | | | -4.02 | | 6.34E-04 | | | | | | 3.33 | 2.90E-03 | | | |  |
| NM_025106 | | | | | SPSB1 | | | | | | | | | | | | | | | | | | -2.39 | | 1.99E-03 | | | | | | 2.38 | 6.20E-03 | | | |  |
| NM_005417 | | | | | SRC | | | | | | | | | | | | | | | | | | -2.27 | | 3.01E-02 | | | | | | 2.15 | 1.65E-02 | | | |  |
| NM_152546 | | | | | SRFBP1 | | | | | | | | | | | | | | | | | | -2.12 | | 6.44E-04 | | | | | | 2.12 | 3.59E-03 | | | |  |
| NM_020762 | | | | | SRGAP1 | | | | | | | | | | | | | | | | | | -6.43 | | 5.10E-04 | | | | | | 5.55 | 2.51E-03 | | | |  |
| NM_182691 | | | | | SRPK2 | | | | | | | | | | | | | | | | | | -3.38 | | 8.21E-04 | | | | | | 2.81 | 2.85E-03 | | | |  |
| NM_018984 | | | | | SSH1 | | | | | | | | | | | | | | | | | | -2.15 | | 1.16E-02 | | | | | | 2.03 | 3.50E-02 | | | |  |
| NM_003033 | | | | | ST3GAL1 | | | | | | | | | | | | | | | | | | -3.80 | | 7.27E-04 | | | | | | 4.57 | 3.26E-03 | | | |  |
| NM_030965 | | | | | ST6GALNAC5 | | | | | | | | | | | | | | | | | | -3.33 | | 7.16E-03 | | | | | | 3.97 | 1.40E-02 | | | |  |
| NM_003473 | | | | | STAM | | | | | | | | | | | | | | | | | | -2.57 | | 1.73E-03 | | | | | | 2.64 | 8.99E-03 | | | |  |
| NM_020799 | | | | | STAMBPL1 | | | | | | | | | | | | | | | | | | -3.34 | | 4.98E-04 | | | | | | 3.06 | 3.79E-03 | | | |  |
| NM_005990 | | | | | STK10 | | | | | | | | | | | | | | | | | | -2.78 | | 1.72E-03 | | | | | | 2.33 | 1.24E-02 | | | |  |
| NM_004760 | | | | | STK17A | | | | | | | | | | | | | | | | | | -2.85 | | 8.81E-04 | | | | | | 2.30 | 5.64E-03 | | | |  |
| NM_001032296 | | | | | STK24 | | | | | | | | | | | | | | | | | | -2.97 | | 4.03E-04 | | | | | | 2.11 | 4.43E-03 | | | |  |
| AK094799 | | | | | STON2 | | | | | | | | | | | | | | | | | | -2.26 | | 1.11E-02 | | | | | | 2.04 | 4.08E-02 | | | |  |
| NM_014574 | | | | | STRN3 | | | | | | | | | | | | | | | | | | -6.28 | | 9.54E-04 | | | | | | 4.16 | 9.22E-03 | | | |  |
| NM_017919 | | | | | STX17 | | | | | | | | | | | | | | | | | | -3.04 | | 6.46E-04 | | | | | | 2.36 | 3.11E-03 | | | |  |
| NM_016930 | | | | | STX18 | | | | | | | | | | | | | | | | | | -2.56 | | 4.51E-04 | | | | | | 2.11 | 2.88E-03 | | | |  |
| NM_005819 | | | | | STX6 | | | | | | | | | | | | | | | | | | -2.31 | | 3.68E-04 | | | | | | 2.06 | 3.45E-03 | | | |  |
| NM_018423 | | | | | STYK1 | | | | | | | | | | | | | | | | | | -2.30 | | 5.44E-03 | | | | | | 2.01 | ns. | | | |  |
| NM_003898 | | | | | SYNJ2 | | | | | | | | | | | | | | | | | | -2.58 | | 1.78E-03 | | | | | | 2.67 | 5.55E-03 | | | |  |
| NM_007247 | | | | | SYNRG | | | | | | | | | | | | | | | | | | -2.75 | | 6.21E-04 | | | | | | 2.67 | 3.55E-03 | | | |  |
| NM_206862 | | | | | TACC2 | | | | | | | | | | | | | | | | | | -3.69 | | 1.35E-03 | | | | | | 2.91 | 1.12E-02 | | | |  |
| NM_001488 | | | | | TADA2A | | | | | | | | | | | | | | | | | | -2.26 | | 6.62E-04 | | | | | | 2.56 | 1.58E-03 | | | |  |
| NM_031923 | | | | | TAF3 | | | | | | | | | | | | -4.04 | | | | | | | | 6.75E-04 | | | | | | 3.34 | 9.18E-03 | | | |  |
| NM_003185 | | | | | TAF4 | | | | | | | | | | | | -2.96 | | | | | | | | 8.19E-04 | | | | | | 2.67 | 3.45E-03 | | | |  |
| NM_033394 | | | | | TANC1 | | | | | | | | | | | | -9.85 | | | | | | | | 5.99E-04 | | | | | | 6.80 | 3.81E-03 | | | |  |
| NM_020791 | | | | | TAOK1 | | | | | | | | | | | | -4.12 | | | | | | | | 3.25E-04 | | | | | | 3.00 | 2.88E-03 | | | |  |
| NM_017714 | | | | | TASP1 | | | | | | | | | | | | -4.72 | | | | | | | | 1.16E-04 | | | | | | 3.00 | 2.07E-03 | | | |  |
| AJ404330 | | | | | TBC1D12 | | | | | | | | | | | | -7.09 | | | | | | | | 3.67E-04 | | | | | | 5.92 | 2.90E-03 | | | |  |
| NM_018421 | | | | | TBC1D2 | | | | | | | | | | | | -2.51 | | | | | | | | 9.30E-03 | | | | | | 2.49 | 3.20E-02 | | | |  |
| NM_017772 | | | | | TBC1D22B | | | | | | | | | | | | -2.43 | | | | | | | | 1.66E-03 | | | | | | 2.23 | 1.13E-02 | | | |  |
| NM_005650 | | | | | TCF20 | | | | | | | | | | | | -2.02 | | | | | | | | 5.26E-03 | | | | | | 2.10 | 3.54E-03 | | | |  |
| NM_001146274 | | | | | TCF7L2 | | | | | | | | | | | | -8.77 | | | | | | | | 1.70E-03 | | | | | | 6.73 | 8.56E-03 | | | |  |
| NM_014844 | | | | | TECPR2 | | | | | | | | | | | | -4.77 | | | | | | | | 2.26E-03 | | | | | | 4.12 | 7.05E-03 | | | |  |
| NM_001127208 | | | | | TET2 | | | | | | | | | | | | -9.83 | | | | | | | | 7.43E-04 | | | | | | 7.13 | 5.26E-03 | | | |  |
| NM_017746 | | | | | TEX10 | | | | | | | | | | | | -2.13 | | | | | | | | 3.26E-03 | | | | | | 2.17 | 1.72E-02 | | | |  |
| NM_018469 | | | | | TEX2 | | | | | | | | | | | | -2.42 | | | | | | | | 1.96E-03 | | | | | | 2.00 | 5.55E-03 | | | |  |
| NM_005653 | | | | | TFCP2 | | | | | | | | | | | | -2.02 | | | | | | | | 1.97E-03 | | | | | | 2.07 | 1.18E-02 | | | |  |
| NM_001024847 | | | | | TGFBR2 | | | | | | | | | | | | -4.15 | | | | | | | | 4.42E-04 | | | | | | 3.13 | 4.42E-03 | | | |  |
| NM_005077 | | | | | TLE1 | | | | | | | | | | | | -2.58 | | | | | | | | 4.35E-03 | | | | | | 2.81 | 9.06E-03 | | | |  |
| NM_007005 | | | | | TLE4 | | | | | | | | | | | | -3.95 | | | | | | | | 1.30E-03 | | | | | | 3.37 | 6.43E-03 | | | |  |
| NM_006852 | | | | | TLK2 | | | | | | | | | | | | -4.60 | | | | | | | | 4.27E-04 | | | | | | 3.89 | 3.11E-03 | | | |  |
| NM_001017395 | | | | | TMCC1 | | | | | | | | | | | | -6.87 | | | | | | | | 3.50E-04 | | | | | | 6.36 | 2.88E-03 | | | |  |
| NM_024562 | | | | | TMCO7 | | | | | | | | | | | | -3.35 | | | | | | | | 3.90E-05 | | | | | | 2.08 | 3.85E-03 | | | |  |
| NM_032021 | | | | | TMEM133 | | | | | | | | | | | | -29.66 | | | | | | | | 9.37E-04 | | | | | | 20.39 | ns. | | | |  |
| NM_153354 | | | | | TMEM161B | | | | | | | | | | | | -3.67 | | | | | | | | 7.10E-04 | | | | | | 2.89 | 3.81E-03 | | | |  |
| NM_018022 | | | | | TMEM51 | | | | | | | | | | | | -2.04 | | | | | | | | 4.65E-04 | | | | | | 2.13 | 3.14E-03 | | | |  |
| NM_014452 | | | | | TNFRSF21 | | | | | | | | | | | | -2.74 | | | | | | | | 1.26E-02 | | | | | | 2.29 | 1.60E-02 | | | |  |
| NM_003747 | | | | | TNKS | | | | | | | | | | | | -2.21 | | | | | | | | 1.12E-03 | | | | | | 2.16 | 6.67E-03 | | | |  |
| NM_025235 | | | | | TNKS2 | | | | | | | | | | | | -2.48 | | | | | | | | 6.45E-03 | | | | | | 2.03 | 1.59E-02 | | | |  |
| NM_014494 | | | | | TNRC6A | | | | | | | | | | | | -2.40 | | | | | | | | 1.43E-03 | | | | | | 2.24 | 5.21E-03 | | | |  |
| NM_015088 | | | | | TNRC6B | | | | | | | | | | | | -2.67 | | | | | | | | 1.09E-03 | | | | | | 2.08 | 3.54E-03 | | | |  |
| NM_022748 | | | | | TNS3 | | | | | | | | | | | | -5.03 | | | | | | | | 6.62E-04 | | | | | | 3.02 | 3.14E-03 | | | |  |
| NM_032865 | | | | | TNS4 | | | | | | | | | | | | -2.43 | | | | | | | | 4.11E-02 | | | | | | 2.97 | ns. | | | |  |
| AK055959 | | | | | TOM1L2 | | | | | | | | | | | | -4.05 | | | | | | | | 7.84E-03 | | | | | | 3.05 | 1.70E-02 | | | |  |
| NM_005426 | | | | | TP53BP2 | | | | | | | | | | | | -2.43 | | | | | | | | 1.05E-03 | | | | | | 2.53 | 3.92E-03 | | | |  |
| NM_003722 | | | | | | | TP63 | | | | | | | | -4.10 | | | | | | | | | | 3.40E-04 | | | | | | 3.16 | 2.60E-03 | | | |  |
| NM_003596 | | | | | | | TPST1 | | | | | | | | -5.18 | | | | | | | | | | 9.67E-04 | | | | | | 4.09 | 1.34E-02 | | | |  |
| NM_145725 | | | | | | | TRAF3 | | | | | | | | -3.37 | | | | | | | | | | 6.42E-04 | | | | | | 2.98 | 3.43E-03 | | | |  |
| NM_012288 | | | | | | | TRAM2 (J) | | | | | | | | -2.54 | | | | | | | | | | 8.22E-03 | | | | | | 2.23 | 2.83E-02 | | | |  |
| NM_003274 | | | | | | | TRAPPC10 | | | | | | | | -2.40 | | | | | | | | | | 7.43E-04 | | | | | | 2.12 | 3.20E-03 | | | |  |
| NM_033502 | | | | | | | TRERF1 | | | | | | | | -2.73 | | | | | | | | | | 5.26E-03 | | | | | | 2.62 | 1.95E-02 | | | |  |
| NM_015905 | | | | | | | TRIM24 | | | | | | | | -3.77 | | | | | | | | | | 4.88E-04 | | | | | | 3.08 | 3.76E-03 | | | |  |
| NM_033020 | | | | | | | TRIM33 | | | | | | | | -2.56 | | | | | | | | | | 2.26E-03 | | | | | | 2.23 | 5.51E-03 | | | |  |
| NM_015294 | | | | | | | TRIM37 | | | | | | | | -2.55 | | | | | | | | | | 7.20E-04 | | | | | | 2.34 | 3.45E-03 | | | |  |
| NM_007118 | | | | | | | TRIO | | | | | | | | -2.84 | | | | | | | | | | 1.15E-02 | | | | | | 2.34 | 1.21E-02 | | | |  |
| NM_025265 | | | | | | | TSEN2 | | | | | | | | -2.16 | | | | | | | | | | 5.66E-04 | | | | | | 2.08 | 2.57E-03 | | | |  |
| NM_030927 | | | | | | | TSPAN14 | | | | | | | | -3.00 | | | | | | | | | | 7.85E-04 | | | | | | 2.54 | 5.64E-03 | | | |  |
| NM_005723 | | | | | | | TSPAN5 | | | | | | | | -3.82 | | | | | | | | | | 1.34E-03 | | | | | | 2.68 | 2.63E-02 | | | |  |
| NM_001040655 | | | | | | | TTC23 | | | | | | | | -2.95 | | | | | | | | | | 4.45E-04 | | | | | | 2.37 | 2.57E-03 | | | |  |
| NM_020245 | | | | | | | TULP4 | | | | | | | | -3.74 | | | | | | | | | | 4.60E-03 | | | | | | 2.30 | 1.48E-02 | | | |  |
| NM_015914 | | | | | | | TXNDC11 | | | | | | | | -2.40 | | | | | | | | | | 1.61E-03 | | | | | | 2.33 | 3.54E-03 | | | |  |
| NM_003330 | | | | | | | TXNRD1 | | | | | | | | -2.55 | | | | | | | | | | 2.65E-03 | | | | | | 2.39 | 2.47E-02 | | | |  |
| NM_018264 | | | | | | | TYW1 | | | | | | | | -4.11 | | | | | | | | | | 3.51E-04 | | | | | | 2.86 | 2.88E-03 | | | |  |
| NM_018449 | | | | | | | UBAP2 | | | | | | | | -2.18 | | | | | | | | | | 3.02E-03 | | | | | | 2.23 | 4.47E-03 | | | |  |
| NM_003342 | | | | | | | UBE2G1 | | | | | | | | -3.66 | | | | | | | | | | 1.49E-02 | | | | | | 2.46 | 4.28E-03 | | | |  |
| NM_003344 | | | | | | | UBE2H | | | | | | | | -2.67 | | | | | | | | | | 4.09E-04 | | | | | | 2.33 | 5.39E-03 | | | |  |
| NM_005339 | | | | | | | UBE2K | | | | | | | | -2.93 | | | | | | | | | | 3.61E-04 | | | | | | 2.40 | 2.60E-03 | | | |  |
| NM_001001481 | | | | | | | UBE2W | | | | | | | | -2.45 | | | | | | | | | | 2.10E-03 | | | | | | 2.18 | 4.29E-03 | | | |  |
| NM_130839 | | | | | | | UBE3A | | | | | | | | -2.49 | | | | | | | | | | 9.60E-04 | | | | | | 2.26 | 4.70E-03 | | | |  |
| NM_174916 | | | | | | | UBR1 | | | | | | | | -3.35 | | | | | | | | | | 3.25E-04 | | | | | | 2.71 | 2.57E-03 | | | |  |
| ENST00000372899 | | | | | | | UBR2 | | | | | | | | -3.17 | | | | | | | | | | 3.51E-04 | | | | | | 2.52 | 3.45E-03 | | | |  |
| NM_012474 | | | | | | | UCK2 | | | | | | | | -2.48 | | | | | | | | | | 2.52E-03 | | | | | | 2.14 | 1.61E-02 | | | |  |
| NM_016290 | | | | | | | UIMC1 | | | | | | | | -2.72 | | | | | | | | | | 2.57E-04 | | | | | | 2.47 | 2.41E-03 | | | |  |
| NM_139015 | | | | | | | UNQ1887 | | | | | | | | -3.42 | | | | | | | | | | 9.78E-04 | | | | | | 2.59 | 2.88E-03 | | | |  |
| BC010099 | | | | | | | USB1 | | | | | | | | -2.87 | | | | | | | | | | 6.70E-04 | | | | | | 3.69 | 3.20E-03 | | | |  |
| NM_182488 | | | | | | | USP12 | | | | | | | | -4.08 | | | | | | | | | | 7.13E-04 | | | | | | 2.47 | 6.58E-03 | | | |  |
| NM_003940 | | | | | | | USP13 | | | | | | | | -2.45 | | | | | | | | | | 9.24E-03 | | | | | | 2.06 | ns. | | | |  |
| ENST00000294383 | | | | | | | USP24 | | | | | | | | | | | | | -3.04 | | | | | 1.21E-03 | | | | | | 2.44 | 5.12E-03 | | | |  |
| NM_006537 | | | | | | | USP3 | | | | | | | | | | | | | -2.32 | | | | | 1.71E-03 | | | | | | 2.36 | 4.84E-03 | | | |  |
| NM_020718 | | | | | | | USP31 | | | | | | | | | | | | | -2.10 | | | | | 2.68E-03 | | | | | | 2.01 | 2.58E-03 | | | |  |
| NM_014709 | | | | | | | USP34 | | | | | | | | | | | | | -2.94 | | | | | 1.62E-03 | | | | | | 2.10 | 3.14E-03 | | | |  |
| NM_020935 | | | | | | | USP37 | | | | | | | | | | | | | -5.05 | | | | | 7.71E-04 | | | | | | 4.66 | 4.56E-03 | | | |  |
| AK090821 | | | | | | | USP43 | | | | | | | | | | | | | -2.22 | | | | | 6.56E-03 | | | | | | 2.51 | 2.54E-02 | | | |  |
| NM_017944 | | | | | | | USP47 | | | | | | | | | | | | | -2.92 | | | | | 1.04E-03 | | | | | | 2.07 | 4.83E-03 | | | |  |
| AK023682 | | | | | | | USP49 | | | | | | | | | | | | | -2.68 | | | | | 1.04E-03 | | | | | | 2.37 | 5.83E-03 | | | |  |
| BC017382 | | | | | | | USP53 | | | | | | | | | | | | | -2.42 | | | | | 2.04E-03 | | | | | | 2.19 | 8.56E-03 | | | |  |
| BC110845 | | | | | | | USP54 | | | | | | | | | | | | | -2.67 | | | | | 4.69E-04 | | | | | | 2.70 | 2.51E-03 | | | |  |
| BC042943 | | | | | | | USP6NL | | | | | | | | | | | | | -4.62 | | | | | 3.25E-04 | | | | | | 3.66 | 3.79E-03 | | | |  |
| NM_138959 | | | | | | | VANGL1 | | | | | | | | | | | | | -2.50 | | | | | 1.05E-03 | | | | | | 2.04 | 5.68E-03 | | | |  |
| NM_003371 | | | | | | | VAV2 | | | | | | | | | | | | | -3.94 | | | | | 5.66E-04 | | | | | | 2.78 | 3.45E-03 | | | |  |
| NM_005429 | | | | | | | VEGFC 🟋 (J) | | | | | | | | | | | | | -4.41 | | | | | 7.10E-04 | | | | | | 4.16 | 3.54E-03 | | | |  |
| NM_017599 | | | | | | | VEZT | | | | | | | | | | | | | -2.61 | | | | | 7.69E-04 | | | | | | 2.09 | 4.01E-03 | | | |  |
| NM_016516 | | | | | | | VPS54 | | | | | | | | | | | | | -3.35 | | | | | 3.25E-04 | | | | | | 2.53 | 2.80E-03 | | | |  |
| NM_100264 | | | | | | | WAC | | | | | | | | | | | | | -3.36 | | | | | 5.12E-04 | | | | | | 2.76 | 4.20E-03 | | | |  |
| NM_015045 | | | | | | | WAPAL | | | | | | | | | | | | | -2.80 | | | | | 7.35E-04 | | | | | | 2.94 | 4.80E-03 | | | |  |
| NM_052950 | | | | | | | WDFY2 | | | | | | | | | | | | | -2.81 | | | | | 4.77E-04 | | | | | | 2.25 | 1.20E-02 | | | |  |
| NM_025160 | | | | | | | WDR26 | | | | | | | | | | | | | -2.22 | | | | | 8.35E-04 | | | | | | 2.01 | 9.94E-03 | | | |  |
| NM_018383 | | | | | | | WDR33 | | | | | | | | | | | | | -3.52 | | | | | 9.43E-04 | | | | | | 2.91 | 5.16E-03 | | | |  |
| NM_014023 | | | | | | | WDR37 | | | | | | | | | | | | | -2.16 | | | | | 3.03E-03 | | | | | | 2.37 | 5.15E-03 | | | |  |
| D87716 | | | | | | | WDR43 | | | | | | | | | | | | | -2.98 | | | | | 4.55E-04 | | | | | | 2.64 | 2.57E-03 | | | |  |
| NM_019045 | | | | | | | WDR44 | | | | | | | | | | | | | -3.59 | | | | | 4.55E-04 | | | | | | 2.91 | 4.81E-03 | | | |  |
| NM_014969 | | | | | | | WDR47 | | | | | | | | | | | | | -2.52 | | | | | 5.08E-04 | | | | | | 2.30 | 3.99E-03 | | | |  |
| NM_018051 | | | | | | | WDR60 | | | | | | | | | | | | | -2.80 | | | | | 1.13E-03 | | | | | | 2.15 | ns. | | | |  |
| NM_015285 | | | | | | | WDR7 | | | | | | | | | | | | | -4.81 | | | | | 7.09E-06 | | | | | | 2.18 | 6.57E-03 | | | |  |
| NM_017778 | | | | | | | WHSC1L1 | | | | | | | | | | | | | -2.53 | | | | | 1.54E-03 | | | | | | 2.17 | 5.34E-03 | | | |  |
| NM_133264 | | | | | | | WIPF2 | | | | | | | | | | | | | -2.55 | | | | | 1.78E-03 | | | | | | 2.56 | 4.18E-03 | | | |  |
| BC035146 | | | | | | | WNK1 | | | | | | | | | | | | | -2.66 | | | | | 1.17E-03 | | | | | | 2.17 | 4.78E-03 | | | |  |
| NM_004625 | | | | | | | WNT7A (J) | | | | | | | | | | | | | -2.87 | | | | | 4.96E-03 | | | | | | 3.40 | 8.66E-03 | | | |  |
| NM_058238 | | | | | | | WNT7B | | | | | | | | | | | | | -2.70 | | | | | 5.58E-03 | | | | | | 2.27 | 3.50E-02 | | | |  |
| NM_015238 | | | | | | | WWC1 | | | | | | -4.60 | | | | | | | | | | | | 5.43E-04 | | | | | | 3.80 | 4.62E-03 | | | |  |
| NM_007013 | | | | | | | WWP1 | | | | | | -3.64 | | | | | | | | | | | | 5.57E-04 | | | | | | 2.86 | 4.80E-03 | | | |  |
| NM_015472 | | | | | | | WWTR1 | | | | | | -2.49 | | | | | | | | | | | | 1.34E-03 | | | | | | 2.17 | 1.28E-02 | | | |  |
| NM_001270940 | | | | | | | XPO6 | | | | | | -2.93 | | | | | | | | | | | | 6.86E-04 | | | | | | 2.35 | 5.39E-03 | | | |  |
| NM_018023 | | | | | | | YEATS2 | | | | | | -2.74 | | | | | | | | | | | | 3.29E-04 | | | | | | 2.40 | 3.97E-03 | | | |  |
| NM_016653 | | | | | | | ZAK | | | | | | -3.74 | | | | | | | | | | | | 2.58E-04 | | | | | | 2.42 | 3.82E-03 | | | |  |
| ENST00000278590 | | | | | | | ZC3H12C | | | | | | -2.34 | | | | | | | | | | | | 1.80E-03 | | | | | | 2.16 | 1.83E-02 | | | |  |
| ENST00000253048 | | | | | | | ZC3H4 | | | | | | -2.07 | | | | | | | | | | | | 6.07E-03 | | | | | | 2.30 | 2.57E-03 | | | |  |
| NM_001009881 | | | | | | | ZCCHC11 | | | | | | -3.93 | | | | | | | | | | | | 3.40E-04 | | | | | | 2.89 | 3.00E-03 | | | |  |
| NM_015144 | | | | | | | ZCCHC14 | | | | | | -2.46 | | | | | | | | | | | | 5.27E-03 | | | | | | 2.49 | 1.66E-02 | | | |  |
| NM_017742 | | | | | | | ZCCHC2 | | | | | | -2.51 | | | | | | | | | | | | 1.33E-03 | | | | | | 2.29 | 9.26E-03 | | | |  |
| AY629351 | | | | | | | ZCCHC4 | | | | | | -2.29 | | | | | | | | | | | | 1.11E-03 | | | | | | 2.16 | 3.48E-03 | | | |  |
| NM_024617 | | | | | | | ZCCHC6 | | | | | | -2.36 | | | | | | | | | | | | 3.23E-03 | | | | | | 2.27 | 1.63E-02 | | | |  |
| NM_032226 | | | | | | | ZCCHC7 | | | | | | -4.87 | | | | | | | | | | | | 1.42E-03 | | | | | | 3.24 | 7.63E-03 | | | |  |
| NM_015336 | | | | | | | ZDHHC17 | | | | | | -3.60 | | | | | | | | | | | | 2.84E-04 | | | | | | 2.59 | 3.23E-03 | | | |  |
| NM_020863 | | | | | | | ZFAT | | | | | | -3.19 | | | | | | | | | | | | 4.09E-04 | | | | | | 2.30 | 2.51E-03 | | | |  |
| NM_006885 | | | | | | | ZFHX3 | | | | | | -2.47 | | | | | | | | | | | | 4.87E-02 | | | | | | 2.25 | 4.81E-03 | | | |  |
| NM_053023 | | | | | | | ZFP91 | | | | | | -2.35 | | | | | | | | | | | | 4.11E-03 | | | | | | 2.20 | 4.29E-03 | | | |  |
| NM_003410 | | | | | | | ZFX | | | | | | -2.13 | | | | | | | | | | | | 5.49E-03 | | | | | | 2.18 | 8.41E-03 | | | |  |
| NM_004799 | | | | | | | ZFYVE9 | | | | | | -3.93 | | | | | | | | | | | | 1.47E-03 | | | | | | 2.81 | 2.71E-02 | | | |  |
| NM_015035 | | | | | | | ZHX3 | | | | | | -5.62 | | | | | | | | | | | | 5.90E-04 | | | | | | 3.98 | 3.17E-03 | | | |  |
| NM_020338 | | | | | | | ZMIZ1 | | | | | | -7.67 | | | | | | | | | | | | 1.87E-03 | | | | | | 5.72 | 4.54E-03 | | | |  |
| NM_003453 | | | | | | | ZMYM2 | | | | | | -3.23 | | | | | | | | | | | | 6.89E-04 | | | | | | 2.79 | 5.31E-03 | | | |  |
| NM_012408 | | | | | | | ZMYND8 | | | | | | -4.03 | | | | | | | | | | | | 3.72E-03 | | | | | | 3.17 | 2.55E-02 | | | |  |
| NM_021964 | | | | | | | ZNF148 | | | | | | -3.52 | | | | | | | | | | | | 2.67E-04 | | | | | | 2.79 | 3.43E-03 | | | |  |
| NM_021994 | | | | | | | ZNF277 | | | | | | -4.52 | | | | | | | | | | | | 5.42E-04 | | | | | | 3.57 | 3.81E-03 | | | |  |
| NM_015021 | | | | | | | ZNF292 | | | | | | -2.53 | | | | | | | | | | | | 4.86E-03 | | | | | | 2.14 | 3.27E-02 | | | |  |
| NM_001031623 | | | | | | | ZNF451 | | | | | | -2.44 | | | | | | | | | | | | 8.83E-04 | | | | | | 2.31 | 8.84E-03 | | | |  |
| NM_021224 | | | | | | | ZNF462 | | | | | | -2.62 | | | | | | | | | | | | 1.20E-02 | | | | | | 2.53 | 1.78E-02 | | | |  |
| NM_014643 | | | | | | | ZNF516 | | | | | | -2.79 | | | | | | | | | | | | 5.30E-03 | | | | | | 2.19 | 1.50E-02 | | | |  |
| NM_015042 | | | | | | | ZNF609 | | | | | | -4.94 | | | | | | | | | | | | 3.51E-04 | | | | | | 3.65 | 4.43E-03 | | | |  |
| AJ420454 | | | | | | | ZNF618 | | | -3.81 | | | | | | | | | | | | | | | 1.15E-03 | | | | | | 2.62 | 4.07E-03 | | | |  |
| NM_201269 | | | | | | | ZNF644 | | | -3.64 | | | | | | | | | | | | | | | 8.12E-04 | | | | | | 3.23 | 4.19E-03 | | | |  |
| AK160373 | | | | | | | ZNF710 | | | -2.07 | | | | | | | | | | | | | | | 2.27E-03 | | | | | | 2.49 | 2.58E-03 | | | |  |
| NM_007131 | | | | | | | ZNF75D | | | -2.57 | | | | | | | | | | | | | | | 4.12E-04 | | | | | | 2.05 | 5.07E-03 | | | |  |
| NM_017953 | | | | | | | ZNHIT6 | | | -2.11 | | | | | | | | | | | | | | | 1.07E-03 | | | | | | 2.13 | 4.02E-03 | | | |  |
| NM_001206998 | | | | | | | ZNRF3 | | | -5.67 | | | | | | | | | | | | | | | 6.44E-04 | | | | | | 5.06 | 3.55E-03 | | | |  |
| NM_017580 | | | | | | | ZRANB1 | | | -2.11 | | | | | | | | | | | | | | | 2.86E-03 | | | | | | 2.12 | 6.74E-03 | | | |  |
| ENST00000371534 | | | | | | | ZYG11B | | | -2.80 | | | | | | | | | | | | | | | 1.46E-04 | | | | | | 2.12 | 2.57E-03 | | | |  |
| NM_015534 | | | | | | | ZZZ3 | | | -3.58 | | | | | | | | | | | | | | | 3.25E-04 | | | | | | 2.98 | 3.43E-03 | | | |  |
| 🟋 Known to be upregulated of UVB irradiation utilizing TLDA as previously reported [[1](#_ENREF_1)]  🟋 Known to be downregulated of UVB irradiation utilizing TLDA as previously reported [[1](#_ENREF_1)]  (J) Defined as the JNK-regulated genes [[2](#_ENREF_2)]  ns. Not significant  🟋 The expression level of genes in UVB-irradiated containing active photolyase samples decreased or increased to the level of control, i.e. there was no statistically significant difference comparing these samples to non-irradiated ones. | | | | | | | | | | | | | | | | | | | | | | | | | | | | | | | | | | | | |

**References**

1. Emri E, Miko E, Bai P, Boros G, Nagy G, et al. (2015) Effects of non-toxic zinc exposure on human epidermal keratinocytes. Metallomics.

2. Gazel A, Banno T, Walsh R, Blumenberg M (2006) Inhibition of JNK promotes differentiation of epidermal keratinocytes. J Biol Chem 281: 20530-20541.
