# Supplementary material for: Identification of Cyclobutane Pyrimidine Dimer-Responsive Genes Using UVB-Irradiated Human Keratinocytes Transfected with In Vitro-Synthesized Photolyase mRNA
Source: PLoS One. 2015 Jun 29;10(6):e0131141. doi: 10.1371/journal.pone.0131141 (PMC4488231; doi:10.1371/journal.pone.0131141)
Supplement: S2 Table — (DOCX) [file pone.0131141.s005.docx]

| **S2 Table. List of 76 upregulated CPD-dependent genes 24 h after UVB exposure.** | | | | | | | | | | | | | | | | | | |
| --- | --- | --- | --- | --- | --- | --- | --- | --- | --- | --- | --- | --- | --- | --- | --- | --- | --- | --- |
| **Accession Number** | | **Gene symbol** | | | **UVB vs. non-irradiated fold change** | | | | | | ***p* value** | **Active photolyase vs. inactive photolyase fold change** 🟋 | | | | |  | ***p* value** |
| NM_018394 | | | | ABHD10 | | | | 2.05 | | 6.60E-03 | | | | | -2.31 |  | ns. | |
| NM_016188 | | | | ACTL6B | | | | 2.21 | | 1.78E-02 | | | | | -2.25 |  | 3.57E-02 | |
| NM_016201 | | | | AMOTL2 | | | | 2.35 | | 1.48E-02 | | | | | -2.02 |  | ns. | |
| BC018597 | | | | ANKRD33B | | | | 2.55 | | 4.93E-03 | | | | | -2.05 |  | ns. | |
| NM_001159 | | | | AOX1 | | | | 5.72 | | 4.38E-03 | | | | | -4.05 |  | 3.99E-02 | |
| NM_025047 | | | | ARL14 | | | | 4.32 | | 2.59E-03 | | | | | -3.01 |  | 3.80E-02 | |
| NM_015459 | | | | ATL3 | | | | 2.52 | | 2.29E-03 | | | | | -2.04 |  | 3.57E-02 | |
| NM_138621 | | | | BCL2L11 | | | | 2.24 | | 4.68E-03 | | | | | -2.01 |  | 3.99E-02 | |
| BC010091 | | | | BICD1 | | | | 3.92 | | 2.29E-03 | | | | | -2.67 |  | 3.57E-02 | |
| NM_014059 | | | | C13orf15 | | | | 3.72 | | 4.38E-03 | | | | | -2.58 |  | 3.57E-02 | |
| BC003519 | | | | C14orf130 | | | | 2.27 | | 9.99E-03 | | | | | -2.29 |  | ns. | |
| BC010099 | | | | C16orf57 | | | | 2.65 | | 3.46E-03 | | | | | -2.46 |  | 3.57E-02 | |
| NM_033069 | | | | C6orf114 | | | | 3.51 | | 5.06E-03 | | | | | -2.72 |  | ns. | |
| NM_012189 | | | | CABYR | | | | 3.15 | | 3.88E-03 | | | | | -2.48 |  | 4.55E-02 | |
| NM_019044 | | | | CCDC93 | | | | 2.22 | | 1.06E-02 | | | | | -2.21 |  | 3.99E-02 | |
| NM_057749 | | | | CCNE2 | | | | 2.70 | | 7.82E-03 | | | | | -2.65 |  | ns. | |
| NM_012118 | | | | CCRN4L | | | | 4.32 | | 6.41E-03 | | | | | -2.90 |  | ns. | |
| ENST00000381577 | | | | CD274 | | | | 2.72 | | 4.46E-02 | | | | | -2.33 |  | ns. | |
| NM_000574 | | | | CD55 | | | | 2.86 | | 2.54E-03 | | | | | -2.22 |  | 3.57E-02 | |
| NM_004233 | | | | CD83 (J) | | | | 3.10 | | 9.99E-03 | | | | | -2.24 |  | ns. | |
| NM_022478 | | | | CDH24 | | | | 3.04 | | 2.29E-03 | | | | | -2.09 |  | 3.75E-02 | |
| NM_016352 | | | | CPA4 | | | | 4.84 | | 2.80E-03 | | | | | -4.12 |  | 3.57E-02 | |
| NM_000767 | | | | CYP2B6 | | | | 5.79 | | 3.54E-03 | | | | | -2.57 |  | ns. | |
| NM_139160 | | | | DEPDC7 | | | | 2.99 | | 7.82E-03 | | | | | -2.10 |  | ns. | |
| NM_004417 | | | | DUSP1 (J) | | | | 2.72 | | 1.63E-02 | | | | | -2.42 |  | ns. | |
| NM_001394 | | | | DUSP4 (J) | | | | 3.59 | | 3.22E-03 | | | | | -2.19 |  | 3.57E-02 | |
| NM_004419 | | | | DUSP5 (J) | | | | 3.20 | | 3.52E-02 | | | | | -3.04 |  | ns. | |
| NM_001955 | | | | EDN1 | | | | 6.63 | | 4.67E-02 | | | | | -4.38 |  | ns. | |
| NM_001964 | | | | EGR1 | | | | 3.38 | | 4.70E-02 | | | | | -2.39 |  | ns. | |
| NM_000399 | | | | EGR2 | | | | 8.46 | | 1.55E-02 | | | | | -5.04 |  | ns. | |
| AF099011 | | | EHD1 | | | 2.17 | | | 3.66E-02 | | | | -2.20 | | | ns. | | |
| NM_006532 | | | ELL | | | 2.09 | | | 4.67E-03 | | | | -2.04 | | | 3.57E-02 | | |
| NM_003633 | | | ENC1 (J) | | | 2.16 | | | 1.73E-02 | | | | -2.76 | | | ns. | | |
| NM_006495 | | | EVI2B | | | 2.81 | | | 2.35E-03 | | | | -2.17 | | | 3.57E-02 | | |
| BC073976 | | | FAM27A | | | 4.00 | | | 2.35E-03 | | | | -2.54 | | | 3.57E-02 | | |
| BC043603 | | | FAM44A | | | 3.11 | | | 2.29E-03 | | | | -2.20 | | | ns. | | |
| NM_005438 | | | FOSL1 | | | 2.59 | | | 4.62E-02 | | | | -2.39 | | | ns. | | |
| NM_022041 | | | GAN | | | 2.15 | | | 3.54E-03 | | | | -2.33 | | | 3.57E-02 | | |
| NM_000805 | | | GAST | | | 4.20 | | | 5.60E-03 | | | | -2.77 | | | 3.80E-02 | | |
| NM_000161 | | | GCH1 (J) | | | 2.08 | | | 4.85E-03 | | | | -2.29 | | | ns. | | |
| NM_000733 | | | GRPEL2 | | | 2.83 | | | 2.35E-03 | | | | -2.02 | | | ns. | | |
| NM_001039670 | | | IFFO1 | | | 2.92 | | | 1.33E-02 | | | | -2.34 | | | ns. | | |
| NM_000600 | | | IL6 | | | 4.47 | | | 3.54E-03 | | | | -2.41 | | | ns. | | |
| NM_005536 | | | IMPA1 | | | 2.08 | | | 1.67E-02 | | | | -2.11 | | | ns. | | |
| NM_002202 | | | ISL1 | | | 3.02 | | | 1.09E-02 | | | | -2.18 | | | ns. | | |
| NM_002228 | | | JUN 🟋(J) | | | 5.72 | | | 1.78E-02 | | | | -3.35 | | | ns. | | |
| NM_032464 | | | LAT2 | | | 4.97 | | | 6.04E-03 | | | | -2.84 | | | ns. | | |
| NM_014572 | | | LATS2 | | | 2.19 | | | 7.17E-03 | | | | -2.35 | | | 4.26E-02 | | |
| NM_033514 | | | LIMS3 | | | 3.59 | | | 2.29E-03 | | | | -2.82 | | | 3.57E-02 | | |
| NM_025261 | | | LY6G6C | | | 3.55 | | | 3.40E-02 | | | | -2.15 | | | ns. | | |
| NM_032793 | | | MFSD2A | | | 2.94 | | | 4.54E-02 | | | | -2.38 | | | ns. | | |
| DQ080207 | | | MIG7 | | | 6.68 | | | 2.29E-03 | | | | -4.07 | | | 3.57E-02 | | |
| NM_198159 | | | MITF | | | 3.26 | | | 3.25E-03 | | | | -2.05 | | | 4.78E-02 | | |
| NM_138768 | | | MYEOV | | | 2.19 | | | 4.93E-03 | | | | -2.04 | | | 4.02E-02 | | |
| NM_144599 | | | NIPA1 | | | 2.00 | | | 1.19E-02 | | | | -2.20 | | | 3.57E-02 | | |
| NM_002135 | | | NR4A1 | | | 4.02 | | | 1.26E-02 | | | | -2.75 | | | ns. | | |
| NM_024576 | | | OGFRL1 | | | 3.05 | | | 2.29E-03 | | | | -2.09 | | | 3.57E-02 | | |
| NM_032521 | | | PARD6B | | | 2.29 | | | 6.09E-03 | | | | -2.03 | | | 4.15E-02 | | |
| NM_002638 | | | PI3 (J) | | | 4.22 | | | 5.61E-03 | | | | -4.77 | | | 3.57E-02 | | |
| NM_152542 | | | PPM1K | | | 3.56 | | | 2.48E-03 | | | | -2.69 | | | 3.57E-02 | | |
| NM_000963 | | | PTGS2 🟋(J) | | | 4.66 | | | ns. | | | | -3.04 | | | ns. | | |
| NM_016084 | | | RASD1 | | | 3.10 | | | 1.73E-02 | | | | -2.98 | | | ns. | | |
| NM_004636 | | | SEMA3B | | | 2.31 | | | 3.36E-02 | | | | -2.11 | | | ns. | | |
| NM_152621 | | | SGMS2 | | | 2.28 | | | 1.75E-02 | | | | | -2.37 | | ns. | | |
| NM_001042496 | | | SLC12A6 | | | 2.92 | | | 2.29E-03 | | | | | -2.77 | | 3.57E-02 | | |
| NM_005985 | | | SNAI1 | | | 2.76 | | | 6.60E-03 | | | | | -2.59 | | 3.94E-02 | | |
| NM_014598 | | | SOCS7 | | | 3.26 | | | 4.28E-03 | | | | | -2.33 | | ns. | | |
| NM_003114 | | | SPAG1 (J) | | | 2.30 | | | 2.85E-02 | | | | | -2.40 | | 4.43E-02 | | |
| NM_001112724 | | | STK32A | | | 2.93 | | | 4.74E-02 | | | | | -2.02 | | ns. | | |
| NM_015000 | | | STK38L | | | 2.55 | | | 2.29E-03 | | | | | -2.15 | | 3.57E-02 | | |
| ENST00000222543 | | | TFPI2 | | | 5.07 | | | 4.38E-03 | | | | | -2.61 | | ns. | | |
| BC005335 | | | TMEM87A | | | 2.34 | | | 4.02E-03 | | | | | -2.14 | | 3.88E-02 | | |
| NM_006290 | | | TNFAIP3 🟋(J) | | | 3.64 | | | 1.77E-02 | | | | | -3.16 | | ns. | | |
| NM_003324 | | | TULP3 | | | 2.59 | | | 2.29E-03 | | | | | -2.59 | | 3.57E-02 | | |
| NM_017853 | | | TXNL4B | | | 2.03 | | | 1.95E-02 | | | | | -2.03 | | ns. | | |
| NM_014797 | | | ZBTB24 | | | 2.57 | | | 1.00E-02 | | | | | -2.01 | | 4.41E-02 | | |
| **List of 250 downregulated CPD-dependent genes 24 h after UVB exposure.** | | | | | | | | | | | | | | | | | | |
| NM_003786 | | | | ABCC3 | | | -3.02 | | 2.29E-03 | | | | | 2.65 | | 3.57E-02 | | |
| NM_138448 | | | | ACYP2 | | | -12.61 | | 1.37E-02 | | | | | 9.93 | | ns. | | |
| NM_139025 | | | | ADAMTS13 | | | -2.82 | | 3.06E-03 | | | | | 2.21 | | ns. | | |
| NM_001116 | | | | ADCY9 | | | -2.50 | | 1.60E-02 | | | | | 2.58 | | ns. | | |
| NM_016824 | | | | ADD3 | | | -2.26 | | 9.46E-03 | | | | | 2.06 | | 3.80E-02 | | |
| NM_001123 | | | | ADK | | | -3.92 | | 5.70E-03 | | | | | 3.51 | | 3.75E-02 | | |
| NM_001037131 | | | | AGAP1 | | | -4.47 | | 4.68E-03 | | | | | 3.25 | | 3.76E-02 | | |
| NM_024060 | | | | AHNAK | | | -4.47 | | 2.54E-03 | | | | | 2.94 | | 3.57E-02 | | |
| NM_016108 | | | | AIG1 | | | -3.34 | | 4.37E-03 | | | | | 3.08 | | 3.57E-02 | | |
| NM_005465 | | | | AKT3 | | | -3.06 | | 9.71E-03 | | | | | 3.35 | | 3.99E-02 | | |
| NM_000693 | | | | ALDH1A3 (J) | | | -2.35 | | 1.27E-02 | | | | | 2.57 | | 3.99E-02 | | |
| NM_001031806 | | | | ALDH3A2 | | | -2.71 | | 3.75E-02 | | | | | 2.16 | | ns. | | |
| NM_144988 | | | | ALG14 | | | -2.25 | | 7.17E-03 | | | | | 2.55 | | 3.99E-02 | | |
| NM_025144 | | | | ALPK1 | | | -9.42 | | 1.56E-02 | | | | | 6.84 | | ns. | | |
| THC2566704 | | | | ALU8 | | | -3.66 | | 3.48E-03 | | | | | 2.60 | | 3.80E-02 | | |
| NM_020987 | | | | ANK3 | | | -9.04 | | 4.17E-03 | | | | | 8.06 | | 3.57E-02 | | |
| NM_032208 | | | | ANTXR1 | | | -3.79 | | 3.07E-02 | | | | | 4.32 | | ns. | | |
| NM_173075 | | | | APBB2 | | | -2.85 | | 1.76E-02 | | | | | 3.19 | | ns. | | |
| NM_017519 | | | | ARID1B | | | -2.84 | | 1.69E-02 | | | | | 2.66 | | ns. | | |
| NM_000050 | | | | ASS1 | | | -2.82 | | 1.87E-02 | | | | | 2.00 | | ns. | | |
| NM_006045 | | | | ATP9A | | | -2.70 | | 4.22E-02 | | | | | 2.32 | | ns. | | |
| NM_198531 | | | | ATP9B | | | -2.72 | | 2.93E-03 | | | | | 2.59 | | 3.57E-02 | | |
| NM_139321 | | | | ATRN | | | -2.05 | | 5.20E-03 | | | | | 2.10 | | 3.57E-02 | | |
| NM_020371 | | | | AVEN | | | -2.10 | | 9.00E-03 | | | | | 2.16 | | 4.51E-02 | | |
| NM_017679 | | | | BCAS3 | | | -11.19 | | 2.29E-03 | | | | | 6.99 | | 3.57E-02 | | |
| NM_005504 | | | | BCAT1 | | | -2.31 | | 2.76E-02 | | | | | 2.88 | | ns. | | |
| NM_000056 | | | | BCKDHB | | | -2.49 | | 3.99E-02 | | | | | 2.52 | | ns. | | |
| NM_004899 | | | | BRE | | | -5.23 | | 2.35E-03 | | | | | 3.62 | | 3.84E-02 | | |
| NM_052893 | | | | BTBD9 | | | -9.31 | | 7.23E-03 | | | | | 8.14 | | 3.99E-02 | | |
| NM_001003676 | | | | C11orf49 | | | -3.45 | | 9.71E-03 | | | | | 3.01 | | ns. | | |
| NM_152446 | | | | C14orf145 | | | -3.38 | | 9.32E-03 | | | | | 4.00 | | 3.57E-02 | | |
| NM_024952 | | | | C14orf159 | | | -3.66 | | 1.36E-02 | | | | | 2.73 | | ns. | | |
| NM_032359 | | | | C3orf26 | | | -2.19 | | 1.60E-02 | | | | | 2.65 | | ns. | | |
| NM_145814 | | | | CACNG6 | | | -3.35 | | 3.73E-03 | | | | | 2.35 | | 4.69E-02 | | |
| NM_172127 | | | | CAMK2D | | | -2.49 | | 1.59E-02 | | | | | 2.40 | | 4.90E-02 | | |
| NM_003688 | | | | CASK | | | -4.51 | | 2.46E-02 | | | | | 4.85 | | ns. | | |
| NM_175709 | | | | CBX7 | | | -2.14 | | 1.16E-02 | | | | | 2.01 | | ns. | | |
| NM_018318 | | | | CCDC91 | | | -2.36 | | 1.74E-02 | | | | | 2.68 | | ns. | | |
| NM_006584 | | | | CCT6B | | | -2.40 | | 7.82E-03 | | | | | 2.07 | | ns. | | |
| NM_003607 | | | | CDC42BPA | | | -2.74 | | 3.65E-03 | | | | | 2.13 | | 3.99E-02 | | |
| NM_001257 | | | | CDH13 | | | -17.58 | | 1.87E-02 | | | | | 11.13 | | ns. | | |
| ENST00000378610 | | | | CDKAL1 | | | -9.15 | | 9.22E-03 | | | | | 6.25 | | ns. | | |
| NM_014246 | | | | CELSR1 | | | -3.30 | | 4.37E-03 | | | | | 2.30 | | 4.70E-02 | | |
| NM_001012267 | | | | CENPP | | | -2.85 | | 1.60E-02 | | | | | 2.17 | | ns. | | |
| NM_000186 | | | | CFH | | | -3.16 | | 4.64E-02 | | | | | 3.17 | | ns. | | |
| NM_006536 | | | | CLCA2 | | | -3.87 | | 2.16E-02 | | | | | 2.08 | | ns. | | |
| NM_003388 | | | | CLIP2 | | | -2.38 | | 2.73E-02 | | | | | 2.14 | | ns. | | |
| BX648591 | | | | CNTN1 | | | -6.27 | | 9.90E-03 | | | | | 5.85 | | 4.50E-02 | | |
| NM_030582 | | | | COL18A1 | | | -2.62 | | 2.62E-02 | | | | | 2.61 | | 4.77E-02 | | |
| NM_033380 | | | | COL4A5 | | | -3.14 | | 1.60E-02 | | | | | 3.10 | | ns. | | |
| NM_033641 | | | | COL4A6 | | | -5.55 | | 1.86E-02 | | | | | 5.51 | | ns. | | |
| NM_152516 | | | | COMMD1 | | | -5.74 | | 2.69E-03 | | | | | 4.37 | | 3.57E-02 | | |
| NM_016144 | | | | COMMD10 | | | -6.43 | | 4.65E-03 | | | | | 5.71 | | 3.80E-02 | | |
| NM_003805 | | | | CRADD | | | -4.01 | | 2.58E-03 | | | | | 3.55 | | 3.57E-02 | | |
| NM_015974 | | | | CRYL1 | | | -2.44 | | 7.63E-03 | | | | | 2.02 | | ns. | | |
| NM_015089 | | | | CUL9 | | | -2.58 | | 8.39E-03 | | | | | 2.39 | | 4.50E-02 | | |
| NM_000104 | | | | CYP1B1 (J) | | | -2.46 | | 3.97E-02 | | | | | 6.01 | | 3.57E-02 | | |
| NM_207352 | | | | CYP4V2 | | | -2.43 | | 3.48E-03 | | | | | 2.14 | | 3.99E-02 | | |
| NM_020946 | | | | DENND1A | | | -15.73 | | 1.07E-02 | | | | | 14.54 | | 4.70E-02 | | |
| NM_017996 | | | | DET1 | | | -2.55 | | 4.78E-03 | | | | | 2.08 | | ns. | | |
| NM_145177 | | | | DHRSX | | | -6.64 | | 3.65E-03 | | | | | 6.43 | | 3.57E-02 | | |
| NM_006729 | | | | DIAPH2 | | | -19.41 | | 4.66E-03 | | | | | 19.16 | | 3.61E-02 | | |
| NM_001042517 | | | | DIAPH3 | | | -2.79 | | 4.28E-03 | | | | | 2.59 | | 3.99E-02 | | |
| NM_152383 | | | | DIS3L2 | | | -4.00 | | 4.85E-03 | | | | | 3.82 | | ns. | | |
| NM_175850 | | | | DNMT3B | | | -2.51 | | 3.22E-03 | | | | | 2.23 | | 3.57E-02 | | |
| NM_001380 | | | | DOCK1 | | | -5.61 | | 4.38E-03 | | | | | 5.37 | | 3.57E-02 | | |
| NM_000110 | | | | DPYD | | | -9.41 | | 2.29E-03 | | | | | 8.15 | | 3.57E-02 | | |
| NM_080820 | | | | DTD1 | | | -2.64 | | 4.66E-03 | | | | | 2.38 | | ns. | | |
| NM_183360 | | | | DTNB | | | -3.32 | | 3.22E-03 | | | | | 2.92 | | 3.57E-02 | | |
| NM_017653 | | | | DYM | | | -2.51 | | 1.08E-02 | | | | | 3.15 | | ns. | | |
| NM_014648 | | | | DZIP3 | | | -2.36 | | 1.36E-02 | | | | | 2.12 | | 4.51E-02 | | |
| NM_005711 | | | | EDIL3 | | | -5.88 | | 7.90E-03 | | | | | 5.17 | | 3.76E-02 | | |
| NM_032328 | | | | EFCAB2 | | | -2.17 | | 3.49E-02 | | | | | 2.39 | | ns. | | |
| NM_019040 | | | | ELP4 | | | -2.30 | | 2.35E-02 | | | | | 2.50 | | 4.88E-02 | | |
| NM_182314 | | | | ENOX2 | | | -2.51 | | 3.48E-03 | | | | | 2.39 | | 3.57E-02 | | |
| NM_004442 | | | | EPHB2 | | | -3.10 | | 1.78E-02 | | | | | 2.44 | | ns. | | |
| NM_178037 | | | | ERC1 | | | -3.27 | | 8.00E-03 | | | | | 2.52 | | 4.51E-02 | | |
| NM_021807 | | | | EXOC4 | | | -18.22 | | 3.20E-03 | | | | | 11.21 | | 3.99E-02 | | |
| ENST00000272427 | | | | EXOC6B | | | -5.66 | | 3.88E-03 | | | | | 3.72 | | ns. | | |
| NM_007051 | | | | FAF1 | | | -2.50 | | 4.08E-03 | | | | | 2.24 | | 3.75E-02 | | |
| NM_017848 | | | | FAM120C | | | -3.36 | | 1.27E-02 | | | | | 3.71 | | ns. | | |
| ENST00000372955 | | | | FAM149B1 | | | -2.15 | | 5.17E-03 | | | | | 2.18 | | ns. | | |
| NM_032042 | | | | FAM172A | | | -9.23 | | 4.08E-03 | | | | | 6.40 | | ns. | | |
| NM_030797 | | | | FAM49A | | | -4.74 | | 4.08E-03 | | | | | 3.80 | | 3.75E-02 | | |
| NM_000136 | | | | FANCC | | | -2.31 | | 4.02E-03 | | | | | 2.24 | | 3.57E-02 | | |
| NM_006567 | | | | FARS2 | | | -2.88 | | 3.48E-03 | | | | | 2.52 | | 4.11E-02 | | |
| NM_001447 | | | | FAT2 | | | -3.84 | | 2.90E-02 | | | | | 2.44 | | ns. | | |
| NM_006486 | | | | FBLN1 | | | -2.21 | | 2.17E-02 | | | | | 2.18 | | 3.99E-02 | | |
| NM_152676 | | | | FBXO15 | | | -4.42 | | 2.29E-03 | | | | | 3.05 | | ns. | | |
| NM_031456 | | | | FBXW10 | | | -2.66 | | 1.97E-02 | | | | | 3.01 | | 4.51E-02 | | |
| ENST00000281092 | | | | FER | | | -2.84 | | 2.47E-02 | | | | | 3.19 | | ns. | | |
| NM_018291 | | | | FGGY | | | -12.82 | | 3.61E-03 | | | | | 8.80 | | 3.57E-02 | | |
| NM_025135 | | | | FHOD3 | | | -2.48 | | 1.94E-02 | | | | | 2.82 | | ns. | | |
| NM_033260 | | | | FOXQ1 | | | -2.67 | | 1.77E-02 | | | | | 2.61 | | 3.57E-02 | | |
| BC003583 | | | | FTO | | | -3.90 | | 4.68E-03 | | | | | 3.51 | | 3.61E-02 | | |
| NM_198516 | | | | GALNTL4 | | | -2.39 | | 9.03E-03 | | | | | 2.48 | | 4.07E-02 | | |
| NM_001500 | | | | GMDS | | | -4.84 | | 2.29E-03 | | | | | 3.97 | | 3.57E-02 | | |
| NM_020806 | | | | GPHN | | | -4.40 | | 6.02E-03 | | | | | 3.51 | | ns. | | |
| NM_153840 | | | | GPR110 | | | -2.37 | | 1.50E-02 | | | | | 2.16 | | 4.43E-02 | | |
| NM_001006636 | | | | GTDC1 | | | -12.72 | | 3.10E-03 | | | | | 10.60 | | 3.57E-02 | | |
| NM_016315 | | | | GULP1 | | | -4.62 | | 3.54E-02 | | | | | 5.35 | | ns. | | |
| NM_006037 | | | | HDAC4 | | | -4.25 | | 1.04E-02 | | | | | 4.35 | | 4.50E-02 | | |
| NM_020733 | | | | HEG1 | | | -2.05 | | 2.34E-02 | | | | | 2.43 | | 4.77E-02 | | |
| ENST00000336926 | | | | HIP1 | | | -5.26 | | 1.96E-02 | | | | | 3.11 | | ns. | | |
| ENST00000314088 | | | | HIST1H2AC | | | -4.67 | | 4.37E-03 | | | | | 2.40 | | ns. | | |
| NM_000411 | | | | HLCS | | | -2.55 | | 8.75E-03 | | | | | 2.72 | | 4.90E-02 | | |
| NM_000860 | | | | HPGD | | | -3.15 | | 1.25E-02 | | | | | 2.20 | | 4.55E-02 | | |
| NM_002165 | | | | ID1 | | | -3.31 | | 2.23E-02 | | | | | 2.41 | | ns. | | |
| NM_002167 | | | | ID3 | | | -2.90 | | 8.94E-03 | | | | | 2.07 | | ns. | | |
| NM_032549 | | | | IMMP2L | | | -37.16 | | 3.54E-03 | | | | | 24.69 | | 3.57E-02 | | |
| NM_015075 | | | | IQSEC2 | | | -2.69 | | 1.28E-02 | | | | | 2.91 | | ns. | | |
| NM_030790 | | | | ITFG1 | | | -2.91 | | 5.75E-03 | | | | | 2.58 | | 4.67E-02 | | |
| NM_004791 | | | | ITGBL1 | | | -2.79 | | 9.22E-03 | | | | | 3.26 | | 3.80E-02 | | |
| NM_002237 | | | | KCNG1 | | | -2.33 | | 3.45E-02 | | | | | 2.12 | | ns. | | |
| NM_015061 | | | | KDM4C | | | -3.41 | | 1.51E-02 | | | | | 3.16 | | ns. | | |
| NM_020817 | | | | KIAA1407 | | | -3.24 | | 3.65E-03 | | | | | 2.74 | | 3.57E-02 | | |
| NM_017794 | | | | KIAA1797 | | | -3.94 | | 4.38E-03 | | | | | 3.95 | | 3.80E-02 | | |
| NM_024704 | | | | KIF16B | | | -3.73 | | 1.08E-02 | | | | | 3.73 | | ns. | | |
| NM_007249 | | | | KLF12 | | | -3.23 | | 1.56E-02 | | | | | 3.02 | | ns. | | |
| NM_002274 | | | | KRT13 (J) | | | -7.24 | | 2.12E-02 | | | | | 3.15 | | ns. | | |
| NM_002275 | | | | KRT15 | | | -7.24 | | 3.95E-02 | | | | | 3.16 | | ns. | | |
| NM_002294 | | | | LAMP2 | | | -2.27 | | 2.15E-02 | | | | | 2.40 | | ns. | | |
| NM_203463 | | | | LASS6 | | | -2.37 | | 1.16E-02 | | | | | 2.84 | | 4.28E-02 | | |
| NM_022126 | | | | LHPP | | | -3.71 | | 5.68E-03 | | | | | 3.30 | | 3.70E-02 | | |
| NM_005578 | | | | LPP | | | -5.71 | | 4.93E-03 | | | | | 5.94 | | 3.61E-02 | | |
| NM_006726 | | | | LRBA | | | -6.03 | | 3.65E-03 | | | | | 5.63 | | 3.57E-02 | | |
| NM_133259 | | | | LRPPRC | | | -2.04 | | 1.69E-02 | | | | | 2.27 | | 4.51E-02 | | |
| NM_001009909 | | | | LUZP2 | | | -4.30 | | 2.65E-02 | | | | | 4.51 | | ns. | | |
| NM_003550 | | | | MAD1L1 | | | -2.54 | | 2.12E-02 | | | | | 2.23 | | ns. | | |
| NM_002757 | | | | MAP2K5 | | | -4.18 | | 3.22E-03 | | | | | 3.49 | | 3.57E-02 | | |
| NM_030583 | | | | MATN2 | | | -3.07 | | 4.75E-03 | | | | | 2.19 | | ns. | | |
| CR627122 | | | | MBNL3 | | | -2.79 | | 7.77E-03 | | | | | 2.62 | | 3.94E-02 | | |
| NM_002397 | | | | MEF2C | | | -7.67 | | 4.08E-03 | | | | | 5.58 | | 4.03E-02 | | |
| NM_178121 | | | | MEGF8 | | | -2.25 | | 5.61E-03 | | | | | 2.38 | | 3.99E-02 | | |
| NM_024302 | | | | MMP28 | | | -3.52 | | 4.28E-02 | | | | | 2.50 | | ns. | | |
| NM_173496 | | | | MPP7 | | | -2.11 | | 4.60E-02 | | | | | 2.53 | | ns. | | |
| NM_001584 | | | | MPPED2 | | | -2.31 | | 1.86E-02 | | | | | 2.23 | | 4.24E-02 | | |
| NM_012331 | | | | MSRA | | | -12.68 | | 2.35E-03 | | | | | 9.50 | | 3.57E-02 | | |
| NM_012228 | | | | MSRB2 | | | -3.24 | | 5.70E-03 | | | | | 2.29 | | ns. | | |
| ENST00000405592 | | | | MTA3 | | | -3.23 | | 5.40E-03 | | | | | 2.94 | | ns. | | |
| NM_152673 | | | | MUC20 | | | -2.13 | | 2.04E-02 | | | | | 2.05 | | ns. | | |
| NM_053025 | | | | MYLK | | | -4.40 | | 1.12E-02 | | | | | 3.36 | | ns. | | |
| NM_006901 | | | | MYO9A | | | -2.88 | | 1.19E-02 | | | | | 2.98 | | ns. | | |
| NM_015909 | | | | NBAS | | | -5.36 | | 2.29E-03 | | | | | 4.03 | | 3.57E-02 | | |
| NM_015678 | | | | NBEA | | | -21.54 | | 2.93E-03 | | | | | 20.57 | | 3.57E-02 | | |
| NM_147233 | | | | NCOA1 | | | -4.15 | | 8.30E-03 | | | | | 3.04 | | 4.88E-02 | | |
| NM_181722 | | | | NCRNA00174 | | | -2.28 | | 2.35E-03 | | | | | 2.27 | | 3.57E-02 | | |
| NM_006393 | | | | NEBL | | | -2.45 | | 2.35E-02 | | | | | 2.36 | | ns. | | |
| NM_002499 | | | | NEO1 | | | -5.05 | | 2.53E-03 | | | | | 3.60 | | ns. | | |
| NM_001142651 | | | | NEURL1B | | | -9.44 | | 9.13E-03 | | | | | 3.40 | | ns. | | |
| NM_005596 | | | | NFIB | | | -2.57 | | 9.00E-03 | | | | | 2.61 | | 4.70E-02 | | |
| NM_024759 | | | | NIPAL2 | | | -3.15 | | 7.90E-03 | | | | | 3.34 | | 3.57E-02 | | |
| NM_013330 | | | | NME7 | | | -2.35 | | 1.27E-02 | | | | | 2.44 | | 4.55E-02 | | |
| BC073815 | | | | NPB | | | -2.15 | | 2.29E-02 | | | | | 2.12 | | ns. | | |
| NM_000272 | | | | NPHP1 | | | -2.09 | | 8.46E-03 | | | | | 2.04 | | 3.57E-02 | | |
| NM_021229 | | | | NTN4 | | | -2.15 | | 4.59E-02 | | | | | 2.02 | | ns. | | |
| NM_025152 | | | | NUBPL | | | -3.37 | | 1.16E-02 | | | | | 2.21 | | ns. | | |
| NM_001042483 | | | | NUPR1 | | | -2.79 | | 3.46E-03 | | | | | 2.46 | | 3.57E-02 | | |
| NM_001080507 | | | | OOEP | | | -2.53 | | 3.75E-02 | | | | | 2.93 | | ns. | | |
| NM_002547 | | | | OPHN1 | | | -4.74 | | 1.03E-02 | | | | | 4.96 | | ns. | | |
| NM_145047 | | | | OSCP1 | | | -2.08 | | 6.41E-03 | | | | | 2.05 | | 4.69E-02 | | |
| AY320284 | | | | PACS2 | | | -2.25 | | 2.04E-02 | | | | | 2.45 | | 4.28E-02 | | |
| NM_000919 | | | | PAM | | | -2.84 | | 4.91E-03 | | | | | 2.54 | | 3.57E-02 | | |
| NM_019619 | | | | PARD3 | | | -4.05 | | 7.08E-03 | | | | | 4.04 | | 3.75E-02 | | |
| AK128814 | | | | PARVA | | | -2.61 | | 1.05E-02 | | | | | 3.24 | | 4.03E-02 | | |
| NM_002585 | | | | PBX1 | | | -5.24 | | 1.71E-02 | | | | | 4.74 | | ns. | | |
| NM_000282 | | | | PCCA | | | -8.88 | | 4.37E-03 | | | | | 8.22 | | 3.80E-02 | | |
| NM_014801 | | | | PCNXL2 | | | -2.34 | | 4.68E-03 | | | | | 2.35 | | 3.57E-02 | | |
| NM_020381 | | | | PDSS2 | | | -5.13 | | 4.66E-03 | | | | | 4.79 | | 3.76E-02 | | |
| NM_003846 | | | | PEX11B | | | -2.19 | | 3.51E-02 | | | | | 2.23 | | ns. | | |
| NM_001031835 | | | | PHKB | | | -2.73 | | 1.60E-02 | | | | | 2.47 | | ns. | | |
| NM_025049 | | | | PIF1 | | | -2.44 | | 3.00E-03 | | | | | 2.32 | | 3.57E-02 | | |
| NM_003662 | | | | PIR | | | -2.27 | | 4.06E-02 | | | | | 2.58 | | ns. | | |
| NM_181671 | | | | PITPNC1 | | | -3.37 | | 2.80E-03 | | | | | 2.53 | | ns. | | |
| NM_181805 | | | | PKIG | | | -3.05 | | 2.29E-03 | | | | | 2.32 | | 3.57E-02 | | |
| NM_002662 | | | | PLD1 | | | -3.20 | | 1.27E-02 | | | | | 3.09 | | ns. | | |
| NM_032812 | | | | PLXDC2 | | | -7.95 | | 4.02E-03 | | | | | 6.55 | | 3.57E-02 | | |
| NM_003621 | | | | PPFIBP2 | | | -3.95 | | 4.55E-03 | | | | | 2.98 | | 3.76E-02 | | |
| NM_005400 | | | | PRKCE | | | -3.97 | | 1.87E-02 | | | | | 3.43 | | 4.88E-02 | | |
| NM_001098538 | | | | PRR4 | | | -2.05 | | 7.90E-03 | | | | | 2.01 | | 3.57E-02 | | |
| NM_015310 | | | | PSD3 | | | -3.09 | | 3.22E-03 | | | | | 3.58 | | 4.03E-02 | | |
| NM_001145369 | | | | PTPN3 | | | -2.08 | | 3.77E-02 | | | | | 2.15 | | ns. | | |
| NM_032932 | | | | RAB11FIP4 | | | -2.94 | | 2.44E-02 | | | | | 2.16 | | ns. | | |
| NM_014857 | | | | RABGAP1L | | | -7.89 | | 9.01E-03 | | | | | 7.46 | | 4.50E-02 | | |
| NM_133510 | | | | RAD51L1 | | | -7.19 | | 2.44E-03 | | | | | 4.90 | | ns. | | |
| NM_006989 | | | | RASA4 | | | -3.59 | | 2.33E-02 | | | | | 2.81 | | ns. | | |
| NM_022128 | | | | RBKS | | | -2.64 | | 8.97E-03 | | | | | 2.22 | | 4.99E-02 | | |
| NM_004726 | | | | REPS2 | | | -2.77 | | 2.79E-02 | | | | | 2.78 | | ns. | | |
| NM_032276 | | | | RHBDD1 | | | -2.61 | | 3.48E-03 | | | | | 2.54 | | 4.06E-02 | | |
| NM_133631 | | | | ROBO1 | | | -3.32 | | 1.16E-02 | | | | | 3.18 | | ns. | | |
| NM_005012 | | | | ROR1 | | | -2.66 | | 1.01E-02 | | | | | 2.95 | | 3.88E-02 | | |
| NM_021135 | | | | RPS6KA2 | | | -2.29 | | 1.60E-02 | | | | | 2.22 | | ns. | | |
| NM_016625 | | | | RSRC1 | | | -2.95 | | 2.06E-02 | | | | | 3.26 | | 3.94E-02 | | |
| NM_012425 | | | | RSU1 | | | -3.09 | | 3.00E-03 | | | | | 2.68 | | 3.57E-02 | | |
| NM_024632 | | | | SAP30L | | | -2.80 | | 6.33E-03 | | | | | 2.07 | | ns. | | |
| NM_030962 | | | | SBF2 | | | -2.37 | | 3.40E-02 | | | | | 2.15 | | ns. | | |
| NM_152540 | | | | SCFD2 | | | -8.45 | | 7.84E-03 | | | | | 7.59 | | ns. | | |
| NM_006642 | | | | SDCCAG8 | | | -2.43 | | 4.85E-03 | | | | | 2.30 | | 3.57E-02 | | |
| NM_012139 | | | | SERGEF | | | -3.81 | | 1.72E-02 | | | | | 3.70 | | ns. | | |
| NM_012397 | | | | SERPINB13 | | | -3.25 | | 2.29E-03 | | | | | 2.79 | | 3.57E-02 | | |
| NM_015559 | | | | SETBP1 | | | -3.14 | | 8.00E-03 | | | | | 4.07 | | 4.55E-02 | | |
| NM_024776 | | | | SGK269 | | | -2.51 | | 1.65E-02 | | | | | 2.37 | | ns. | | |
| NM_014631 | | | | SH3PXD2A | | | -2.57 | | 9.03E-03 | | | | | 2.30 | | ns. | | |
| NM_001037633 | | | | SIL1 | | | -2.95 | | 2.53E-03 | | | | | 2.52 | | 3.76E-02 | | |
| NM_015556 | | | | SIPA1L1 | | | -2.53 | | 4.99E-03 | | | | | 2.02 | | 3.57E-02 | | |
| NM_015073 | | | | SIPA1L3 | | | -2.41 | | 2.29E-03 | | | | | 2.09 | | ns. | | |
| NM_015482 | | | | SLC22A23 | | | -3.66 | | 9.25E-03 | | | | | 3.37 | | ns. | | |
| NM_198580 | | | | SLC27A1 | | | -2.13 | | 4.56E-02 | | | | | 2.09 | | ns. | | |
| NM_001001290 | | | | SLC2A9 | | | -2.82 | | 2.37E-02 | | | | | 2.62 | | ns. | | |
| NM_139177 | | | | SLC39A11 | | | -10.26 | | 6.60E-03 | | | | | 7.76 | | 4.09E-02 | | |
| NM_003486 | | | | SLC7A5 | | | -2.87 | | 9.65E-03 | | | | | 2.19 | | 4.03E-02 | | |
| NR_024054 | | | | SMA4 | | | -2.92 | | 1.91E-02 | | | | | 3.25 | | 4.69E-02 | | |
| NM_022743 | | | | SMYD3 | | | -16.09 | | 3.25E-03 | | | | | 13.29 | | 3.57E-02 | | |
| NM_014390 | | | | SND1 | | | -2.63 | | 5.70E-03 | | | | | 2.48 | | 3.99E-02 | | |
| NM_032167 | | | | SNX29 | | | -4.17 | | 6.73E-03 | | | | | 4.02 | | 3.88E-02 | | |
| NM_003105 | | | | SORL1 | | | -2.56 | | 1.47E-02 | | | | | 2.25 | | ns. | | |
| NM_002959 | | | | SORT1 | | | -2.10 | | 4.91E-03 | | | | | 2.33 | | ns. | | |
| NM_001080394 | | | | SPIDR | | | -5.26 | | 4.04E-03 | | | | | 4.68 | | 3.57E-02 | | |
| NM_005862 | | | | STAG1 | | | -2.28 | | 1.50E-02 | | | | | 2.06 | | ns. | | |
| NM_001164380 | | | | STAU2 | | | -2.54 | | 7.82E-03 | | | | | 2.21 | | 3.57E-02 | | |
| NM_004853 | | | | STX8 | | | -9.87 | | 3.00E-03 | | | | | 5.76 | | ns. | | |
| NM_182760 | | | | SUMF1 | | | -3.82 | | 2.58E-03 | | | | | 3.00 | | 3.57E-02 | | |
| NM_032872 | | | | SYTL1 | | | -3.34 | | 1.93E-02 | | | | | 2.30 | | ns. | | |
| NM_014346 | | | | TBC1D22A | | | -2.18 | | 4.93E-03 | | | | | 2.03 | | 3.99E-02 | | |
| NM_014744 | | | | TBC1D5 | | | -19.96 | | 2.43E-03 | | | | | 13.10 | | 3.57E-02 | | |
| NM_033115 | | | | TBCK | | | -3.18 | | 6.22E-03 | | | | | 2.10 | | ns. | | |
| NM_005647 | | | | TBL1X | | | -2.54 | | 4.53E-02 | | | | | 2.00 | | ns. | | |
| ENST00000267811 | | | | TCF12 | | | -2.72 | | 4.92E-03 | | | | | 2.24 | | ns. | | |
| NM_003199 | | | | TCF4 | | | -2.44 | | 2.29E-03 | | | | | 2.62 | | 3.57E-02 | | |
| NM_006287 | | | | TFPI | | | -2.81 | | 6.33E-03 | | | | | 2.81 | | 3.57E-02 | | |
| NM_000361 | | | | THBD | | | -2.92 | | 3.95E-03 | | | | | 2.85 | | ns. | | |
| NM_024817 | | | | THSD4 | | | -3.55 | | 2.57E-02 | | | | | 3.90 | | ns. | | |
| NM_032256 | | | | TMEM117 | | | -6.04 | | 3.05E-03 | | | | | 5.70 | | 3.57E-02 | | |
| NM_152588 | | | | TMTC2 | | | -9.87 | | 3.65E-03 | | | | | 7.81 | | 3.57E-02 | | |
| NM_015028 | | | | TNIK | | | -2.07 | | 6.70E-03 | | | | | 2.09 | | ns. | | |
| NM_031466 | | | | TRAPPC9 | | | -9.81 | | 2.29E-03 | | | | | 6.96 | | 3.57E-02 | | |
| NM_014818 | | | | TRIM66 | | | -2.07 | | 2.69E-02 | | | | | 2.38 | | ns. | | |
| NM_152653 | | | | UBE2E2 | | | -3.46 | | 3.46E-03 | | | | | 2.80 | | ns. | | |
| NM_001072 | | | | UGT1A6 | | | -2.19 | | 1.12E-02 | | | | | 2.31 | | 4.17E-02 | | |
| NM_007124 | | | | UTRN | | | -4.81 | | 8.30E-03 | | | | | 4.70 | | 3.99E-02 | | |
| NM_017890 | | | | VPS13B | | | -6.52 | | 5.21E-03 | | | | | 7.00 | | 3.57E-02 | | |
| NM_020821 | | | | VPS13C | | | -2.01 | | 7.96E-03 | | | | | 2.08 | | 3.57E-02 | | |
| NM_014396 | | | | VPS41 | | | -2.07 | | 3.39E-02 | | | | | 2.34 | | 4.70E-02 | | |
| NM_001009921 | | | | VPS8 | | | -3.25 | | 2.35E-03 | | | | | 2.42 | | ns. | | |
| NM_145206 | | | | VTI1A | | | -4.00 | | 3.48E-03 | | | | | 3.88 | | 3.57E-02 | | |
| NM_015285 | | | | WDR7 | | | -3.76 | | 8.00E-03 | | | | | 3.95 | | 3.80E-02 | | |
| NM_016373 | | | | WWOX | | | -3.27 | | 2.46E-02 | | | | | 2.21 | | ns. | | |
| NM_022550 | | | | XRCC4 | | | -2.30 | | 1.70E-02 | | | | | 2.29 | | 4.90E-02 | | |
| NM_153746 | | | | ZDHHC14 | | | -4.28 | | 1.03E-02 | | | | | 5.09 | | 4.03E-02 | | |
| NM_182755 | | | | ZNF438 | | | -3.19 | | 1.88E-02 | | | | | 2.87 | | ns. | | |
| 🟋 Known to be upregulated of UVB irradiation utilizing TLDA as previously reported [[1](#_ENREF_1)]  (J) Defined as the JNK-regulated genes [[2](#_ENREF_2)]  ns. Not significant  🟋 The expression level of genes in UVB-irradiated containing active photolyase samples decreased or increased to the level of control i.e. there was no statistically significant difference comparing these samples to non-irradiated ones. | | | | | | | | | | | | | | | | | | |

**References**

1. Emri E, Miko E, Bai P, Boros G, Nagy G, et al. (2015) Effects of non-toxic zinc exposure on human epidermal keratinocytes. Metallomics.

2. Gazel A, Banno T, Walsh R, Blumenberg M (2006) Inhibition of JNK promotes differentiation of epidermal keratinocytes. J Biol Chem 281: 20530-20541.
